# Suzuki-Type Cross-Coupling Reaction of Unprotected 3-Iodoindazoles with Pinacol Vinyl Boronate: An Expeditive C-3 Vinylation of Indazoles under Microwave Irradiation

**DOI:** 10.3390/molecules23082051

**Published:** 2018-08-16

**Authors:** Gonzalo Vera, Benjamín Diethelm, Claudio A. Terraza, Gonzalo Recabarren-Gajardo

**Affiliations:** 1 Departamento de Farmacia, Facultad de Química, Pontificia Universidad Católica de Chile, Casilla 306, Avda. Vicuña Mackenna 4860, Macul 7820436, Santiago, Chile; glvera@uc.cl (G.V.); bmdiethelm@uc.cl (B.D.); 2 Departamento de Química Orgánica, Facultad de Química, Pontificia Universidad Católica de Chile, Casilla 306, Avda. Vicuña Mackenna 4860, Macul 7820436, Santiago, Chile; cterraza@uc.cl; 3 Centro Interdisciplinario de Neurociencias, Pontificia Universidad Católica de Chile, Marcoleta 391, Santiago 8330024, Santiago, Chile

**Keywords:** vinylation, Suzuki cross-coupling, 3-iodoindazole, 3-vinylindazole, microwave synthesis

## Abstract

Herein we report an expeditive C-3 vinylation of unprotected 3-iodoindazoles under microwave irradiation. Ten C-5 substituted 3-vinylindazole derivatives, nine of them novel, were synthesized through this method, which proceeds in moderate to excellent yields starting from C-5 substituted 3-iodoindazole derivatives. In all cases, the C-3 vinylated derivative was the only isolated product. This methodology allows access to 3-vinylated indazoles selectively and directly without the need of *N*-protection. 3-Vinylindazoles could be interesting synthetic intermediates allowing access to biologically active molecules.

## 1. Introduction

**Scheme 1:**
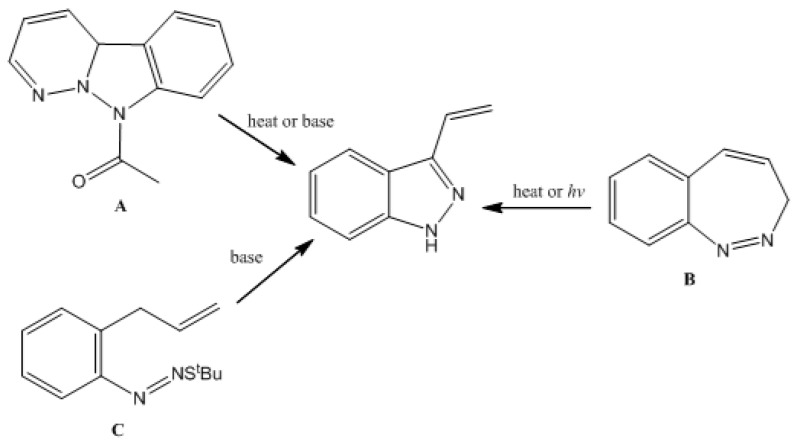
First reported syntheses of 3-vinylindazole: (**A**) Igeta’s work [8]; (**B**) Tsuchiya group’s work [10] and (**C**) report of the Petrillo group [11].

The indazole ring is a heterocycle which, being an isostere of the indole ring, exhibits a very interesting medicinal chemistry potential. Having shown a broad variety of biological activities, this heterocycle could lead to the development of several pharmaceutical products [1]. Interestingly, the indazole nucleus is scarcely found in Nature, and to date, only three natural products containing this unit have been isolated: nigellicine [2], nigeglanine [3], and nigellidine [4]. For these reasons, the development of synthetic methodologies aimed at this aromatic moiety is an active area of research. Our research group has been conducting efforts to develop novel 5-HT_6_ receptor antagonists [5,6,7]. In this sense, we are very interested in preparing 5-HT_6_ receptor ligands based on the indazole nucleus, where a key intermediate in the envisaged synthetic route of these serotonergic ligands is 3-vinylindazole. The synthesis of this parent compound has been reported in some works, the first of them carrying out its preparation from synthetically complex precursors. In the 70s Igeta et al. obtained 3-vinylindazole through both thermal and basic decomposition of 1-(pyridazino [1,6-b]indazol-9(4aH)-yl)ethan-1-one with poor to moderate yields [8,9]. Another work from Tsuchiya’s group reported the synthesis of 3-vinyl indazole through a rearrangement of 3*H*-diazepines by thermo- or photo-stimulation in outstanding yields (95% and 90% respectively) [10]. Furthermore, Petrillo et al. described the preparation of 3-vinylindazole from *o*-allylphenylazosulfides in basic media with excellent yield [11] (Scheme 1).

**Scheme 2:**
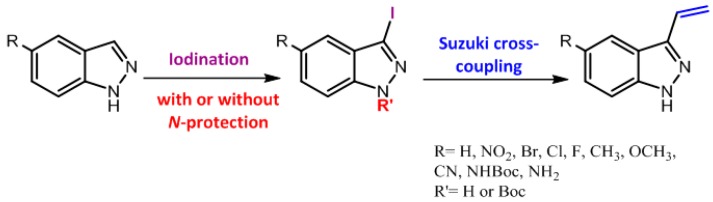
Synthetic pathway to prepare 5-R-3-vinylindazole derivatives.

However, despite the excellent reported yields, none of these works have dealt with obtaining C-5-substituted 3-vinylindazole derivatives. On the other hand, cross-coupling reactions have been widely studied as a way to perform palladium or nickel catalyzed vinylation reactions with aryl halide (or pseudohalide) substrates [12,13,14]. Nevertheless, despite the vast number of developed transition metal-catalyzed cross-coupling reactions, only a small fraction of these accommodate the attachment of a simple vinyl unit, with an aryl halide being used as the substrate an overwhelming number of times more than the heteroaryl halides [12]. On the other hand, in an outstanding work by Buchwald et al. the authors describe the arylation and heteroarylation of unprotected, nitrogen-rich heterocycles through a Suzuki cross-coupling reaction [15]. However, in this work the authors do not investigate the vinylation reaction over these substrates. In this sense, some reports have used cross-coupling reactions to functionalize the indazole ring as an attractive alternative to cyclization reactions. The most notable works in this area have been performed by Collot et al. [16,17,18,19,20,21,22]. In one of such works, the authors report the synthesis of a C-3 methyl acrylate-substituted indazole ring through a Heck cross-coupling reaction in order to easily obtain azatryptamines [18]. However, in the case of our compounds the presence of the ester function does not allow the easy preparation of our scaffold. More recently, the preparation of some 3-vinylindazole derivatives using cross-coupling reactions has been reported [23,24,25,26]. In one of them, the parent 3-vinylindazole was prepared in 60% yield from 3-bromo-1*H*-indazole via Stille coupling, the main drawback being the toxicity of the tin compounds used [23]. In another work by Sampson et al. the authors managed to prepare one 6-functionalized 3-vinylindazole with very good yield, but in a long time of reaction, via Suzuki cross-coupling in the context of the discovery of Polo-Like kinase 4 inhibitors as antitumoral agents [24]. In another example, the authors achieved the synthesis of 3-vinyl-1*H*-indazol-5-carboxylic acid methyl ester from the corresponding *N*-unprotected 3-iodoindazole via Suzuki cross-coupling under microwave irradiation at 160 °C in 40% yield [25]. Finally, in a patent by Li et al. the authors managed to prepare a highly functionalized 3-vinylindazole derivative in 39% yield using three equivalents of 4,4,5,5-tetramethyl-2-vinyl-1,3,2-dioxaborolane and 10 mol% of catalyst under conventional heating in a very long time of reaction [26]. Additionally, amongst the cross-couplings reactions, the Suzuki-Miyaura coupling is arguably the most important C-C-coupling method employed due to its broad substrate scope, high level of functional group tolerance, and, with modern catalysts, high turnover rates [15,27]. Considering all this gathered background, we set ourselves the goal of evaluating the vinylation reaction directly on the indazole nucleus through a Suzuki cross-coupling reaction and explore the influence of the electronic properties of the C-5 substituent on the indazole nucleus on the course of the reaction. Consequently, the following reaction sequence was proposed (Scheme 2).

## 2. Results and Discussions

### 2.1. Synthesis of Starting Materials

**Scheme 3:**
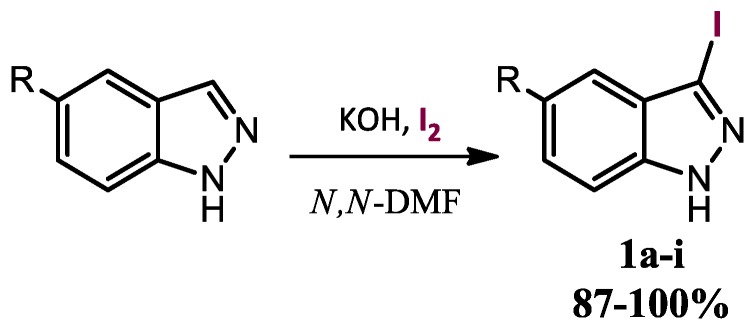
Iodination reaction of commercial indazole.

According to Scheme 2, the first step in the envisaged pathway involved the preparation of the 3-iodinated derivatives of several C-5 substituted commercial indazoles in a *N*,*N*-dimethyl-formamide (*N*,*N*-DMF) solution under standard basic conditions [16,18,28]. Thus, the corresponding 5-R-3-iodoindazole derivatives were obtained in excellent yields ranging from 87% to 100% (Scheme 3). An exception was 5-amino-3-iodoindazole; due to the high insolubility of the substrate in the reaction solvent, the product was obtained through a three-step sequence with a moderate final yield (49%). Said sequence involved: *N*-Boc protection of the aniline functionality (90%), iodination at C-3 (54%) and quantitative *N*-deprotection. Attempts to improve the yield of this iodination were infructuous.

**Scheme 4:**
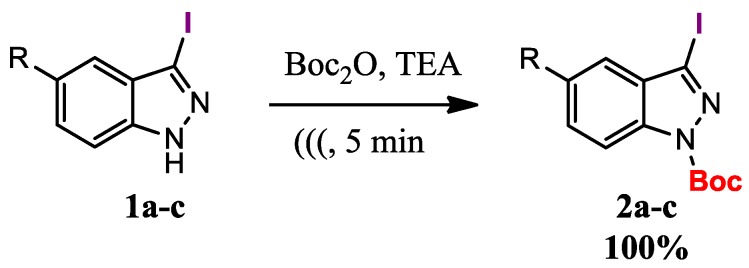
*N_1_*-protection of 3-iodoindazole derivatives using ultrasound irradiation.

As a previous step to the vinylation reaction assay, *N*_1_-protection of 3-iodoindazole derivatives with di-*4*-butyl dicarbonate (Boc_2_O) under ultrasound irradiation in basic media at room temperature was performed. The *N*_1_-Boc-3-iodoindazole products were quantitatively obtained in less than 5 min (Scheme 4).

This experiment was carried out in accordance with works that report that the 3-arylation of indazole requires the previous *N_1_*-protection of the NH group due to the competing *N_1_*-arylation reaction [16,29]. It is noteworthy to mention that the previously reported *N*-protection procedures apply ultrasound irradiation on aliphatic or aromatic amine substrates [30], which are inherently more basic than *N*-heterocyclic rings. On the other hand, experimental procedures that employ indazole as a substrate for *N*-protection use standard basic conditions, namely DMAP/Et_3_N in acetonitrile [18,31]. Thus, to our knowledge, this is the first report that employs ultrasound energy to *N*-protect the indazole nucleus.

### 2.2. Optimisation

Once *N*-protection had been carried out, the obtained *N_1_*-protected-3-iodoindazole (**2a**) was employed in the vinylation reaction assay with pinacol vinyl boronate under microwave irradiation, using Pd(PPh_3_)_4_ as a catalyst in basic media, replicating conditions described by Hopkins [29] (Table 1). The first attempt led to the intended product in a moderate yield of 60%, with the concomitant and expected loss of the Boc protecting group [32,33] (entry 1).

Afterwards, in an attempt to increase the yield of the reaction and to verify whether the vinylation reaction exhibits a similar behaviour to the arylation reaction previously described in the literature, the described vinylation reaction was tested employing non-*N*-protected 3-iodoindazole (**1a**) as a substrate under the same experimental conditions. Interestingly, under these conditions the yield of 3-vinylindazole increased to 75% (entry 2). Next, in order to explore whether the catalyst system could influence the course of the reaction or the ratio of the products, we also performed a vinylation reaction assay under microwave irradiation employing 3-iodoindazole (**1a**) and replicating previous conditions (namely, presence of aqueous base, 1,4-dioxane as solvent, and two equivalents of pinacol vinyl boronate) using different catalysts (entry 3–6) (Table 1). All the employed catalytic systems led to the C-3-vinylation of 3-iodoindazole **1a** without the need of *N*-protection. Nevertheless, only PdCl_2_(PPh_3_)_2_ led to the complete conversion of the 3-iodoindazole substrate **1a,** yielding a 50% of 3-vinylindazole **3a** (entry 5). In all other cases, a significant amount of unreacted 3-iodoindazole **1a** was recovered, carrying to a poor reaction yield. Thus, use of the Pd(OAc)_2_/XantPhos system led to a greater substrate recovery percentage than product yield (32% vs. 20% for **1a** and **3a** respectively) (entry 3, Table 1). On the other hand, Pd(OAc)_2_ afforded similar yields for both the vinylation product and the substrate (34% of **3a** vs. 35% of **1a**) but nevertheless the product yield was poor (entry 4, Table 1). Likewise, the nickel-based catalyst showed no improvement on the yield of the vinylation reaction; although it was possible to obtain the final reaction product, it was in a very low yield, and an important amount of the iodinated precursor was recovered (44% vs. 20% for **1a** and **3a** respectively) (entry 6, Table 1). Therefore, even though the obtainment of the product, using various catalytic systems, reveals the versatility and tolerability of the vinylation reaction in face of a lack of *N*-protection, the results achieved were poorer than those obtained when Pd(PPh_3_)_4_ was employed as a catalyst. In order to determine the optimum equivalents of Suzuki coupling partner for the vinylation reaction we proceeded to assay the reaction of compound **1a** with one or three equivalents of pinacol vinyl boronate (entries 7 and 8). When one equivalent of the coupling partner was employed, 3-vinyl indazole was obtained in 48% yield, recovering 33% of 3-iodoindazole (entry 7). Alternatively, when we used three equivalents of the boron compound, 59% of 3-vinylindazole, without traces of 3-iodo-indazole, was obtained (entry 8). As an additional exploration of the reaction conditions, we carried out two more changes in the reaction’s parameters. First, we assayed the precursor (**1a**) increasing the temperature to 180 °C while keeping all other conditions unchanged. Under this temperature the C-3 vinylation product was the only isolated compound, but a slightly lower yield was observed than at 120 °C (68%, entry 9 vs. 75%, entry 2). We also carried out the vinylation of **1a** at 120 °C in a longer reaction time, resulting in a poorer product yield (34%, entry 8 vs. 75%, entry 2). Overall, the best conditions for the vinylation reaction were those initially employed in this work, that is, two equivalents of the boron coupling partner, tetrakis triphenylphosphine palladium (0) as catalyst and microwave irradiation at 120 °C for 40 min (Table 1). Under these experimental conditions, the vinylation reaction proceeds with quantitative conversion of the reactant yielding 3-vinylindazole (**3a**) after chromatographic purification in a very good yield (entry 2, Table 1).

### 2.3. Scope

In order to evaluate the scope of these conditions, the vinylation reaction was tested on several *N*-unprotected 5-*R*-3-iodoindazoles encompassing a wide variety of both electron-withdrawing and electron-donating substituents (entries 2, 4 and 6–12, Table 2). 

In all cases, the isolated product was the C-3-vinyl derivative, while no other competing product was detected. The obtained yields range from moderate to excellent (see Table 2). Despite the variability in electronic effects of the C-5 substituents, no correlation was observed either in the course of the reaction or in the yield of the reaction products, except for the opposing cases of the nitro (electron-withdrawing) (entry 2) and amino (electron-donating) (entry 12) groups (Table 2). Additionally, a few other *N*-protected derivatives was assayed in order to shed light on the unexpected behavior of this vinylation (entries 1, 3 and 5). In this sense, 3-iodo-5-nitroindazole, a more reactive substrate towards Suzuki cross coupling, was assayed with *N*-Boc protection under the optimized experimental conditions (entry 1). Surprisingly, the vinylation product was formed in a very poor yield (13%) with 3-iodo-5-nitro-1*H*-indazole being isolated as a co-product of the reaction in an 86% yield as consequence of *N*-deprotection reaction. However, by conducting a more exhaustive search of the literature, we find that a similar observation for the nitro analogue was previously reported Guillaumet et al. where the Suzuki arylation coupling of *N*-Boc 3-iodo-7-nitroindazole fails to give the C-3 arylation product, but the unprotected 3-iodo-7-nitro-1*H*-indazole is isolated in 95% [32]. Conversely, when unprotected 3-iodo-5-nitroindazole (**1b**) was employed, the vinylation product was obtained in excellent yield (87%) (entry 2). It seems to be that the nitro indazole derivatives are highly reactive towards *N*-deprotection. This is not surprising since the powerful electron withdrawing nitro group is at the right place for stabilisation of the anion generated by the Boc deprotection. One tentative explanation is that the *N*-deprotection for this analogue is so favored that occurs before the vinylation reaction and the side products generated by the cleavage of the Boc group poison the Pd-catalytic system [34]. However, more experiments to corroborate this hypothesis would be necessary [35].

Additionally, we used the *N*-Boc protected 5-bromo derivative (**2c**) (entry 3), and its unprotected counterpart (**1c**) (entry 4), both under the same conditions, obtaining the C-3-vinylation product in 43% and 60% yield respectively. Noteworthy that in the experiment with the *N*-Boc protected 5-bromo derivative (**2c**), we achieved to isolate a 5% of the 5-bromo-3-vinyl-*N*-protected derivative as only coproduct of the reaction, without observation of the C-5 vinylation product. Finally, as an additional proof of the reaction behavior, we performed the vinylation reaction with *N*-Boc protected 5-methoxy derivative (**2d**) (entry 5), and its unprotected counterpart (**1d**) (entry 6). The first example (entry 5) led to the isolation of the C-3-vinylation product in 46% yield and an additional 26% yield of the *N*-protected C-3-vinylation compound. The reaction with the unprotected precursor (entry 6) conduct to the preparation of the *N*-unprotected C-3 vinylation product in a 58% yield, corroborating the results obtained with the three previous examples (Table 1). Interestingly, compared to nitro compound, in the examples with 5-bromo and 5-methoxy derivatives the *N*-deprotection reaction possibly occurs after the vinylation reaction. Considering the absence of a strong electron-withdrawing group in these latter examples this hypothesis seems to be not very surprisingly. This is tentatively supported by the recovery of the *N*-Boc vinylated products for the 5-Br and 5-OMe analogues, isolated in 5% and 26% yield, respectively. The 5-OMe group being electron donating will be the most resistant to *N*-deprotection and this is supported by the higher amount of recovered *N*-Boc vinylated product vs. the 5-Br.

Next, with the aim of determining whether microwave heating was essential to the success of the reaction, we also performed the vinylation reaction of the unprotected derivatives 3-iodoindazole (**1a**), 5-nitro-3-iodoindazole (**1b**), 5-methoxy-3-iodoindazole (**1d**), 5-fluoro-3-iodoindazole (**1e**), and 5-methyl-3-iodoindazole (**1g**) under conventional heating conditions (entries 13–17, Table 2). In most cases, the reaction leads to the formation of the corresponding C-3 vinylation products (only fluoro derivative led to recovery of precursor (63% yield) and no isolation of the vinylation product), but in lower yields (except for **3g**) and a longer reaction time than the procedure performed under microwave heating. The case of 5-methoxy-3-iodoindazole **1g** is particularly noteworthy, as the work of Hopkins shows no arylation reaction of this 3-iodoindazole derivative under conventional heating (DME at 80 °C) [29]. However, the present conditions led, with an increment in heating temperature of only 20 °C, to the obtainment of the Suzuki cross-coupling product **3g** in a very good (75%) yield.

The NMR analyses support the purity of the different synthesized compounds, the case of the amine derivatives being an exception (see supplementary materials). Both the ^1^H- and ^13^C-NMR spectra show a certain degree of impurities which could not be minimized despite the purification efforts carried out. On the other hand, the analyses of mass spectrometry support the success of the syntheses in all cases.

To the best of our knowledge, this is the first systematic study performing a vinylation cross-coupling reaction on the C-5 substituted indazole derivatives without *N*-protection of the indazole ring and without detection of the any other competing product.

## 3. Materials and Methods

### 3.1. General Information

All organic solvents used for the synthesis were of analytical grade. All reagents used were purchased from Sigma-Aldrich (St. Louis, MO, USA), Merck (Darmstadt, Germany) or AK Scientific (Union City, CA, USA) and were used as received. Melting points were determined on a Stuart Scientific SMP30 apparatus (Bibby Scientific Limited, Stone, UK) and are uncorrected. NMR spectra were recorded on anAvance III HD 400 instrument (Bruker, Billerica, MA, USA) at 400 MHz for ^1^H and 100 MHz for ^13^C-NMR spectra were recorded in DMSO-d_6_ unless otherwise indicated, using the solvent signal as reference. The chemical shifts are expressed in ppm (δ scale) downfield from tetramethylsilane (TMS), and coupling constants values (*J*) are given in Hertz. The IR spectra were obtained on a Bruker Vector 22 spectrophotometer using KBr discs. High resolution mass spectra were obtained on high resolution mass spectrometer Exactive™ Plus Orbitrap (ThermoFisher Scientific, Bremen, Germany) using electron impact ionization. Column chromatography was performed on Merck silica gel 60 (70–230 mesh). Thin layer chromatographic separations were performed on Merck silica gel 60 (70–230 mesh) chromatofoils. Ultrasonic reactions were performed on an ultrasound device Branson 2210R-MT Ultrasonic Cleaner (Marshall Scientific, Danbury, CT, USA). Suzuki cross coupling reactions were performed on a microwave oven Monowave 300 (Anton Paar, Graz, Austria).

### 3.2. Syntheses and Characterization Procedures

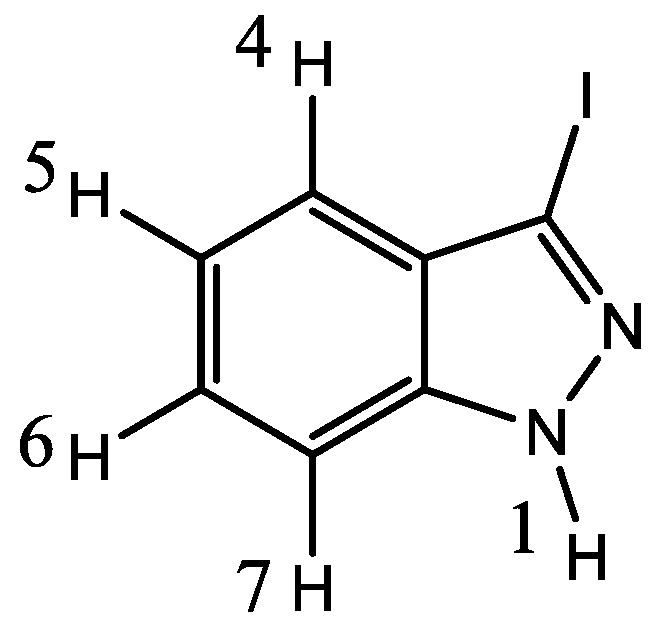



*3-Iodo-1H-indazole* (**1a**). 3-Iodoindazoles were obtained by direct iodination of commercial indazoles by the method previously described by Bocchi [28] with slight modifications. A solution of 1*H*-indazole (3 g, 25.4 mmol), iodine (12.7 g, 50.03 mmol) and potassium hydroxide (5.34 g, 95.25 mmol) in DMF (7 mL) was stirred for 3 h at room temperature. The reaction was quenched by dilution with saturated solution of sodium bisulfite (150 mL) and a precipitated was formed. The precipitated was filtered over vacuum and washed with water (3 × 30 mL). The solid was left to dry at 30 °C in a vacuum oven overnight obtaining 6.17 g of a pale yellow solid. Yield: 100%; m.p.: 136–138 °C (lit.: [36] 134–136 °C); IR (KBr) ν (cm^−1^): 3086 (NH); 424 (C-I). ^1^H-NMR δ (ppm): 13.50 (1H, s, H-1); 7.55 (1H, d, *J* = 8.6 Hz, H-7); 7.45–7.40 (2H, m, H-6 and H-4); 7.19 (1H, dd, *J* = 7.5 Hz, H-5). ^13^C-NMR δ (ppm): 140.41; 127.22; 126.79; 121.23; 120.39; 110.51; 93.49; HRMS calculated for C_7_H_5_IN_2_: 243.9497, Found: 243.9499.

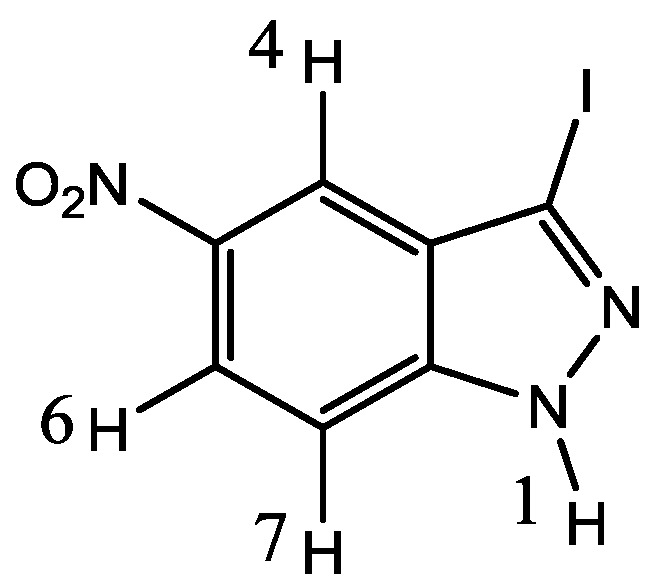



*3-Iodo-5-nitro-1H-indazole* (**1b**). Prepared from 5-nitro-1*H*-indazole (3 g, 18.4 mmol), iodine (9.0 g, 35.46 mmol), potassium hydroxide (3.87 g, 69.00 mmol) and DMF (7 mL) to give 5.31 g of a pale yellow solid. Yield: 100%; m.p.: 216–217 °C (lit.: [22] 214 °C); IR (KBr) ν (cm^−1^): 3200 (NH); 1528 (NO_2_ asymmetrical); 1335 (NO_2_ symmetrical); 424 (C-I). ^1^H-NMR δ (ppm): 14.13 (1H, s, H-1); 8.30 (1H, d, *J* = 1.8 Hz, H-4); 8.23 (1H, dd, *J* = 9.2 and 2.1 Hz, H-6); 7.75 (1H, d, *J* = 9.2, H-7). ^13^C-NMR δ (ppm): 142.99; 142.52; 126.77; 122.42; 118.57; 112.18; 97.67; HRMS calculated for C_7_H_4_IN_3_O_2_: 288.9348, Found: 288.9353.

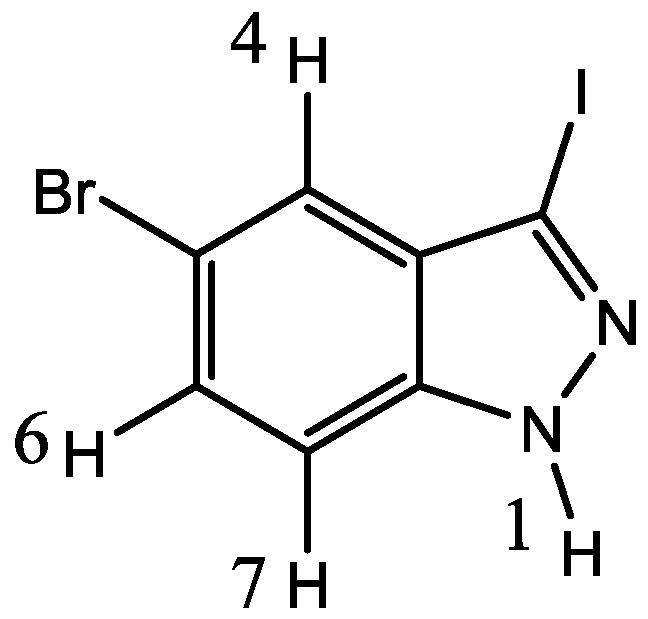



*5-Bromo-3-iodo-1H-indazole* (**1c**). Prepared from 5-bromo-1*H*-indazole (1 g, 5.07 mmol), iodine (2.5 g, 9.85 mmol), potassium hydroxide (1.07 g, 19.06 mmol) and DMF (4 mL) to give 1.63 g of a pale yellow solid. Yield: 100%; m.p.: 209–210 °C (lit.: [22] 196 °C); IR (KBr) ν (cm^−1^): 3124 (NH); 424 (C-I and C-Br). ^1^H-NMR δ (ppm): 13.69 (1H, s, H-1); 7.60 (1H, s, H-4); 7.57–7.50 (2H, m, H-6 and H-7). ^13^C-NMR δ (ppm): 139.24; 129.98; 128.46; 122.59; 113.46; 112.74; 92.47; HRMS calculated for C_7_H_4_BrIN_2_: 321.8603, Found: 321.8617.

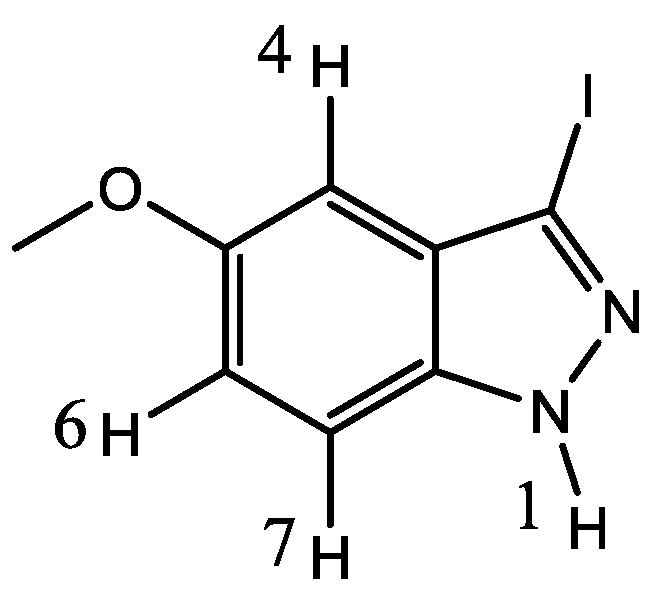



*3-Iodo-5-methoxy-1H-indazole* (**1d**). Prepared from 5-methoxy-1*H*-indazole (0.2 g, 1.35 mmol), iodine (0.7 g, 2.76 mmol), potassium hydroxide (284 mg, 5.06 mmol) and DMF (3 mL) to give 0.34 g of a pale brown solid. Yield: 90%; m.p.: 162–163 °C (lit.: [22] 178–179 °C); IR (KBr) ν (cm^−1^): 3185 (NH); 1288 (C-O); 424 (C-I). ^1^H-NMR δ (ppm): 13.36 (1H, s, H-1); 7.46 (1H, d, *J* = 8.9 Hz, H-7); 7.08 (1H, d, *J* = 8.8 Hz, H-6); 6.76 (1H, s, H-4); 3.82 (3H, s, OCH_3_). ^13^C-NMR δ (ppm): 154.71; 136.16; 127.13; 119.51; 111.74; 99.24; 92.25; 55.45; HRMS calculated for C_8_H_7_IN_2_O: 273.9603, Found: 273.9599.

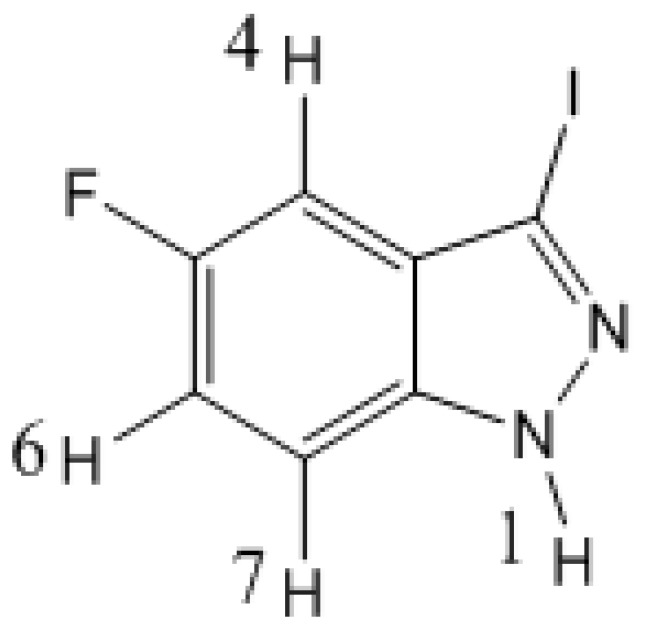



*5-Fluoro-3-iodo-1H-indazole* (**1e**). Prepared from 5-fluoro-1*H*-indazole (0.2 g, 1.47 mmol), iodine (0.75 g, 2.95 mmol), potassium hydroxide (0.31 g, 5.52 mmol) and DMF (3 mL) to give 0.33 g of a pale brown solid. Yield: 87%; m.p.: 158–159 °C (lit.: [37] 166 °C); IR (KBr) ν (cm^−1^): 3171 (NH); 1165 (C-F); 424 (C-I). ^1^H-NMR δ (ppm): 13.61 (1H, s, H-1); 7.61 (1H, dd, *J* = 9.0 and 4.1 Hz, H-7); 7.32 (1H, td, *J* = 9.1 and 2.1 Hz, H-6); 7.17 (1H, dd, *J* = 8.7 and 1.7 Hz, H-4). ^13^C-NMR δ (ppm): 157.51 (d, *J* = 237.3 Hz); 137.56; 126.96 (d, *J* = 10.4 Hz); 116.84 (d, *J* = 27.5 Hz); 112.41 (d, *J* = 9.7 Hz); 104.44 (d, *J* = 24.2 Hz); 92.69 (d, *J* = 5.8 Hz); HRMS calculated for C_7_H_4_FIN_2_: 261.9403, Found: 261.9408.

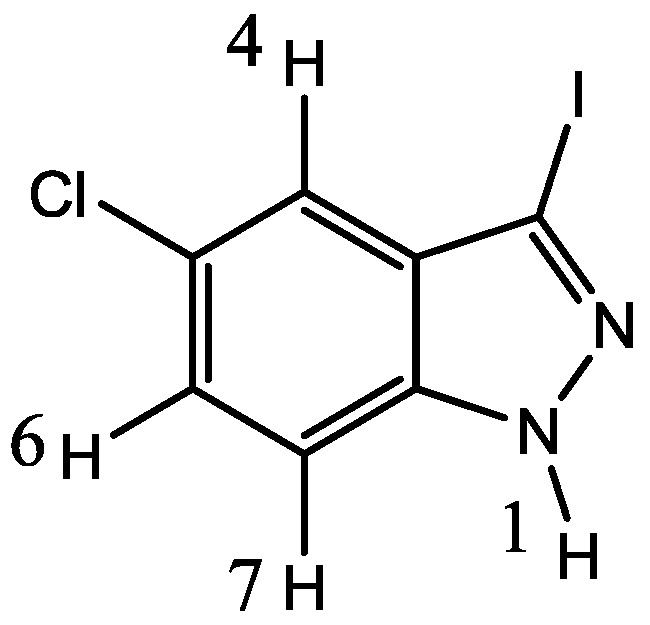



*5-Chloro-3-iodo-1H-indazole* (**1f**). Prepared from 5-chloro-1*H*-indazole (0.2 g, 1.31 mmol), iodine (0.65 g, 2.56 mmol), potassium hydroxide (275 mg, 4.90 mmol) and DMF (3 mL) to give 0.33 g of a pale brown solid. Yield: 90%; m.p.: 182–183 °C; IR (KBr) ν (cm^−1^): 3125 (NH); 710 (C-Cl); 424 (C-I). ^1^H-NMR δ (ppm): 13.69 (1H, s, H-1); 7.60 (1H, d, *J* = 8.7 Hz, H-7), 7.46–7.40 (2H, m, H-4 and H-6). ^13^C-NMR δ (ppm): 139.10; 127.82; 127.64; 125.77; 119.48; 112.51; 92.64; HRMS calculated for C_7_H_4_ClIN_2_: 277.9108, Found: 277.9116.

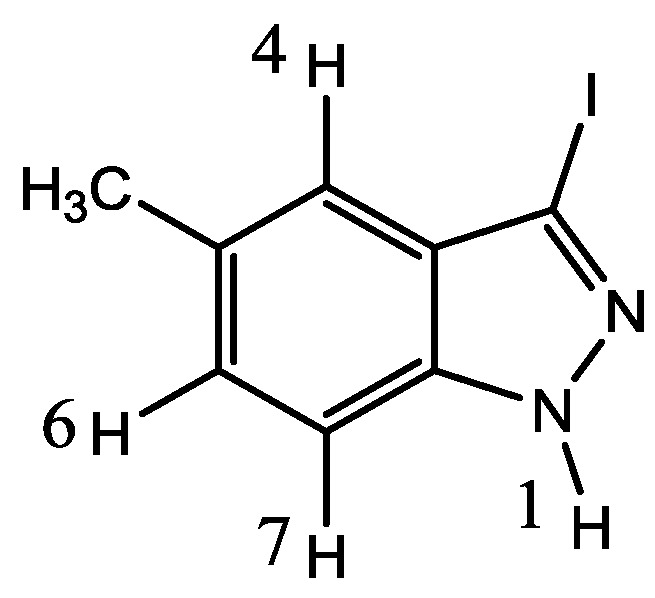



*3-Iodo-5-methyl-1H-indazole* (**1g**). Prepared from 5-methyl-1*H*-indazole (0.2 g, 1.51 mmol), iodine (0.75 g, 2.95 mmol), potassium hydroxide (318 mg, 5.67 mmol) and DMF (3 mL) to give 0.36 g of a pale brown solid. Yield: 93%; m.p.: 159–160 °C; IR (KBr) ν (cm^−1^): 3186 (NH); 2916 (C-H); 424 (C-I). ^1^H-NMR δ (ppm): 13.37 (1H, s, H-1); 7.44 (1H, d, *J* = 8.2 Hz, H-7); 7.25 (1H, d, *J* = 8.1 Hz, H-6); 7.18 (1H, s, H-4); 2.41 (3H, s, CH_3_). ^13^C-NMR δ (ppm): 139.12; 130.43; 129.33; 127.10; 119.24; 110.31; 92.59; 20.91; HRMS calculated for C_8_H_7_IN_2_: 257.9654, Found: 257.9659.

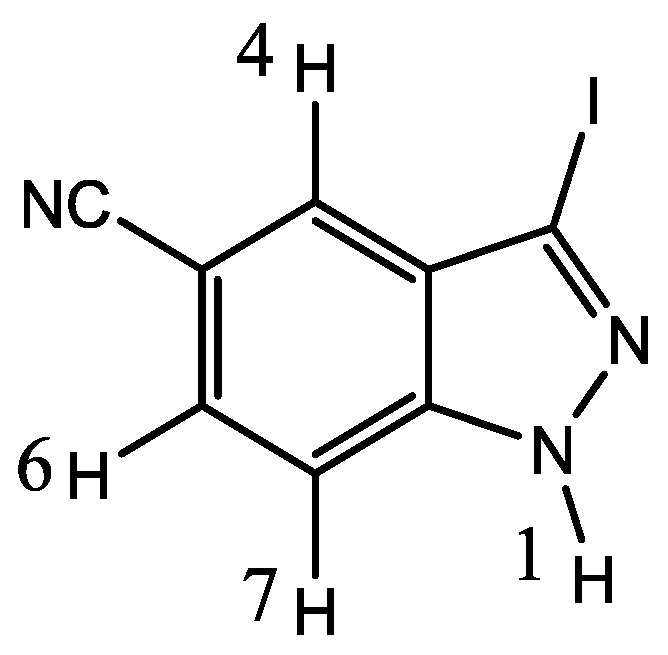



*3-Iodo-1H-indazole-5-carbonitrile* (**1h**). Prepared from 1*H*-indazole-5-carbonitrile (0.2 g, 1.4 mmol), iodine (0.7 g, 2.76 mmol), potassium hydroxide (295 mg, 5.26 mmol) and DMF (3 mL) to give 0.34 g of a white solid. Yield: 91%; m.p.: 206–207 °C; IR (KBr) ν (cm^−1^): 3340 (NH); 2283 (CN); 424 (C-I). ^1^H-NMR δ (ppm): 13.99 (1H, s, H-1); 8.04 (1H, s, H-7); 7.73 (2H, s, H-4 and H-6). ^13^C-NMR δ (ppm): 141.53; 129.27; 127.39; 126.73; 119.30; 112.25; 103.73; 95.36. HRMS calculated for C_8_H_4_IN_3_: 268.9450, Found: 268.9455.

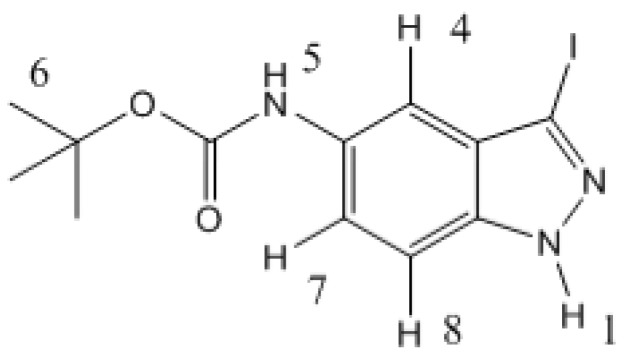



*tert-Butyl (3-iodo-1H-indazol-5-yl)carbamate* (**1i**). Prepared from *tert*-butyl(1-*H*-indazol-5-yl)carbamate (0.35 g, 1.5 mmol), iodine (0.75 g, 2.95 mmol), potassium hydroxide (316 mg, 5.63 mmol) and DMF (3 mL). The brown solid obtained was purificated by column chromatography on silica gel using *n*-hexane/ethyl acetate 1:1 *v*/*v* to obtain 0.29 g of pure product as brown crystalline plates. Yield: 54%; m.p.: 181–182 °C; IR (KBr) ν (cm^−1^): 3348 (NH); 1750 (C=O); 1157 (C-O); 424 (C-I). ^1^H-NMR δ (ppm): 13.35 (1H, s, H-1); 9.40 (1H, s, H-5); 7.70 (1H, s, H-4); 7.44 (1H, d, *J* = 8.9 Hz, H-8); 7.38 (1H, d, *J* = 8.9 Hz, H-7); 1.50 (9H, s, H-6). ^13^C-NMR δ (ppm): 153.05; 136.82; 133.63; 126.91; 120.89; 110.71; 107.38; 92.78; 79.04; 28.19; HRMS calculated for C_12_H_14_IN_3_O_2_: 359.0131, Found: 359.0128.

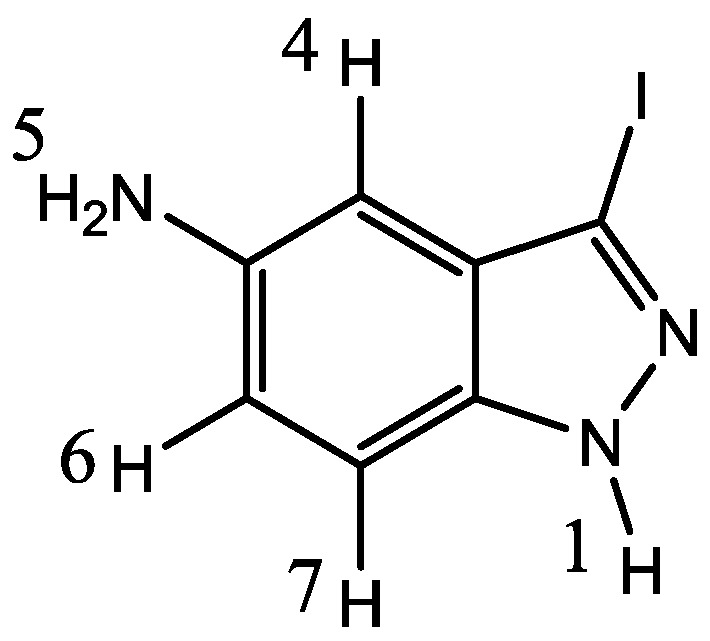



*3-Iodo-1H-indazol-5-amine* (**1j**). To a solution of tert-butyl (3-iodo-1-*H*-indazol-5-yl)carbamate (0.2 g, 0.56 mmol) (**1i**) and trifluoroacetic acid (5 mL, 65.34 mmol) in CH_2_Cl_2_ (5 mL) was stirred vigorously for 2 h at room temperature. After stirring, the reaction was neutralized to pH = 7 using NaOH 1M and the organic layer was extracted with ethyl acetate (3 × 20 mL) The combined organic layers were dried over anhydrous sodium sulfate and removal of the solvent under vacuum afforded a 144 mg of pure product as a black solid. Yield: 100%; m.p.: 178–179 °C; IR (KBr) ν (cm^−1^): 3375 (NH); 424 (C-I). ^1^H-NMR δ (ppm): 13.07 (1H, s, H-1); 7.25 (1H, d, *J* = 8.8 Hz, H-7), 6.85 (1H, dd, *J* = 8.8 and 1.7 Hz, H-6); 6.44 (1H, s, H-4); 5.01 (2H, br s, H-5). ^13^C-NMR δ (ppm): 143.46; 134.79; 127.96; 119.39; 110.91; 99.83; 90.59; HRMS calculated for C_7_H_6_IN_3_: 258.9606, Found: 258.9602.

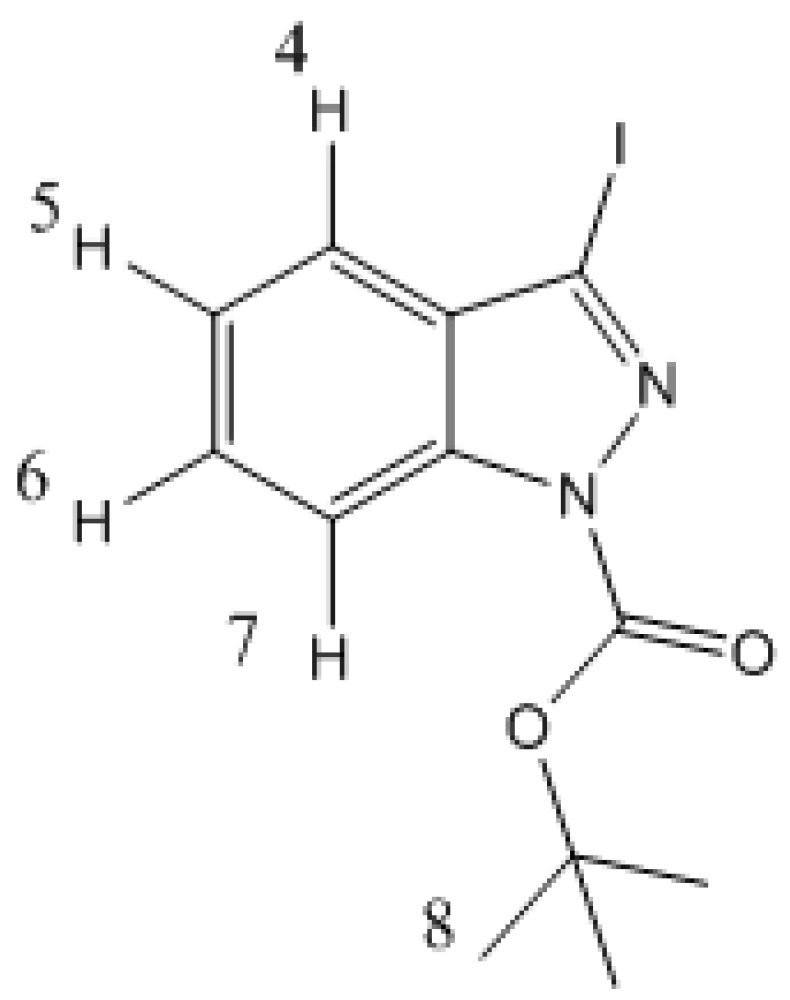



*tert-Butyl 3-iodo-1H-indazole-1-carboxylate* (**2a**). A mixture of 3-iodo-1*H*-indazole (0.2 g, 0.82 mmol), di-*tert*-butyldicarbonate (0.2 g, 0.92 mmol) and triethylamine (1 mL) were put under ultrasonic irradiation for 10 min. The resulted solution was neutralized using HCl 1M and then extracted with dichloromethane (3 × 30 mL). The combined organic layers were dried with anhydrous sodium sulfate and removal of the solvent under vacuum afforded a pure product as a pale yellow crystals. Yield: 100%; m.p.: 93–95 °C; IR (KBr) ν (cm^−1^): 1728 (C=O); 1150 (C-O); 424 (C-I). ^1^H-NMR (CDCl_3_) δ (ppm): 8.09 (1H, d, *J* = 8.5 Hz, H-7); 7.55 (1H, t, *J* = 7.8 Hz, H-4); 7.46 (1H, d, *J* = 7.9 Hz, H-6); 7.33 (1H, t, *J* = 7.6 Hz, H-5); 1.71 (9H, s, CH_3_). ^13^C-NMR δ (ppm): 148.35; 139.59; 130.17; 129.98; 124.21; 121.96; 114.56; 102.95; 85.48; 28.18; HRMS calculated for C_12_H_13_IN_2_O_2_: 344.0022, Found: 344.0016.

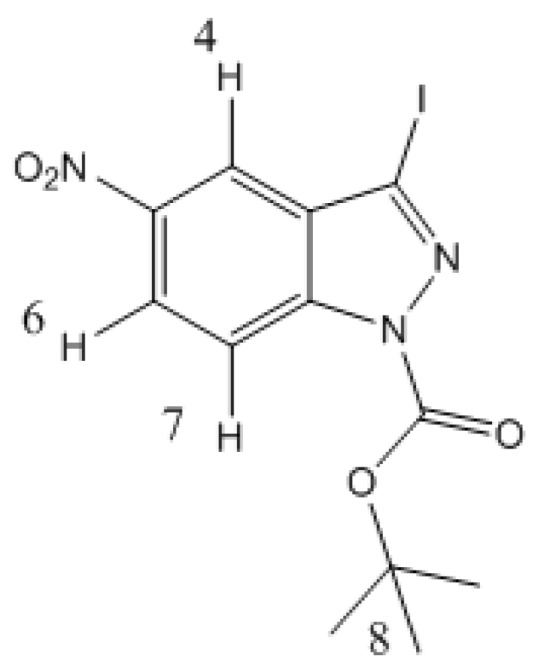



*tert-Butyl 3-iodo-5-nitro-1H-indazole-1-carboxylate* (**2b**). Prepared from 3-iodo-5-nitro-1*H*-indazole (0.2 g, 0.69 mmol), di-*tert*-butyldicarbonate (0.17 g, 0.78 mmol) and triethylamine (1 mL) to give 0.27 g of a pale yellow solid. Yield: 100%; m.p.: 144–145 °C; IR (KBr) ν (cm^−1^): 1744 (C=O); 1528 (NO_2_ asymmetrical); 1381 and 1342 (NO_2_ symmetrical); 1258 (C-O); 617 (C-I). ^1^H-NMR δ (ppm): 8.49 (1H, br s, H-4); 8.32 (1H, br s, H-7); 8.25 (1H, br s, H-6); 1.66 (9H, s, CH_3_). ^13^C-NMR δ (ppm): 147.11; 144.13; 141.57; 129.78; 124.83; 118.31; 115.39; 105.88; 86.23; 27.56; HRMS calculated for C_12_H_12_IN_3_O_4_: 388.9872, Found: 388.9871.

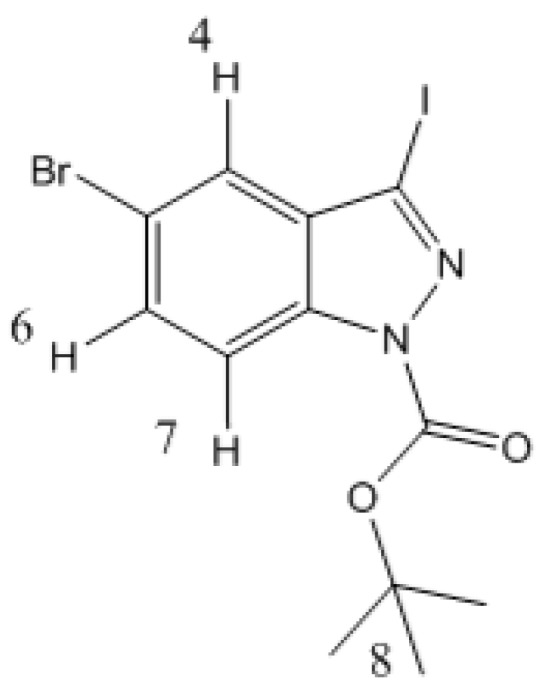



*tert-Butyl 5-bromo-3-iodo-1H-indazole-1-carboxylate* (**2c**). Prepared from 5-bromo-3-iodo-1*H*-indazole (0.2 g, 0.62 mmol), di-*tert*-butyldicarbonate (0.15 g, 0.69 mmol) and triethylamine (1 mL) to give 0.27 g of a pale yellow solid. Yield: 100%; m.p.: 152–153 °C; IR (KBr) ν (cm^−1^): 1750 (C=O); 1157 (C-O); 424 (C-I and C-Br). ^1^H-NMR (CDCl_3_) δ (ppm): 8.01 (1H, d, *J* = 8.8 Hz, H-7); 7.66 (1H, d, *J* = 9.2 Hz, H-6); 7.64 (1H, s, H-4); 1.64 (9H, s, CH_3_). ^13^C-NMR δ (ppm): 148.14; 138.67; 133.15; 131.89; 124.69; 117.56; 116.15; 101.11; 86.13; 28.23; HRMS calculated for C_12_H_12_BrIN_2_O_2_: 421.9127, Found: 421.9128.

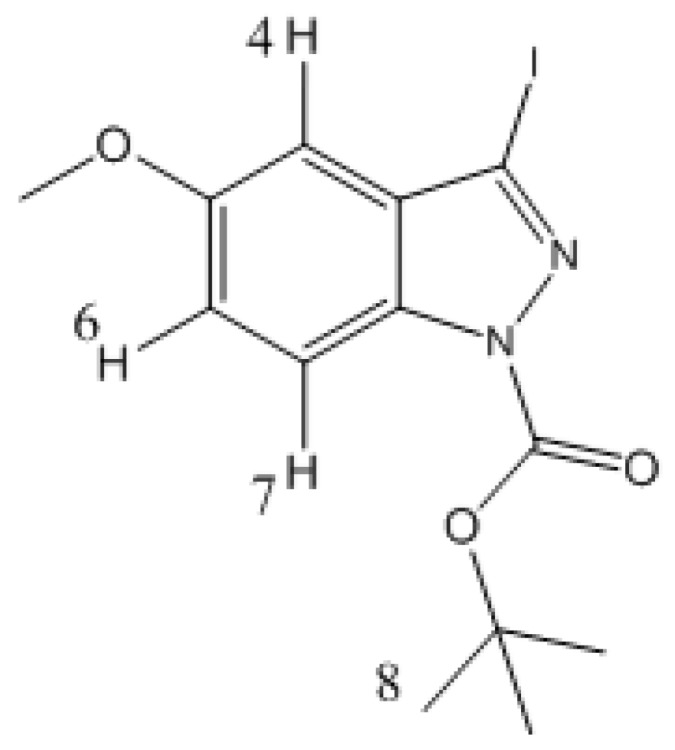



*tert-Butyl 3-iodo-5-methoxy-1H-indazole-1-carboxylate* (**2d**). Prepared from 3-iodo-5-methoxy-1*H*-indazole (0.1 g, 0.365 mmol), di-*tert*-butyldicarbonate (0.08 g, 0.366 mmol) and triethylamine (1 mL) to give 119 mg of a pale yellow solid. Yield: 88%; m.p.: 130–131 °C; IR (KBr) ν (cm^−1^): 1740 (C=O); 1298 (H_3_C-O); 1157 (C-O); 424 (C-I). ^1^H-NMR (CDCl_3_) δ (ppm): 8.01 (1H, d, *J* = 9.1 Hz, H-7); 7.22 (1H, dd, *J* = 9.1 and 2.4 Hz, H-6); 6.82 (1H, d, *J* = 2.2 Hz, H-4); 3.93 (3H, s, OCH_3_); 1.74 (9H, s, CH_3_). ^13^C-NMR δ (ppm): 157.902; 148.32; 134.85; 130.95; 121.12; 115.58; 102.20; 101.71; 85.45; 55.86; 28.21; HRMS calculated for C_13_H_15_IN_2_O_3_: 374.0127, Found: 374.0118.

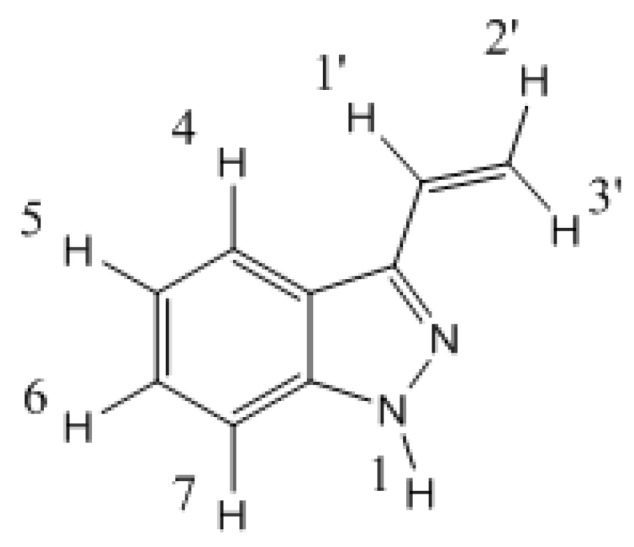



*3-Vinyl-1H-indazole* (**3a**). *Method a*: A mixture of 3-iodoindazole (0.2 g, 0.82 mmol), 2 equivalents of vinyl boronic acid pinacol ester (0.27 mL, 1.62 mmol), tetrakis triphenylphosphine palladium (52 mg, 0.045 mmol), an aqueous solution of sodium carbonate 2N (2 mL) and 1,4-dioxane (7 mL), were placed in a microwave glass tube and purged with nitrogen. The closed tube was placed under microwave irradiation to 120 °C for 40 min. After irradiation was completed, the reaction was stopped by dilution using 50 mL of brine. The organic layer was extracted with ethylacetate (3 × 45 mL) and the combined organic layers were dried over anhydrous sodium sulfate. Removal of the solvent under vacuum afforded a brown oil crude residue. The oil was purified by column chromatography on silica gel (hexane/ethylacetate 7:3) to yield 89 mg of white crystalline plates. Yield: 75%.

*Method b***:** Prepared from *tert*-butyl 3-iodo-1*H*-indazole-1-carboxylate (0.2 g, 0.58 mmol), 2 eq. of vinyl boronic acid pinacol ester (0.27 mL, 1.62 mmol), tetrakistriphenylphosphine palladium (52 mg, 0.045 mmol), an aqueous solution of sodium carbonate 2N (2 mL) and dioxane (7 mL) using microwave method described above to obtain 50 mg of a crystalline plates: Yield: 60%.

*Method c*: Prepared from 3-iodoindazole (0.2 g, 0.82 mmol), 1 equivalent of vinyl boronic acid pinacol ester (0.14 mL, 0.823 mmol), tetrakistriphenylphosphine palladium (52 mg, 0.045 mmol), an aqueous solution of sodium carbonate 2N (2 mL) and dioxane (7 mL) using microwave method described above to obtain 58 mg of a crystalline plates. Yield: 48%.

*Method d*: Prepared from 3-iodoindazole (0.2 g, 0.82 mmol), 3 equivalents of vinyl boronic acid pinacol ester (0.42 mL, 2.47 mmol), tetrakistriphenylphosphine palladium (52 mg, 0.045 mmol), an aqueous solution of sodium carbonate 2N (2 mL) and dioxane (7 mL) using the previously described microwave method to obtain 70 mg of a crystalline plates. Yield: 59%.

*Method e*: Prepared from 3-iodoindazole (0.2 g, 0.82 mmol), 2 equivalents of vinyl boronic acid pinacol ester (0.28 mL, 1.62 mmol), palladium (II) acetate (5 mol%, 9 mg, 0.041 mmol), Xantphos (5 mol%, 24 mg, 0.041 mmol) an aqueous solution of potassium phosphate 1M (2 mL) and dioxane (7 mL) using microwave method described above to obtain 23 mg of a crystalline plates. Yield: 20%.

*Method f*: Prepared from 3-iodoindazole (0.2 g, 0.82 mmol), 2 equivalents of vinyl boronic acid pinacol ester (0.28 mL, 1.62 mmol), palladium (II) acetate (5 mol%, 9 mg, 0.041 mmol), an aqueous solution of sodium carbonate 2N (2 mL) and dioxane (7 mL) using microwave method described above to obtain 40 mg of a crystalline plates. Yield: 34%.

*Method g*: Prepared from 3-iodoindazole (0.2 g, 0.82 mmol), 2 equivalents of vinyl boronic acid pinacol ester (0.28 mL, 1.62 mmol), bis(triphenylphosphine)palladium (II) dichloride (5 mol%, 29 mg, 0.041 mmol), triphenylphosphine (5 mol%, 11 mg, 0.041 mmol), an aqueous solution of sodium carbonate 2N (2 mL) and dioxane (7 mL) using microwave method described above to obtain 58 mg of a crystalline plates. Yield: 49%.

*Method h*: Prepared from 3-iodoindazole (0.2 g, 0.82 mmol), 2 equivalents of vinyl boronic acid pinacol ester (0.28 mL, 1.62 mmol), [1,2-bis(diphenylphosphino)ethane]dichloro nickel (II) (5 mol%, 22 mg, 0.041 mmol), ethylenebis(diphenylphosphine) (5 mol%, 16 mg, 0.041 mmol), an aqueous solution of potassium phosphate 1M (2 mL) and dioxane (7 mL) using microwave method described above to obtain 24 mg of a crystalline plates. Yield: 20%.

*Method i*: Prepared from 3-iodoindazole (0.2 g, 0.82 mmol), 2 equivalents of vinyl boronic acid pinacol ester (0.28 mL, 1.62 mmol), tetrakistriphenylphosphine palladium (52 mg, 0.045 mmol), an aqueous solution of sodium carbonate 2N (2 mL) and dioxane (7 mL) using microwave method described above, but at a reaction temperature of 180 °C, to obtain 80 mg of a crystalline plates. Yield: 68%.

*Method j:* Prepared from 3-iodoindazole (0.2 g, 0.82 mmol), 2 equivalents of vinyl boronic acid pinacol ester (0.28 mL, 1.62 mmol), tetrakistriphenylphosphine palladium (52 mg, 0.045 mmol), an aqueous solution of sodium carbonate 2N (2 mL) and dioxane (7 mL) using microwave method described above, but with a reaction time of 60 min, to obtain 40 mg of a crystalline plates. Yield: 34%.

*Method k*: Prepared from 3-iodoindazole (0.1 g, 0.41 mmol), 2 eq. of vinyl boronic acid pinacol ester (0.1 mL, 0.81 mmol), tetrakistriphenylphosphine palladium (26 mg, 0.023 mmol), an aqueous solution of sodium carbonate 2N (1 mL) and dioxane (3.5 mL) using method a described above, but employing conventional heating, to obtain 26 mg of a crystalline plates: Yield: 44%.

m.p.: 104–105 °C (lit.: [11] 115.5–116.5 °C); IR (KBr) ν (cm^−1^): 3047 (NH); 1620 (C=C). ^1^H-NMR (CDCl_3_) δ (ppm): 11.32 (1H, s, H-1); 7.95 (1H, d, *J* = 8.2 Hz, H-7); 7.48 (1H, d, *J* = 8.4 Hz, H-4); 7.40 (1H, t, *J* = 7.6 Hz, H-6); 7.21 (1H, t, *J* = 7.5 Hz, H-5); 7.13 (1H, dd, *J_trans_* = 18.0 Hz and *J*_cis_ = 11.5 Hz, H-1′); 6.16 (1H, d, *J*_trans_ = 18.0 Hz, H-3′); 5.58 (1H, d, *J*_cis_ = 11.4 Hz, H-2′). ^13^C-NMR (CDCl_3_) δ (ppm): 144.18; 141.59; 128.90; 127.03; 121.48; 121.14; 121.00; 117.18; 110.30; HRMS calculated for C_9_H_8_N_2_: 144.0687, Found: 144.0681.

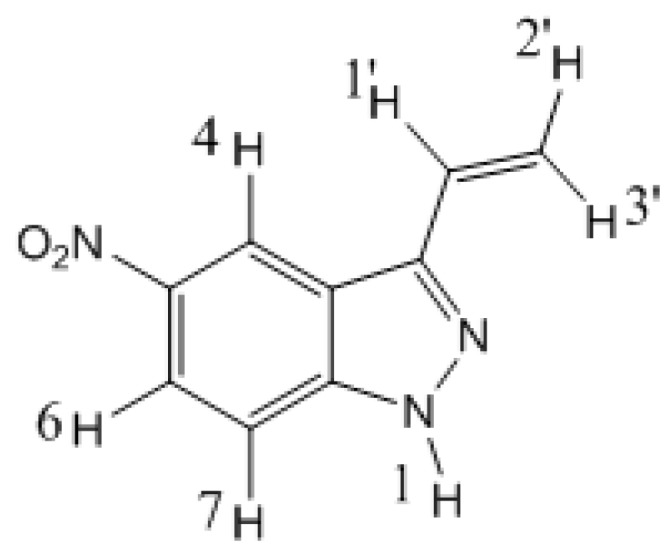



*5-Nitro-3-vinyl-1H-indazole* (**3b**). *Method a*: Prepared from 3-iodo-5-nitro-1*H*-indazole (0.2 g, 0.69 mmol), 2 equivalents of vinyl boronic acid pinacol ester (0.23 mL, 1.35 mmol), tetrakis- triphenylphospine palladium (52 mg, 0.045 mmol), aqueous solution of sodium carbonate 2N (2 mL) and dioxane (7 mL), to give 0.113 g of a pale brown solid. Yield: 87%.

*Method b*: Prepared from *tert*-butyl 3-iodo-5-nitro-1*H*-indazole-1-carboxylate (0.2 g, 0.514 mmol), 2 eq. of vinyl boronic acid pinacol ester (0.17 mL, 1.03 mmol), tetrakistriphenylphosphine palladium (35 mg, 0.03 mmol), an aqueous solution of sodium carbonate 2N (2 mL) and dioxane (7 mL) using microwave method described above to obtain 13 mg of a pale brown solid: Yield: 13%.

*Method c*: Prepared from 3-iodo-5-nitro-1*H*-indazole (0.18 g, 0.62 mmol), 2 equivalents of vinyl boronic acid pinacol ester (0.21mL, 1.24 mmol), tetrakistriphenylphospine palladium (52 mg, 0.045 mmol), aqueous solution of sodium carbonate 2N (2 mL) and dioxane (7 mL) using method a described above, but employing conventional heating, to give 24 mg of pale brown solid. Yield: 21%.

m.p.: 166–168 °C; IR (KBr) ν (cm^−1^): 3179 (NH); 1620 (C=C); 1528 (NO_2_ asymmetrical); 1335 (NO_2_ symmetrical). ^1^H-NMR δ (ppm): 13.75 (1H, s, H-1); 8.93 (1H, s, H-4); 8.20 (1H, d, *J* = 8.6 Hz, H-7); 7.71 (1H, d, *J* = 9.2 Hz, H-6); 7.14 (1H, dd, *J_trans_* = 17.8 Hz and *J*_cis_ =11.4 Hz, H-1′); 6.19 (1H, d, *J*_trans_ = 17.8 Hz, H-3′); 5.58 (1H, d, *J*_cis_ = 11.4 Hz, H-2′).^13^C-NMR δ (ppm): 145.12; 142.97; 141.89; 127.65; 121.14; 119.61; 118.21; 117.95; 111.31; HRMS calculated for C_9_H_7_N_3_O_2_: 189.0538, Found: 189.0535.

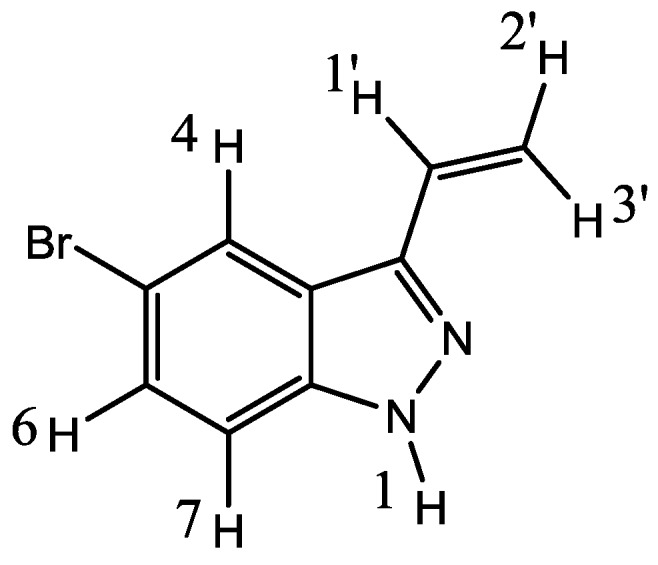



*5-Bromo-3-vinyl-1H-indazole* (**3c**). *Method a*: Prepared from 5-bromo-3-iodo-1*H*-indazole (0.2 g, 0.621 mmol), 2 equivalent of vinyl boronic acid pinacol ester (0.21 mL, 1.24 mmol), tetrakis- triphenylphospine palladium (52 mg, 0.045 mmol), aqueous solution of sodium carbonate 2N (2 mL) and dioxane (7 mL), to give 82 mg of a pale yellow solid. Yield: 60%.

*Method b*: Prepared from tert-butyl 5-bromo-3-iodo-1*H*-indazole-1-carboxylate (0.2 g, 0.47 mmol), 2 eq. of vinyl boronic acid pinacol ester (0.16 mL, 0.96 mmol), tetrakistriphenylphosphine palladium (28 mg, 0.024 mmol), an aqueous solution of sodium carbonate 2N (2 mL) and dioxane (7 mL) using microwave method described above to obtain 45 mg of a pale yellow solid: Yield: 43%.

m.p.: 250–251 °C; IR (KBr) ν (cm^−1^): 3112 (NH); 1689 (C=C); 424 (C-Br). ^1^H-NMR (CDCl_3_) δ (ppm): 11.68 (1H, s, H-1); 8.03 (1H, s, H-4); 7.44 (1H, d, *J* = 8.8 Hz, H-7); 7.32 (1H, d, *J* = 8.8 Hz, H-6); 7.01 (1H, dd, *J*_trans_ = 18.0 Hz and *J*_cis_ = 11.5 Hz, H-1′); 6.07 (1H, d, *J*_trans_ = 18.0 Hz, H-3′); 5.57 (1H, d, *J*_cis_ = 11.5 Hz, H-2′). ^13^C-NMR (CDCl_3_) δ (ppm): 143.45;140.15; 130.21; 128.12; 123.48; 122.54; 117.83; 114.66; 111.77; HRMS calculated for C_9_H_7_BrN_2_: 221.9793, Found: 221.9797.

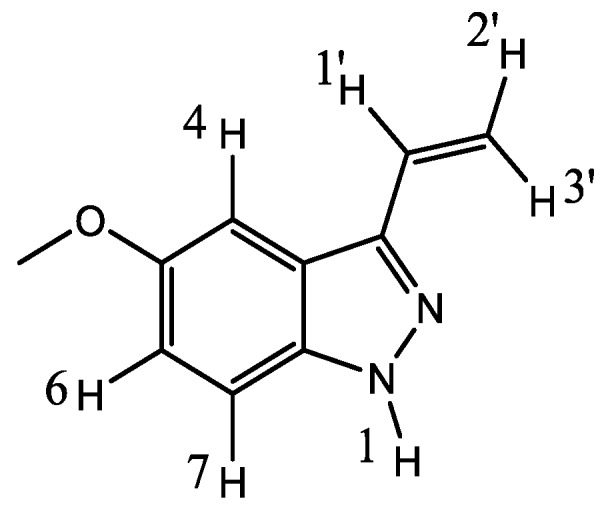



*5-Methoxy-3-vinyl-1H-indazole* (**3d**). *Method a*: Prepared from 3-iodo-5-methoxy-1*H*-indazole (0.2 g, 0.73 mmol), 2 equivalents of vinyl boronic acid pinacol ester (0.24 mL, 1.43 mmol), tetrakis- triphenylphospine palladium (52 mg, 0.045 mmol), aqueous solution of sodium carbonate 2N (2 mL) and dioxane (7 mL), to give 74 mg of a pale yellow solid. Yield: 58%.

*Method b*: Prepared from *tert*-butyl 3-iodo-5-methoxy-1*H*-indazole-1-carboxylate (0.135 g, 0.361 mmol), 2 eq. of vinyl boronic acid pinacol ester (0.12 mL, 0.72 mmol), tetrakistriphenylphosphine palladium (21 mg, 0.018 mmol), an aqueous solution of sodium carbonate 2N (2 mL) and dioxane (7 mL) using microwave method described above to obtain 29 mg of a pale yellow solid. Yield: 46%.

*Method c*: Prepared from 3-iodo-5-methoxy-1*H*-indazole (0.2 g, 0.73 mmol), 2 equivalents of vinyl boronic acid pinacol ester (0.24 mL, 1.43 mmol), tetrakistriphenylphosphine palladium (52 mg, 0.045 mmol), an aqueous solution of sodium carbonate 2N (2 mL) and dioxane (7.0 mL) using method described above, but employing conventional heating, to obtain 96 mg of a pale yellow solid. Yield: 75%.

m.p.: 88–89 °C; IR (KBr) ν (cm^−1^): 3155 (NH); 1635 (C=C); 1219 (C-O). ^1^H-NMR δ (ppm): 12.95 (1H, s, H-1); 7.44 (1H, d, *J* = 8.9 Hz, H-7); 7.33 (1H, s, H-4); 7.06–6.96 (2H, m, H-6 and H-1’); 6.02 (1H, d, *J*_trans_ = 18.0 Hz, H-3′); 5.41 (1H, d, *J*_cis_ = 11.5 Hz, H-2′); 3.82 (1H, s, OCH_3_). ^13^C-NMR δ (ppm): 154.50; 141.71; 137.02; 129.38; 120.70; 118.05; 114.90; 111.47; 100.02; 55.50; HRMS calculated for C_10_H_10_N_2_O: 174.0793, Found: 174.0788.

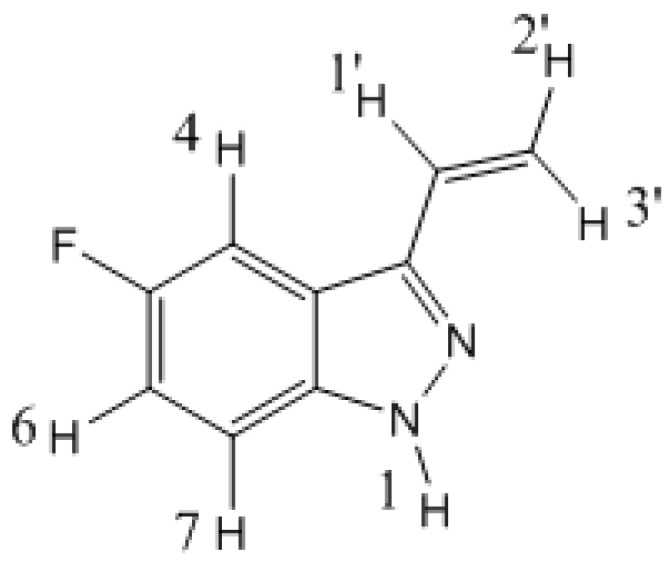



*5-Fluoro-3-vinyl-1H-indazole* (**3e**). *Method a*: Prepared from 5-fluoro-3-iodo-1*H*-indazole (0.2 g, 0.763 mmol), 2 equivalents of vinyl boronic acid pinacol ester (0.26 mL, 1.54 mmol), tetrakis- triphenylphospine palladium (52 mg, 0.045 mmol), aqueous solution of sodium carbonate 2N (2 mL) and dioxane (7 mL), to give 58 mg of a pale yellow solid. Yield: 47%.

*Method b*: Prepared from 5-fluoro-3-iodo-1*H*-indazole (0.08 g, 0.305 mmol), 2 eq. of vinyl boronic acid pinacol ester (0.075 mL, 0.61 mmol), tetrakistriphenylphosphine palladium (17 mg, 0.015 mmol), an aqueous solution of sodium carbonate 2N (1 mL) and dioxane (3.5 mL) using method a described above, but employing conventional heating, to obtain 50 mg of the precursor 5-fluoro-3-iodo-1*H*-indazole: Yield of recovery: 63%.

m.p.: 104–105 °C; IR (KBr) ν (cm^−1^): 3163 (NH); 1636 (C=C); 1118 (C-F). ^1^H-NMR (CDCl_3_) δ (ppm): 11.73 (1H, s, H-1); 7.52 (1H, dd, *J* = 8.9 and 1.7 Hz, H-4); 7.41 (1H, dd, *J* = 9.0 and 4.2 Hz, H-7); 7.17 (1H, td, *J* = 8.9 and 2.0 Hz, H-6); 7.05 (1H, dd, *J_trans_* = 18.0 Hz and *J*_cis_ = 11.5 Hz, H-1′); 6.05 (1H, d, *J*_trans_ = 18.0 Hz, H-3′); 5.56 (1H, d, *J*_cis_ = 11.5 Hz, H-2′). ^13^C-NMR (CDCl_3_) δ (ppm): 158.39 (d, *J* = 238.8 Hz), 144.04 (d, *J* = 5.5 Hz), 138.52, 128.38, 121.01 (d, *J* = 10.0 Hz), 117.25, 111.49 (d, *J* = 9.7 Hz), 105.21 (d, *J* = 24.1 Hz); HRMS calculated for C_9_H_7_FN_2_: 162.0593, Found: 162.0590.

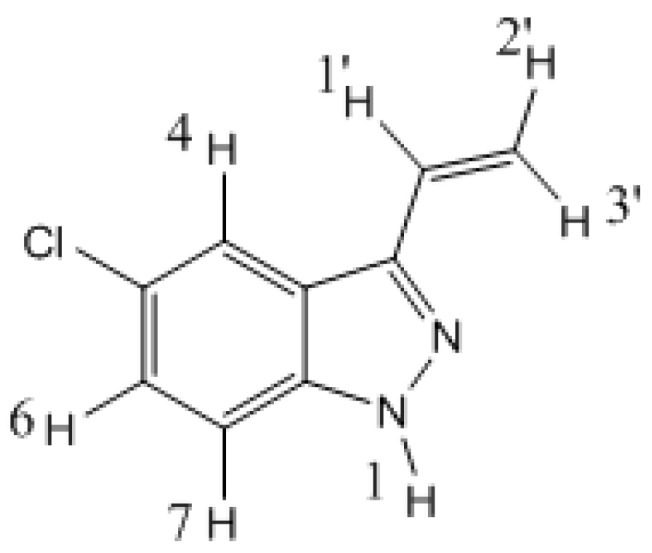



*5-Chloro-3-vinyl-1H-indazole* (**3f**). Prepared from 5-chloro-3-iodo-1*H*-indazole (0.2 g, 0.722 mmol), 2 equivalents of vinyl boronic acid pinacol ester (0.24 mL, 1.42 mmol), tetrakistriphenylphospine palladium (52 mg, 0.045 mmol), aqueous solution of sodium carbonate 2N (2 mL) and dioxane (7 mL), to give 77 mg of a yellow solid. Yield: 60%; m.p.: 131–132 °C; IR (KBr) ν (cm^−1^): 3181 (NH); 1628 (C=C); 649 (C-Cl). ^1^H-NMR δ (ppm): 13.28 (1H, s, H-1); 8.05 (1H, s, H-4); 7.56 (1H, d, *J* = 8.8 Hz, H-7); 7.37 (1H, d, *J* = 8.7 Hz, H-6); 7.01 (1H, dd, *J*_trans_ = 18.0 Hz and *J*_cis_ = 11.5 Hz, H-1′); 6.08 (1H, d, *J*_trans_ = 18.0 Hz, H-3′); 5.46 (1H, d, *J*_cis_ = 11.5 Hz, H-2′). ^13^C-NMR δ (ppm): 142.00; 139.72; 128.57; 126.56; 125.45, 121.23, 119.60, 116.29, 112.17; HRMS calculated for C_9_H_7_ClN_2_: 178.0298, Found: 178.0296.

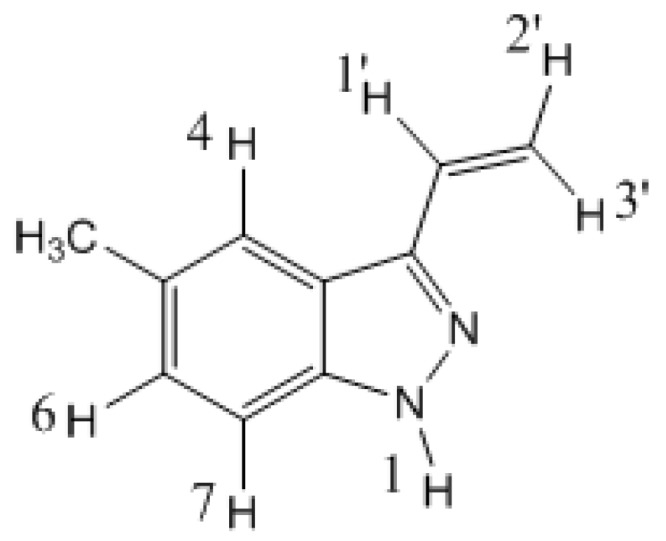



*5-Methyl-3-vinyl-1H-indazole* (**3g**). *Method a*: Prepared from 3-iodo-5-methyl-1*H*-indazole (0.2 g, 0.775 mmol), 2 equivalent of vinyl boronic acid pinacol ester (0.26 mL, 1.54 mmol), tetrakis- triphenylphospine palladium (52 mg, 0.045 mmol), aqueous solution of sodium carbonate 2N (2 mL) and dioxane (7 mL), to give 60 mg of a pale brown solid. Yield: 49%.

*Method b*: Prepared from 3-iodo-5-methyl-1*H*-indazole (0.095 g, 0.368 mmol), 2 equivalents of vinyl boronic acid pinacol ester (0.090 mL, 0.736 mmol), tetrakistriphenylphosphine palladium (21 mg, 0.018 mmol), an aqueous solution of sodium carbonate 2N (1 mL) and dioxane (3.5 mL) using method a described above, but employing conventional heating, to obtain 10 mg of a pale brown solid. Yield: 17%.

m.p.: 88–89 °C; IR (KBr) ν (cm^−1^): 3179 (NH); 2916 (C-H); 1628 (C=C). ^1^H-NMR (CDCl_3_) δ (ppm): 11.63 (1H, s, H-1); 7.72 (1H, s, H-4); 7.37 (1H, d, *J* = 8.5 Hz, H-7); 7.24–7.11 (2H, m, H-1′ and H-6); 6.16 (1H, d, *J*_trans_ = 18.0 Hz, H-3′); 5.57 (1H, d, *J*_cis_ = 11.4 Hz, H-2′); 2.50 (3H, s, CH_3_). ^13^C-NMR (CDCl_3_) δ (ppm):143.28; 140.28; 130.84; 129.02; 128.96; 121.38; 119.96; 116.69; 110.12; 21.58; HRMS calculated for C_10_H_10_N_2_: 158.0844, Found: 158.0840.

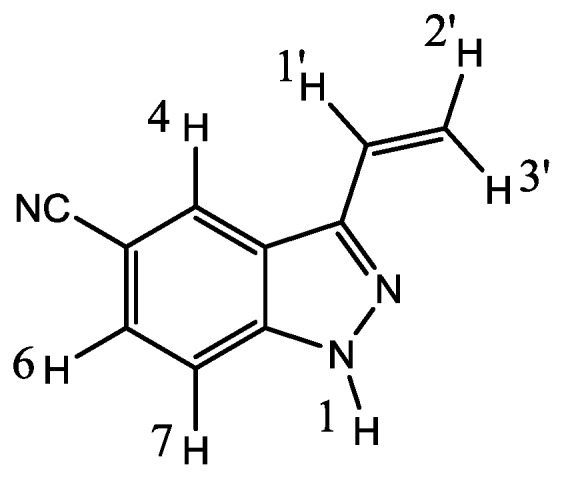



*5-Cyano-3-vinyl-1H-indazole* (**3h**). Prepared from 5-cyano-3-iodo-1*H*-indazole (0.2 g, 0.743 mmol), 2 equivalents of vinyl boronic acid pinacol ester (0.25 mL, 1.48 mmol), tetrakistriphenylphospine palladium (52 mg, 0.045 mmol), aqueous solution of sodium carbonate 2N (2 mL) and dioxane (7 mL), to give 77 mg of a white solid. Yield: 61%; m.p.: 156–157 °C; IR (KBr) ν (cm^−1^): 3209 (NH); 2222 (CN); 1612 (C=C). ^1^H-NMR δ (ppm): 13.59 (1H, s, H-1); 8.63 (1H, s, H-4), 7.68 (2H, s, H-7 and H-6); 7.04 (1H, dd, *J*_trans_ = 18.0 Hz and *J*_cis_ = 11.5 Hz, H-1′), 6.21 (1H, d, *J* = 18.0 Hz, H-3′), 5.53 (1H, d, *J* = 11.5 Hz, H-2′). ^13^C-NMR δ (ppm): 143.56; 142.01; 128.38; 128.01; 127.43; 119.99; 119.82; 117.82; 111.98; 103.29; HRMS calculated for C_10_H_7_N_3_: 169.0640, Found: 169.0637.

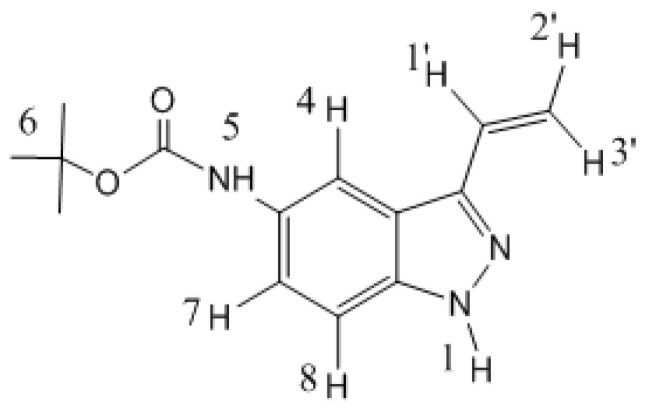



*tert-Butyl (3-vinyl-1H-indazol-5-yl)carbamate* (**3i**). Prepared from *tert*-butyl (3-iodo-1-*H*-indazol-5-yl) carbamate (0.2 g, 0.56 mmol), 2 equivalents of vinyl boronic acid pinacol ester (0.19 mL, 1.12 mmol), tetrakistriphenylphospine palladium (52 mg, 0.045 mmol), aqueous solution of sodium carbonate 2N (2 mL) and dioxane (7 mL), to give 80 mg of a White solid. Yield: 55%; m.p.: 189–190 °C; IR (KBr) ν (cm^−1^): 3356 (NH); 1700 (C=O); 1636 (C=C); 1165 (C-O). ^1^H-NMR δ (ppm): 12.96 (1H, s, H-1); 9.30 (1H, s, H-5); 8.13 (1H, s, H-4); 7.45–7.37 (2H, m, H-8 and H-7); 6.98 (1H, dd, *J_trans_* = 18.0 Hz and *J_cis_ =* 11.4 Hz, H-1′); 5.92 (1H, d, *J_trans_* = 18.0 Hz, H-3′); 5.44 (1H, d, *J*_cis_ = 11.4 Hz, H-2′); 1.49 (9H, s, H-6). ^13^C-NMR δ (ppm): 153.18; 141.83; 137.80; 133.36; 129.70; 120.33; 119.92; 114.88; 110.54; 107.98; 78.91; 28.22; HRMS calculated for C_14_H_17_N_3_O_2_: 259.1321, Found: 259.1330.

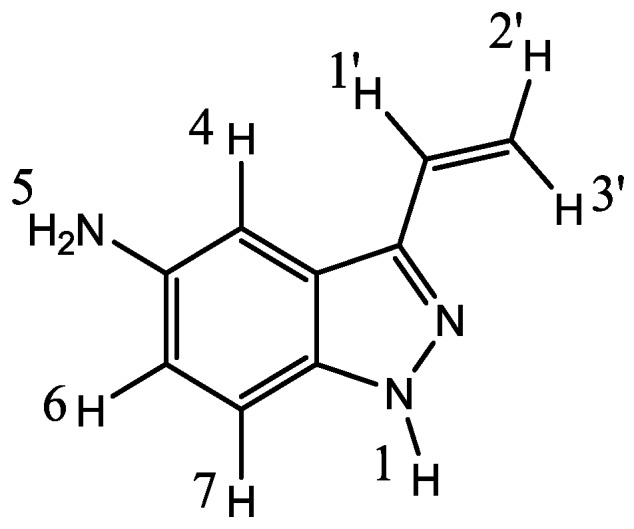



*5-Amino-3-vinyl-1H-indazole* (**3j**). *Method a*: Prepared from 5-amino-3-iodo-1*H*-indazole (**1j**, 0.2 g, 0.77 mmol), 2 equivalents of vinyl boronic acid pinacol ester (0.26 mL, 1.54 mmol), tetrakis- triphenylphospine palladium (52 mg, 0.045 mmol), aqueous solution of sodium carbonate 2N (2 mL) and dioxane (7 mL), to give 44 mg of a brown solid. Yield: 36%.

*Method b*: A solution of *tert*-butyl (3-vinyl-1*H*-indazol-5-yl) carbamate (**3i**, 145 mg, 0.4 mmol) and trifluoroacetic acid (2 mL, 26.13 mmol) in CH_2_Cl_2_ (2 mL) was stirred vigorously for 2 h at room temperature. After stirring, the reaction was neutralized to pH = 7 using NaOH 1M and the organic layer was extracted with ethyl acetate (3 × 20 mL) The combined organic layers were dried with anhydrous sodium sulfate and removal of the solvent under vacuum afforded a 80 mg of pure product as a brown solid. Yield: 100%; m.p.: 157–158 °C; IR (KBr) ν (cm^−1^): 3461 (NH); 1682 (C=O). ^1^H-NMR δ (ppm):12.64 (1H, s, H-1); 7.24 (1H, d, *J* = 8.7 Hz, H-7); 7.00 (1H, s, H-4); 6.91 (1H, dd, *J_trans_* = 18.0 and *J_cis_ =* 11.5 Hz, H-1′); 6.80 (1H, d, *J* = 8.8 Hz, H-6); 5.84 (1H, d, *J_trans_* = 18.0 Hz, H-3′); 5.34 (1H, d, *J_cis_* = 11.5 Hz, H-2′); 4.87 (2H, s, H5). ^13^C-NMR δ (ppm): 143.20; 140.39; 135.83; 130.33; 121.46; 118.02; 113.61; 110.81; 100.76; HRMS calculated for C_9_H_9_N_3_: 159.0796, Found: 159.0792.

## 4. Conclusions

We have prepared ten, nine of them novel, C-5 substituted 3-vinylindazole derivatives through an easy, simple, fast and reproducible Suzuki cross-coupling reaction between unprotected 3-iodoindazoles and pinacol vinyl boronate with yields ranging from moderate to excellent. The vinylation reaction without *N*-protection herein presented shows tolerance concerning: (i) the electronic properties of the C-5 substituent on the indazole nucleus, (ii) the use of conventional heating, and (iii) the use of diverse catalytic systems. Despite such tolerance, the best conditions for this reaction were identified as: microwave irradiation, Pd(PPh_3_)_4_ as a catalyst, 120 °C, and 40 min of reaction time. In all reported cases the main isolated product was the C-3 vinyl derivative without detection of the any other competing product. With this methodology in hand, vinylated indazoles may be directly and selectively obtained without the need of *N*-protection.

## Figures and Tables

**Table 1:**
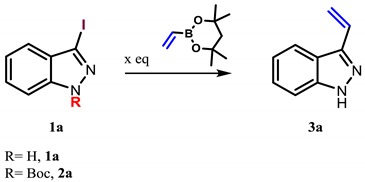
Optimization of the reaction conditions.

Entry	Reactant	Reaction Conditions	x	Time/Temp (°C)	Yield ^a^ (%)
**1**	**2a**	Pd(PPh_3_)_4_/Na_2_CO_3_	2	40 min/120	60
**2**	**1a**	Pd(PPh_3_)_4_/Na_2_CO_3_	2	40 min/120	75
**3**	**1a**	Pd(OAc)_2_/XantPhos, K_3_PO_4_	2	40 min/120	20
**4**	**1a**	Pd(OAc)_2_, Na_2_CO_3_	2	40 min/120	34
**5**	**1a**	Pd(PPh_3_)_2_Cl_2_/PPh_3_, Na_2_CO_3_	2	40 min/120	50
**6**	**1a**	NiCl_2_(dppe)/dppe, K_3_PO_4_	2	40 min/120	20
**7**	**1a**	Pd(PPh_3_)_4_/Na_2_CO_3_	1	40 min/120	48
**8**	**1a**	Pd(PPh_3_)_4_/Na_2_CO_3_	3	40 min/120	59
**9**	**1a**	Pd(PPh_3_)_4_/Na_2_CO_3_	2	40 min/180	68
**10**	**1a**	Pd(PPh_3_)_4_/Na_2_CO_3_	2	60 min/120	34

Reaction conditions: 5 mol % of catalyst, aqueous base, 1,4-dioxane, µW. ^a^ Yield corresponding to the purified products.

**Table 2:**
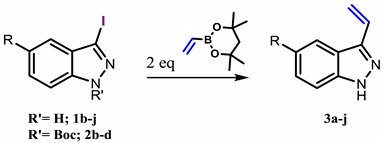
Results of the vinylation reaction of several 5-R-3-iodoindazole derivatives.

Entry	Reactant	R	R′	Heating	Product	Yield ^a^ (%)
**1**	**2b**	NO_2_	Boc	µW	**3b**	13 ^b^
**2**	**1b**	NO_2_	H	µW	**3b**	87
**3**	**2c**	Br	Boc	µW	**3c**	43 ^c^
**4**	**1c**	Br	H	µW	**3c**	60
**5**	**2d**	OCH_3_	Boc	µW	**3d**	46 ^d^
**6**	**1d**	OCH_3_	H	µW	**3d**	58
**7**	**1e**	F	H	µW	**3e**	47
**8**	**1f**	Cl	H	µW	**3f**	60
**9**	**1g**	CH_3_	H	µW	**3g**	49
**10**	**1h**	CN	H	µW	**3h**	61
**11**	**1i**	NHBoc	H	µW	**3i**	55
**12**	**1j**	NH_2_	H	µW	**3j**	36 (55) ^e^
**13**	**1a**	H	H	**conventional**	**3a**	44
**14**	**1b**	NO_2_	H	**conventional**	**3b**	21
**15**	**1d**	OCH_3_		**conventional**	**3d**	75
**16**	**1e**	F	H	**conventional**	**3e**	0
**17**	**1g**	CH_3_	H	**conventional**	**3g**	17

Reaction conditions: pinacol vinyl boronate (2 eq), Pd(PPh_3_)_4_ (5–6 mol %), Na_2_CO_3_ (2N), 1,4-dioxane, µW, 120 °C, 40 min (entries 1–12). Conventional heating, 101 °C, 3 h (entries 13–17). ^a^ Yields corresponding to the purified products. ^b^ 3-iodo-5-nitro-1*H*-indazole was obtained as the major product in 86% yield. ^c^ 5-bromo-3-vinyl-*N*-protected derivative was isolated as only coproduct in 5% yield. ^d^ *N*-boc-3-vinyl-5-methoxy-1*H*-indazole was obtained as coproduct in 26% yield. ^e^ 55% yield for 5-amino-3-vinylindazole **3j** corresponds to that obtained by deprotection of **3i**.

## Supplementary Materials

The Supplementary Materials are available online. Figure S1: ^1^H-NMR spectrum of **1a**. Figure S2: ^13^C-NMR spectrum of **1a**. Figure S3: ^1^H-NMR spectrum of **1b**. Figure S4: ^13^C-NMR spectrum of **1b**. Figure S5: ^1^H-NMR spectrum of **1c**. Figure S6: ^13^C-NMR spectrum of **1c**. Figure S7: ^1^H-NMR spectrum of **1d**. Figure S8: ^13^C-NMR spectrum of **1d**. Figure S9: ^1^H-NMR spectrum of **1e**. Figure S10: ^13^C-NMR spectrum of **1e**. Figure S11: ^1^H-NMR spectrum of **1f**. Figure S12: ^13^C-NMR spectrum of **1f**. Figure S13: ^1^H-NMR spectrum of **1g**. Figure S14: ^13^C-NMR spectrum of **1g**. Figure S15: ^1^H-NMR spectrum of **1h**. Figure S16: ^13^C-NMR spectrum of **1h**. Figure S17: ^1^H-NMR spectrum of **1i**. Figure S18: ^13^C-NMR spectrum of **1i**. Figure S19: ^1^H-NMR spectrum of **1j**. Figure S20: ^13^C-NMR spectrum of **1j**. Figure S21: ^1^H-NMR spectrum of **2a**. Figure S22: ^13^C-NMR spectrum of **2a**. Figure S23: ^1^H-NMR spectrum of **2b**. Figure S24: ^13^C-NMR spectrum of **2b**. Figure S25: ^1^H-NMR spectrum of **2c**. Figure S26: ^13^C-NMR spectrum of **2c**. Figure S27: ^1^H-NMR spectrum of **2d**. Figure S28: ^13^C-NMR spectrum of **2d**. Figure S29: ^1^H-NMR spectrum of **3a**. Figure S30: ^13^C-NMR spectrum of **3a**. Figure S31: ^1^H-NMR spectrum of **3b**. Figure S32: ^13^C-NMR spectrum of **3b**. Figure S33: ^1^H-NMR spectrum of **3c**. Figure S34: ^13^C-NMR spectrum of **3c**. Figure S35: ^1^H-NMR spectrum of **3d**. Figure S36: ^13^C-NMR spectrum of **3d**. Figure S37: ^1^H-NMR spectrum of **3e**. Figure S38: ^13^C-NMR spectrum of **3e**. Figure S39: ^1^H-NMR spectrum of **3f**. Figure S40: ^13^C-NMR spectrum of **3f**. Figure S41: ^1^H-NMR spectrum of **3g**. Figure S42: ^13^C-NMR spectrum of **3g**. Figure S43: ^1^H-NMR spectrum of **3h**. Figure S44: ^13^C-NMR spectrum of **3h**. Figure S45: ^1^H-NMR spectrum of **3i**. Figure S46: ^13^C-NMR spectrum of **3i**. Figure S47: ^1^H-NMR spectrum of **3j**. Figure S48: ^13^C-NMR spectrum of **3j**.
