# Supplementary material for: Suzuki-Type Cross-Coupling Reaction of Unprotected 3-Iodoindazoles with Pinacol Vinyl Boronate: An Expeditive C-3 Vinylation of Indazoles under Microwave Irradiation
Source: Molecules. 2018 Aug 16;23(8):2051. doi: 10.3390/molecules23082051 (PMC6222620; doi:10.3390/molecules23082051)
Supplement: Supplementary file 1 [file molecules-23-02051-s001.pdf]

Supporting Information for:

Suzuki-type Cross-Coupling Reaction of Unprotected 3-Iodoindazoles with Pinacol Vinyl Boronate: An Expeditive C-3 Vinylation of Indazoles under Microwave Irradiation.

Gonzalo Vera[1], Benjamín Diethelm[1], Claudio Terraza[2], Gonzalo Recabarren-Gajardo[1, 3, ∗]

[1]*Departamento de Farmacia, Facultad de Química, Pontificia Universidad Católica de Chile, Casilla 306, Avda. Vicuña Mackenna 4860, Macul 7820436, Santiago, Chile.*

[2]*Departamento de Química Orgánica, Facultad de Química, Pontificia Universidad Católica de Chile, Casilla 306, Avda. Vicuña Mackenna 4860, Macul 7820436, Santiago, Chile.*

[3]*Centro Interdisciplinario de Neurociencias, Pontificia Universidad Católica de Chile, Marcoleta 391, Santiago 8330024, Santiago, Chile.*

Contents:

1. NMR data of all compounds ....…………………………………..... S2-S25

∗ Corresponding author. Tel: +56-2-23541418; Fax: +56-2-23544744; e-mail: grecabarren@uc.cl

1. NMR data of all compounds

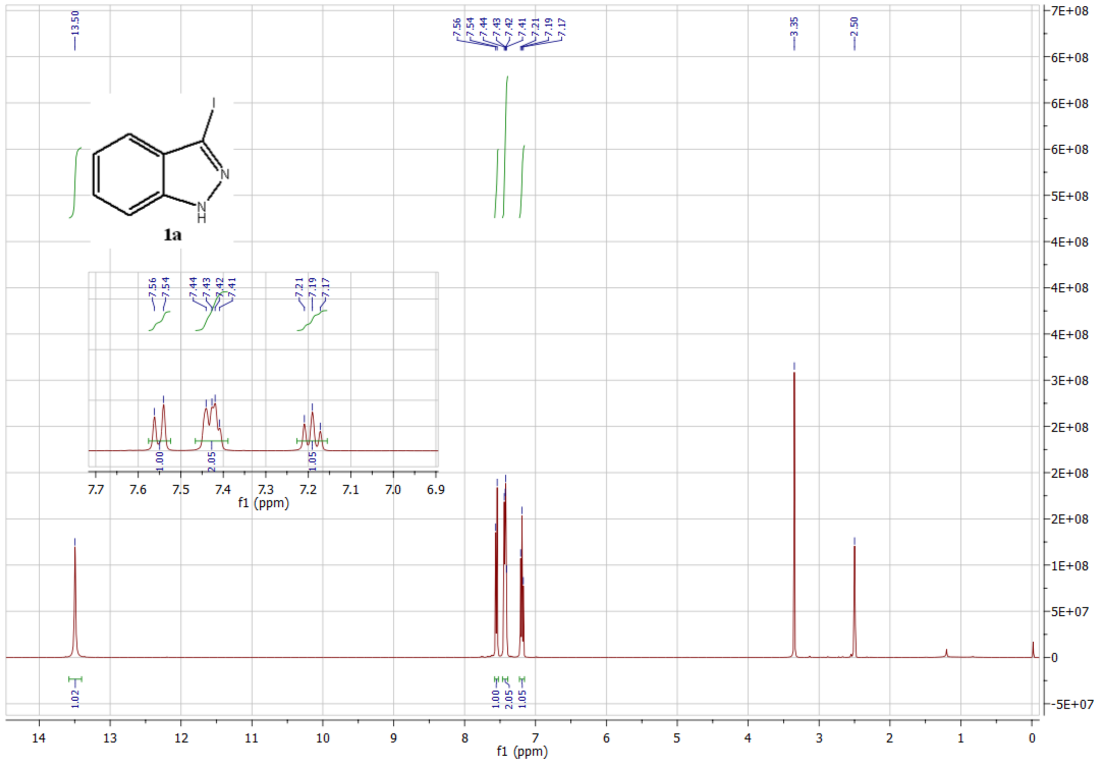

Figure S1. ^{1}H-NMR spectrum of **1a**

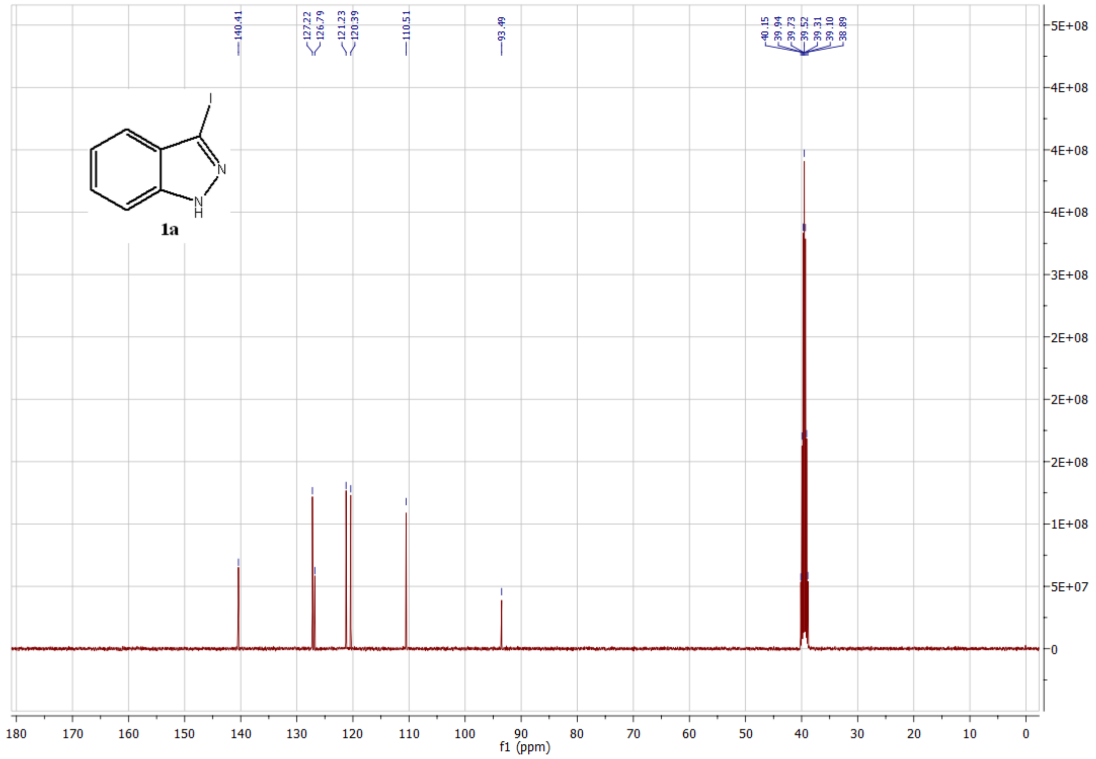

Figure S2. ^{13}C-NMR spectrum of **1a**

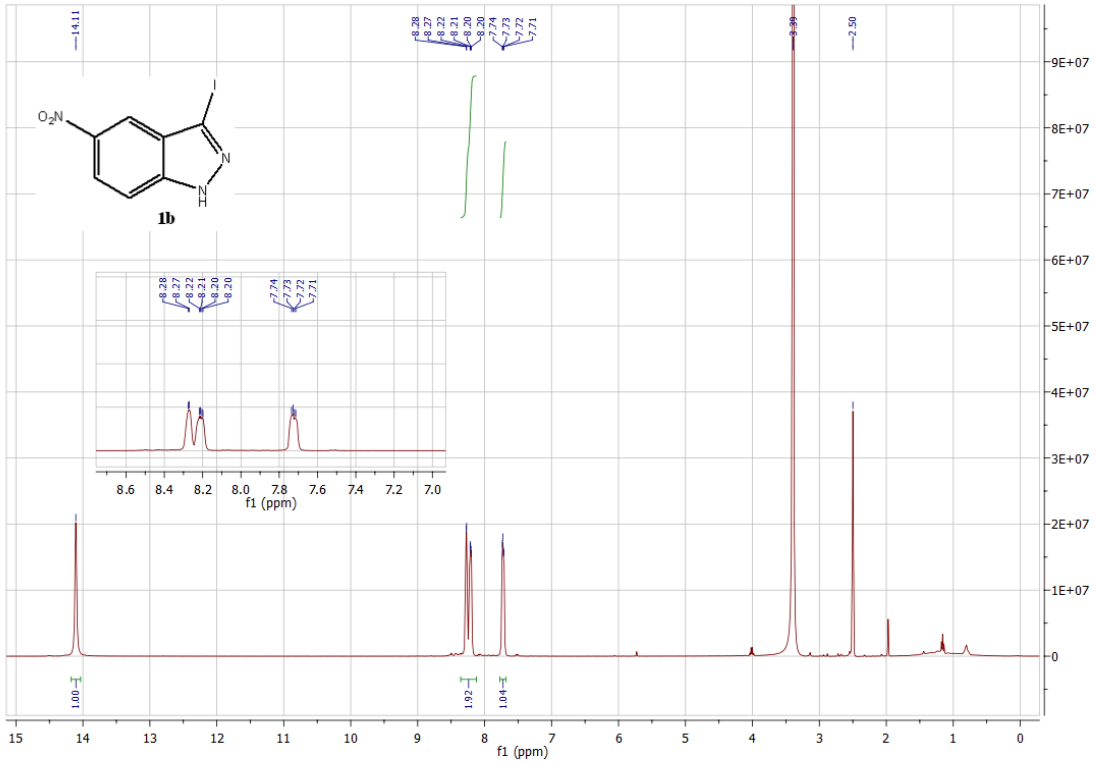

Figure S3. ^{1}H-NMR spectrum of **1b**

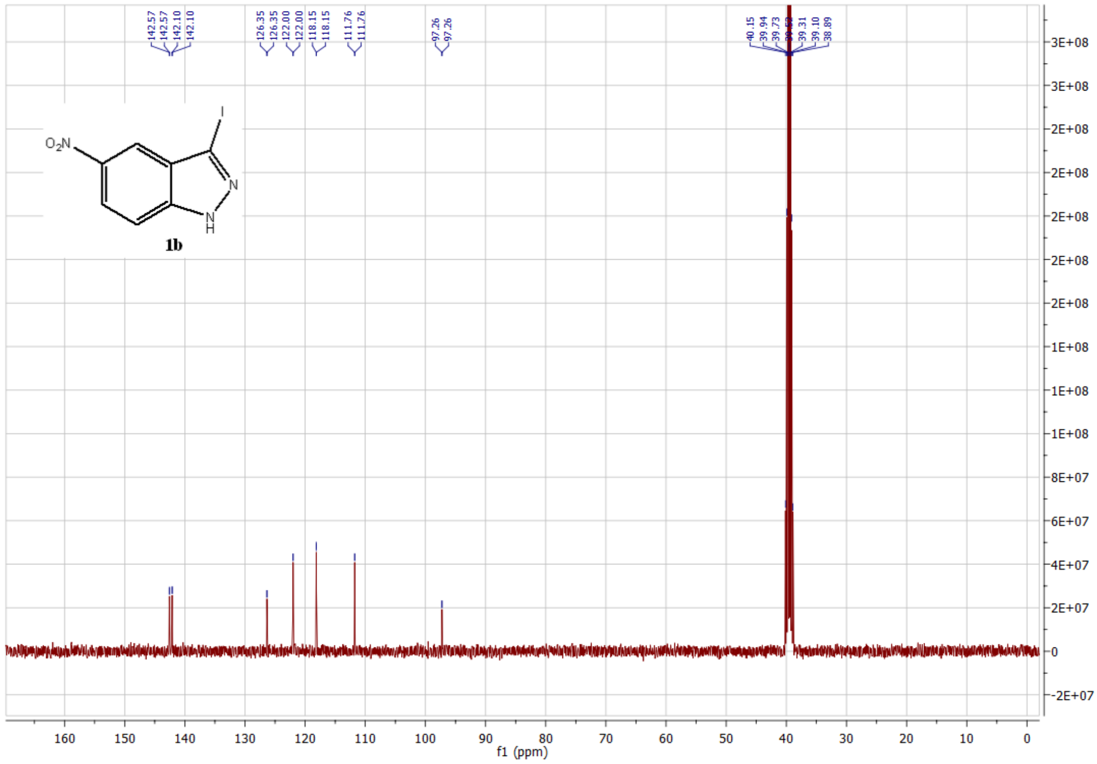

Figure S4. ^{13}C-NMR spectrum of **1b**

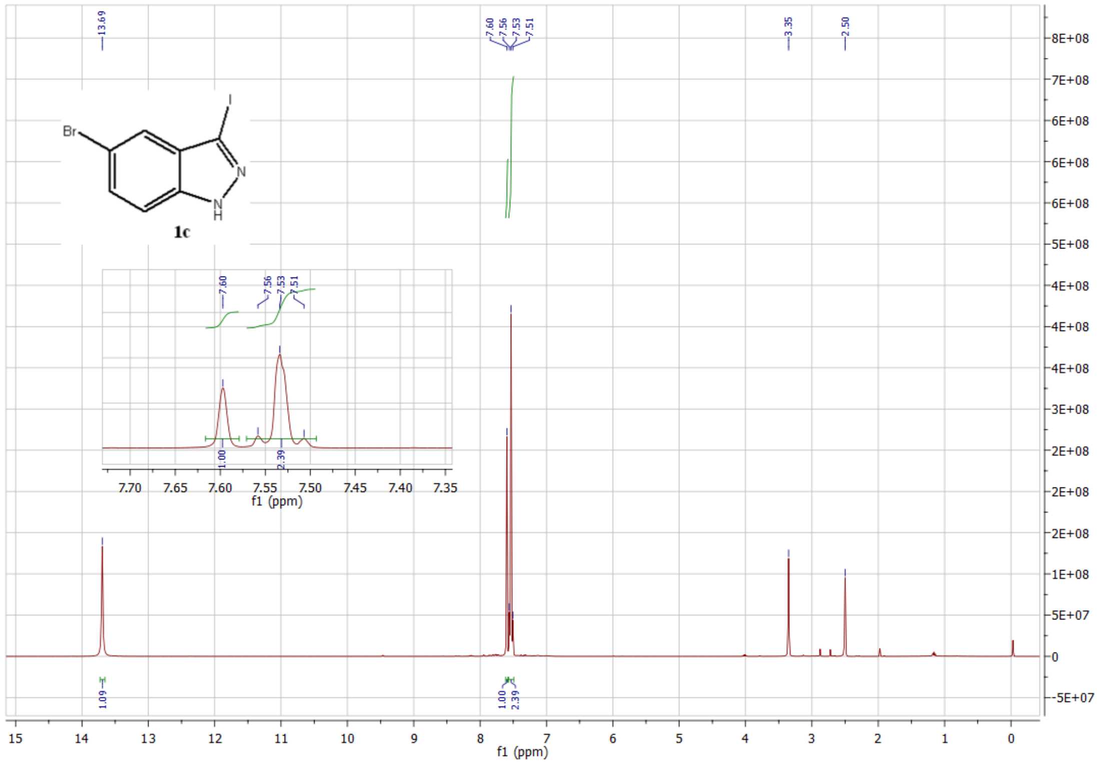

Figure S5. ^{1}H-NMR spectrum of **1c**

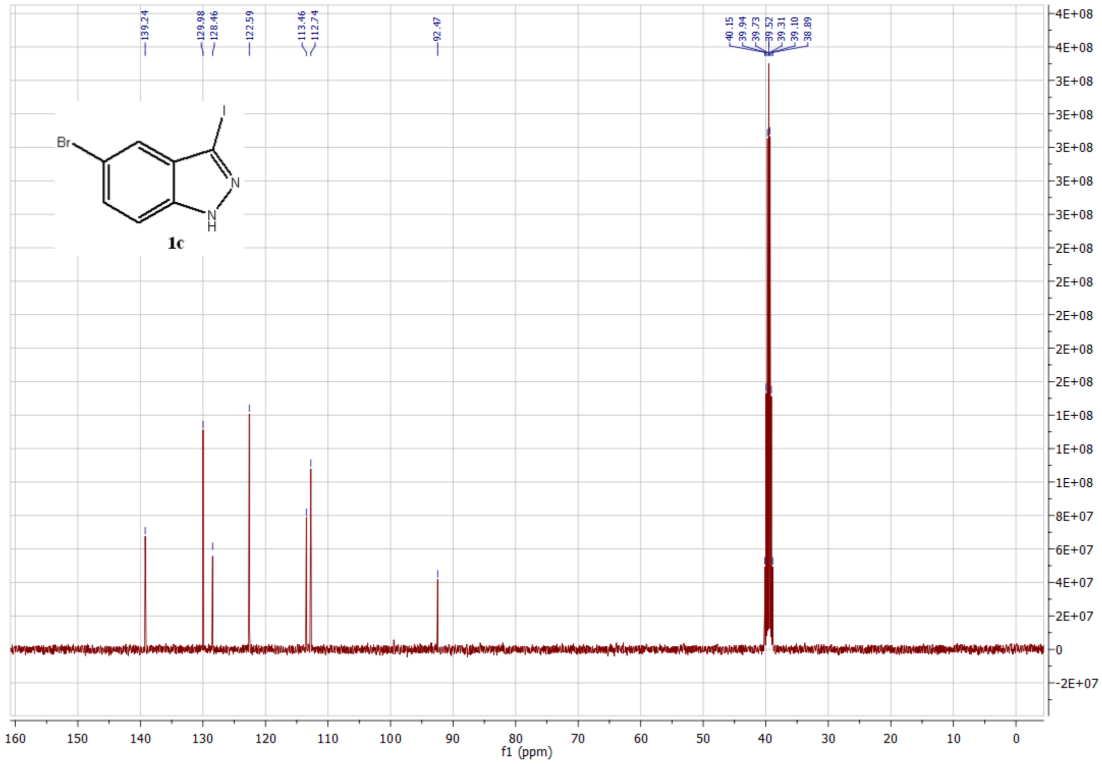

Figure S6. ^{13}C-NMR spectrum of **1c**

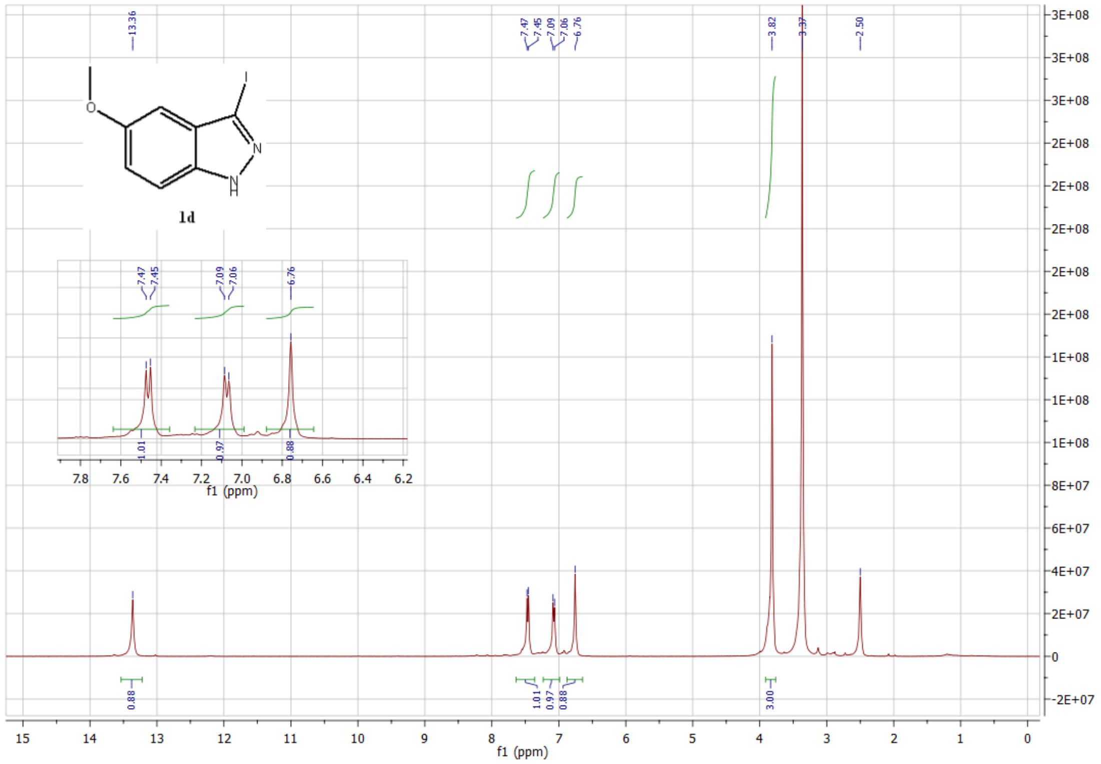

Figure S7. ^{1}H-NMR spectrum of **1d**

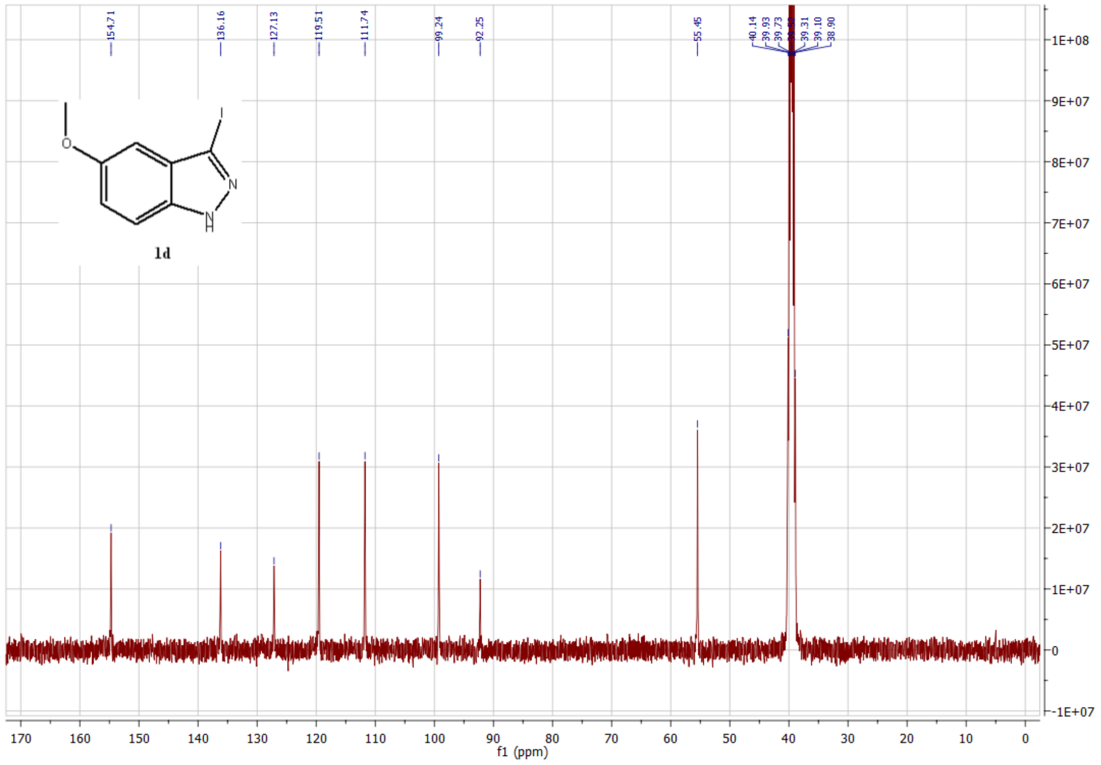

Figure S8. ^{13}C-NMR spectrum of **1d**

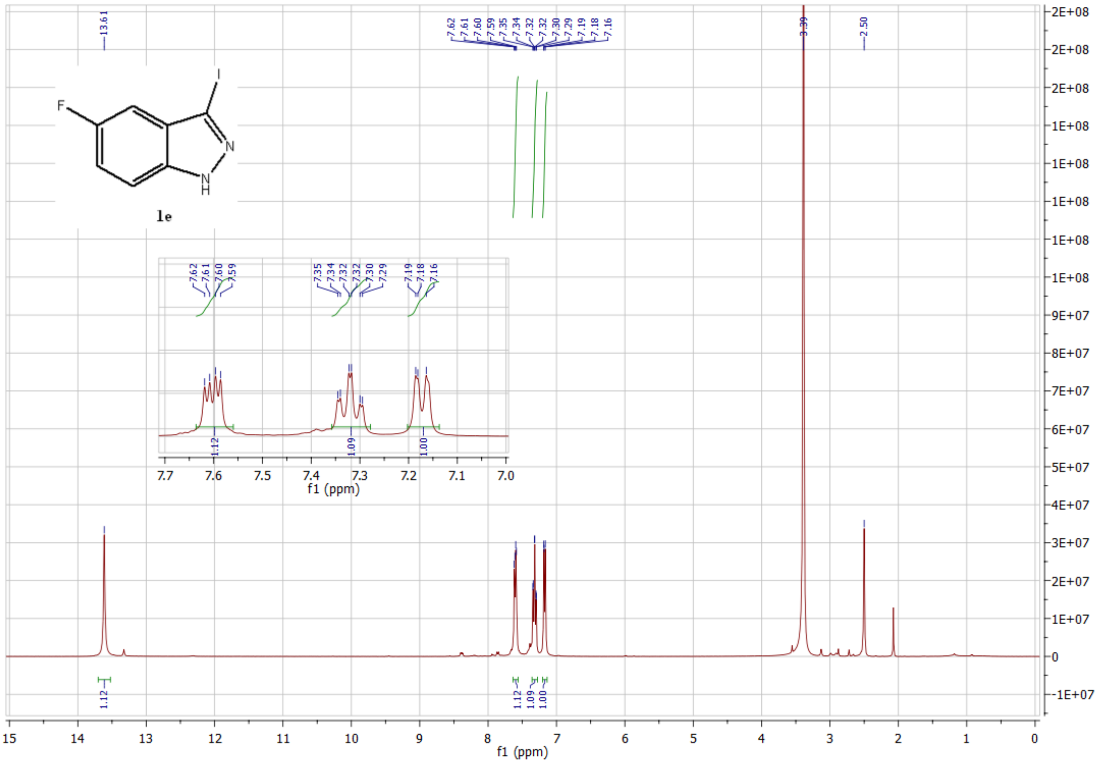

Figure S9. ^{1}H-NMR spectrum of **1e**

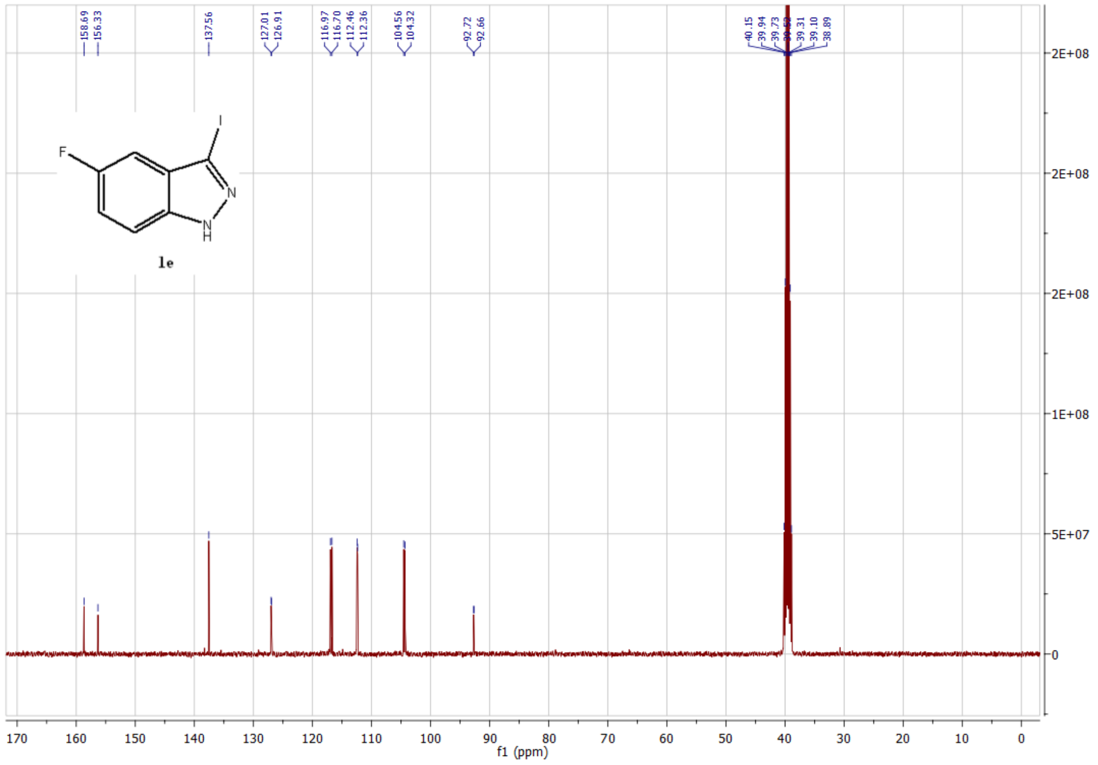

Figure S10. ^{13}C-NMR spectrum of **1e**

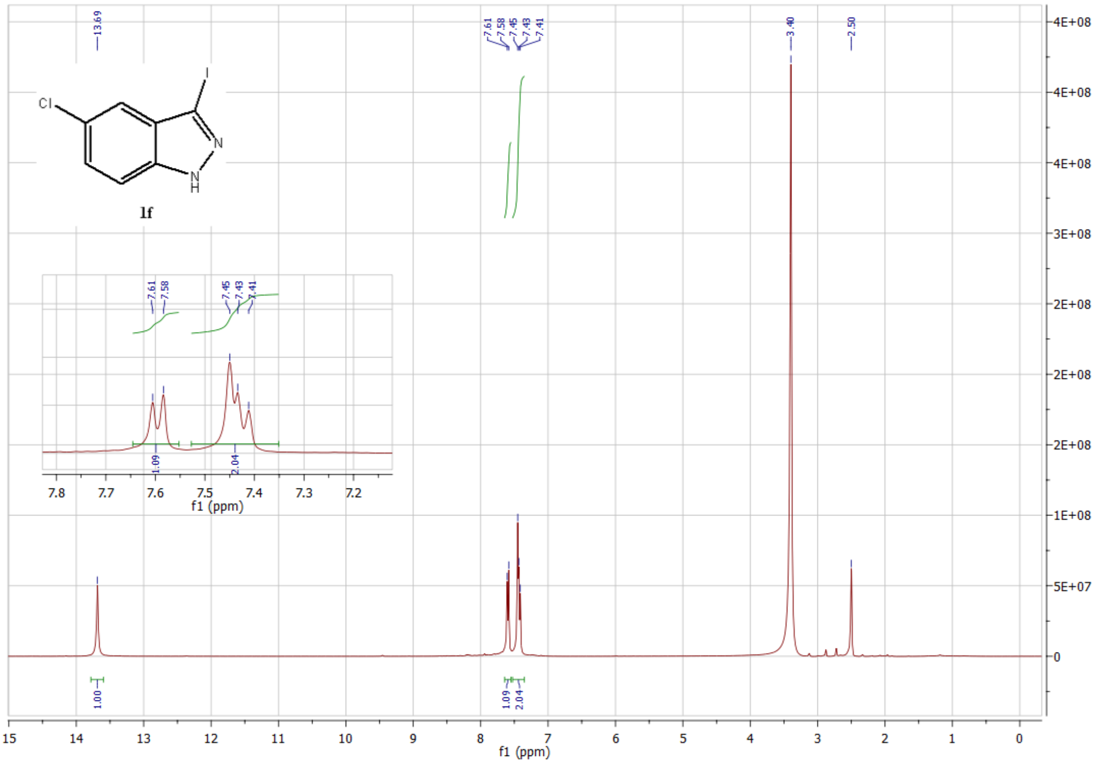

Figure S11. ^{1}H-NMR spectrum of **1f**

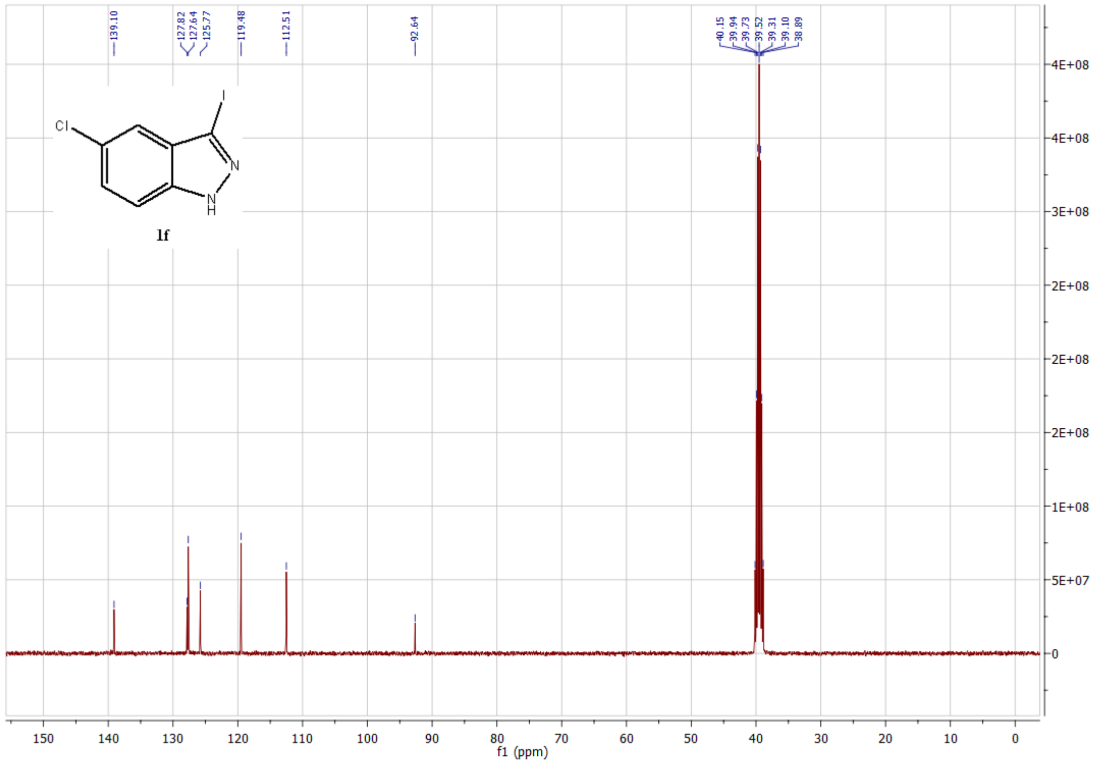

Figure S12. ^{13}C-NMR spectrum of **1f**

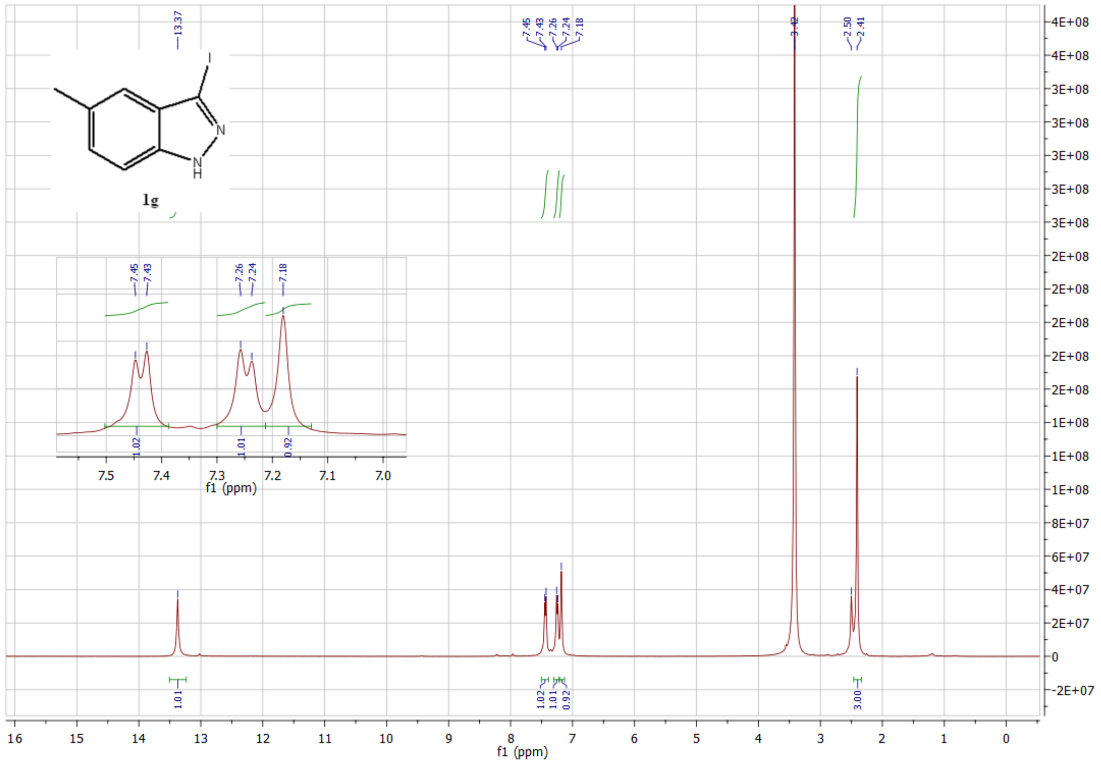

Figure S13. ^{1}H-NMR spectrum of **1g**

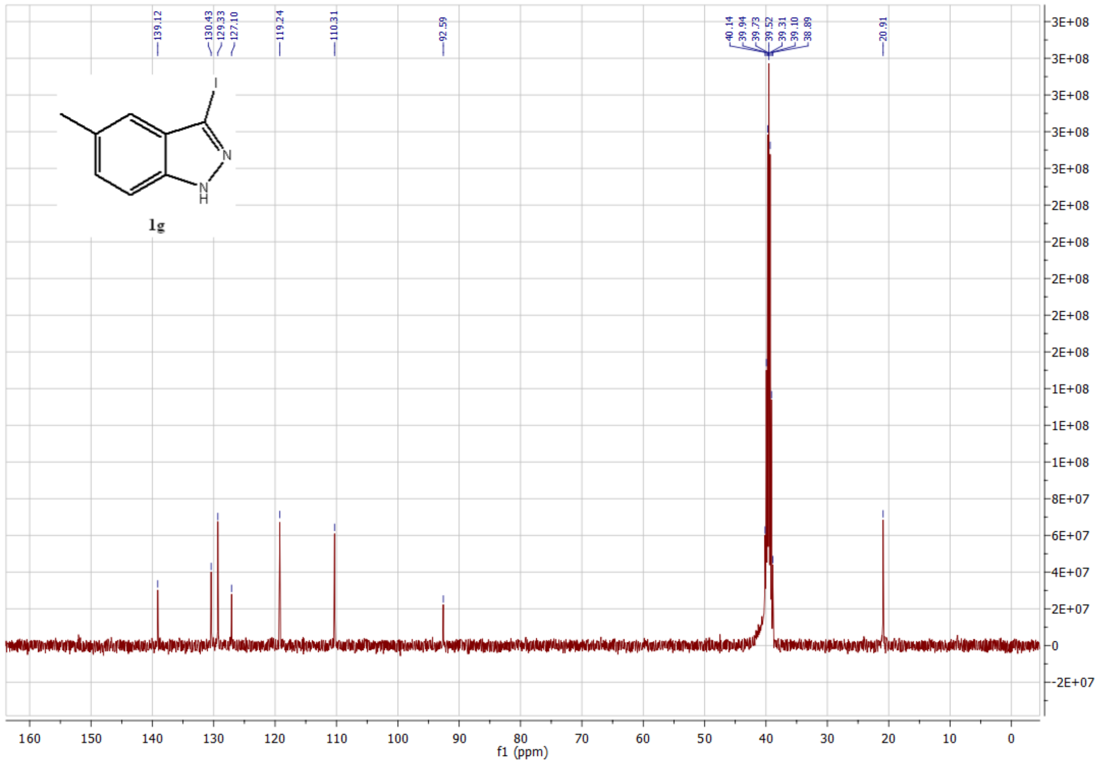

Figure S14. ^{13}C-NMR spectrum of **1g**

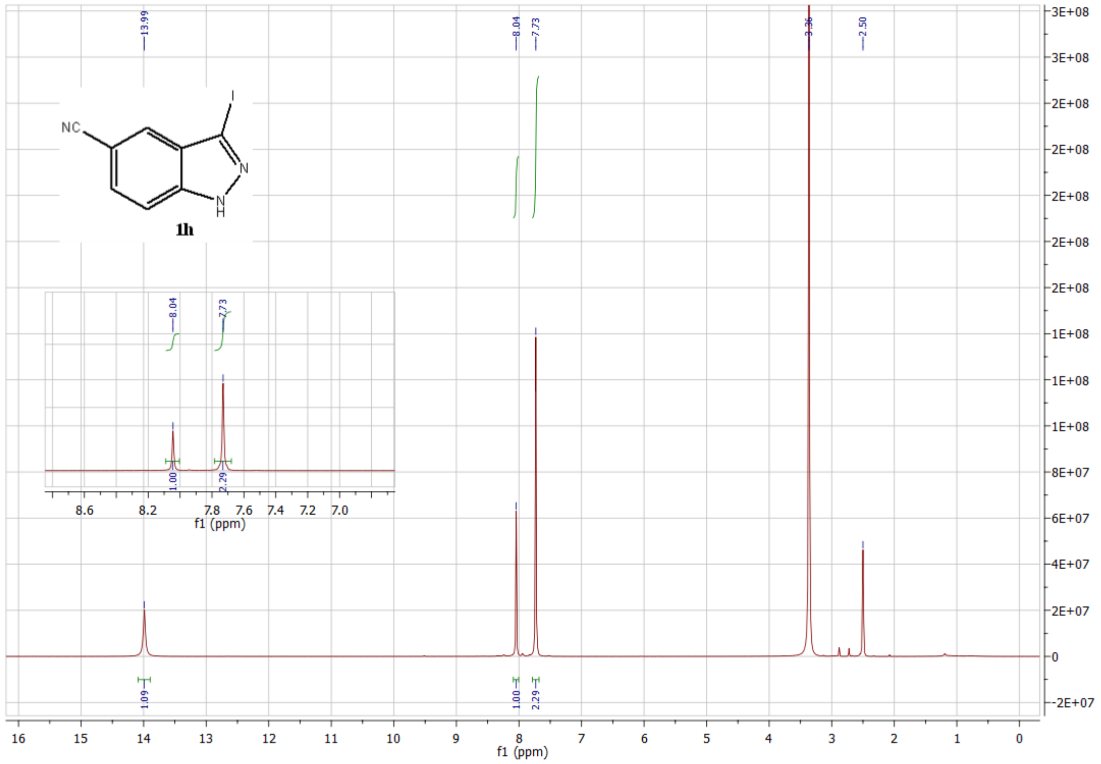

Figure S15. ^{1}H-NMR spectrum of **1h**

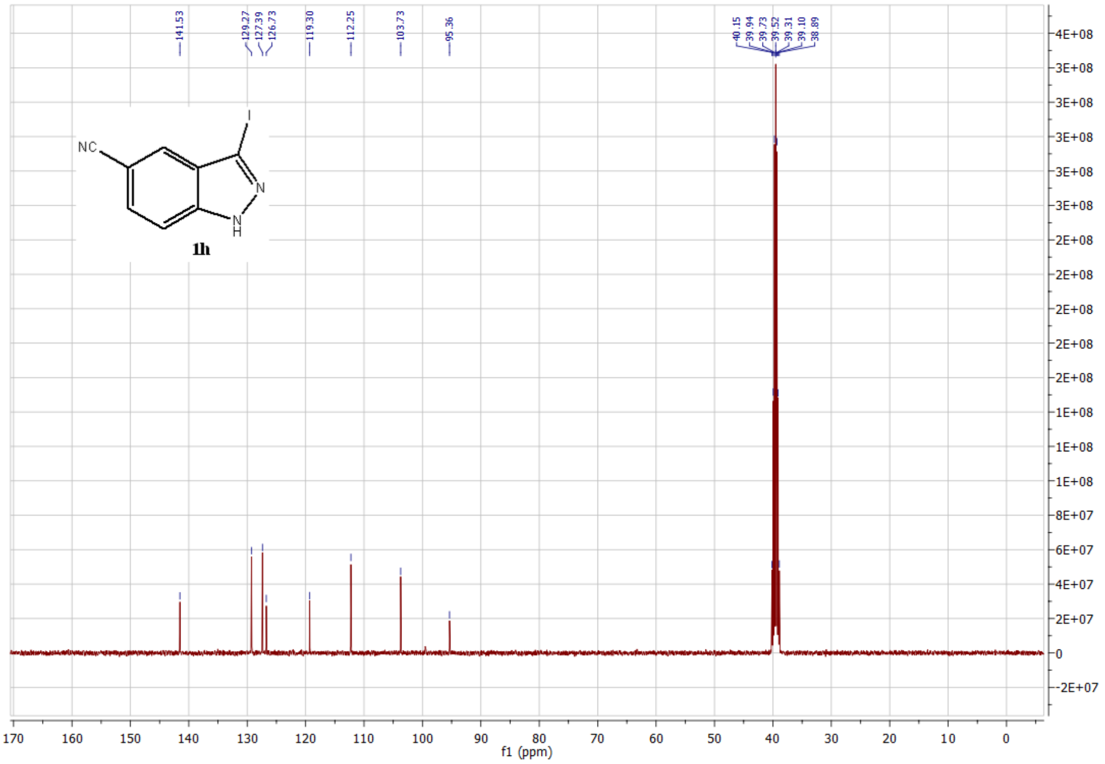

Figure S16. ^{13}C-NMR spectrum of **1h**

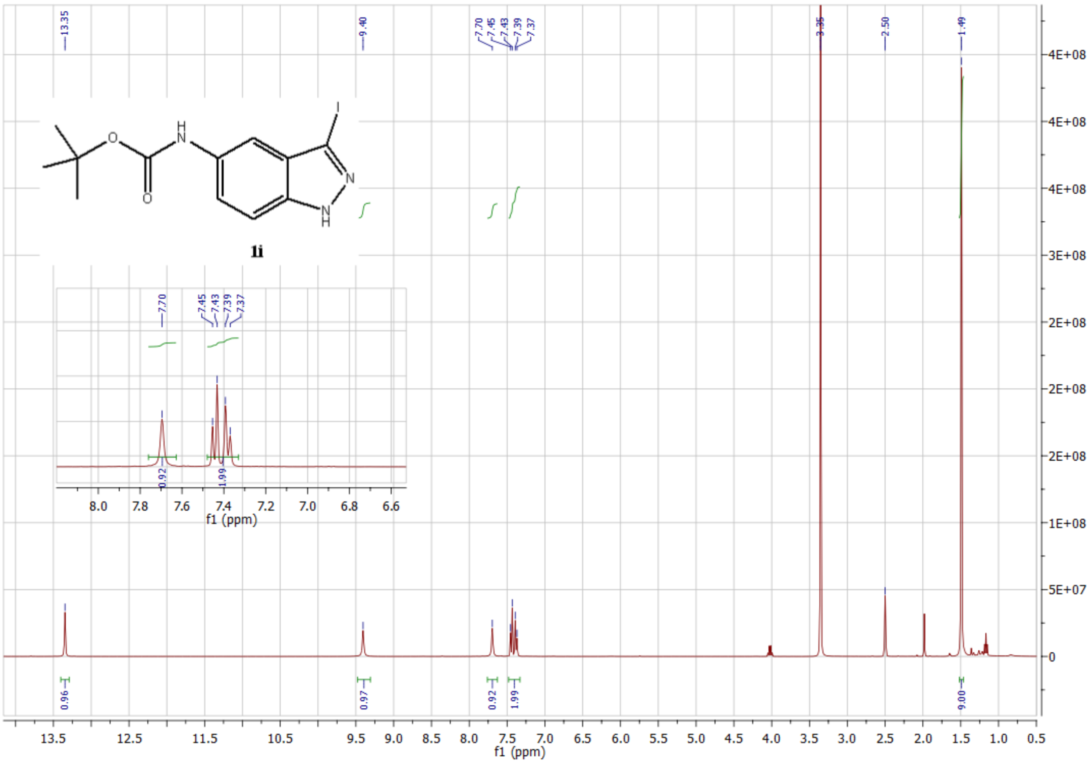

Figure S17. ^{1}H-NMR spectrum of **1i**

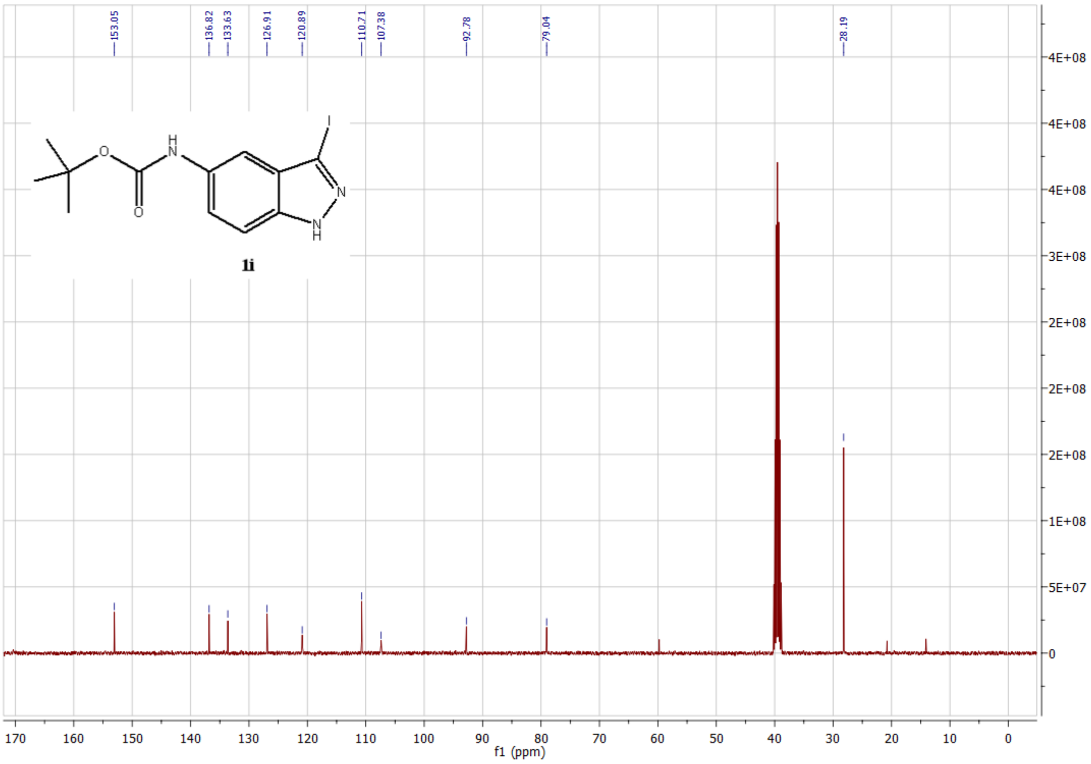

Figure S18. ^{13}C-NMR spectrum of **1i**

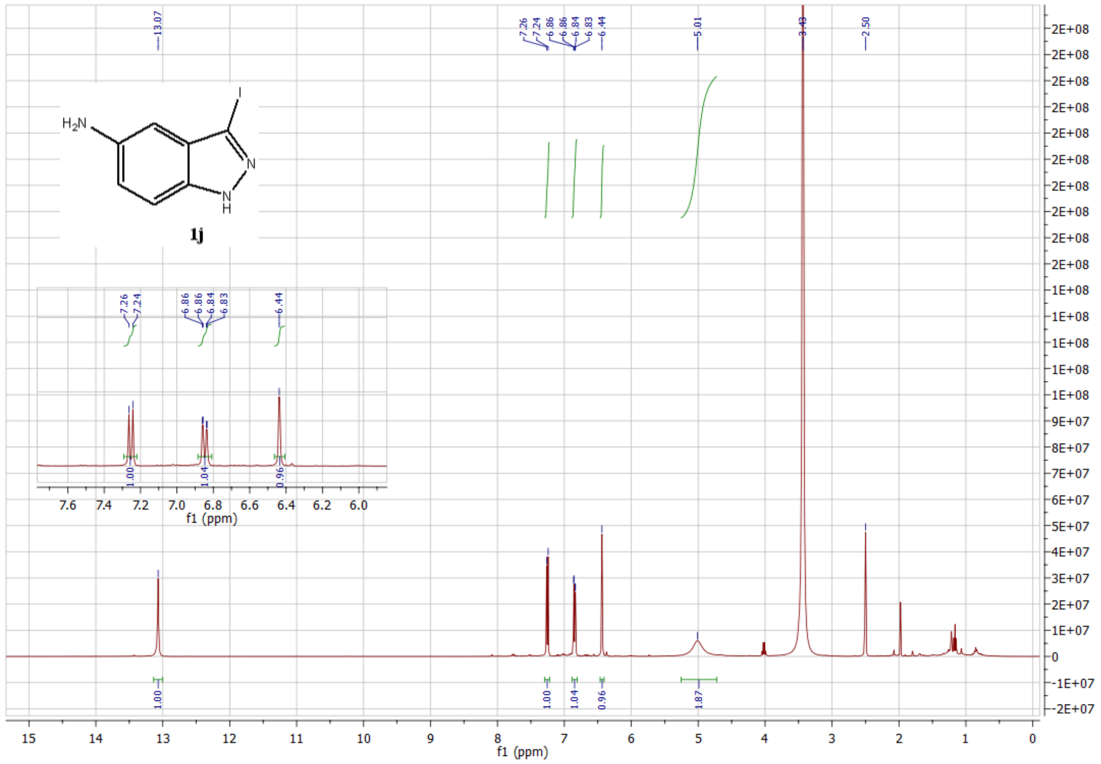

Figure S19. ^{1}H-NMR spectrum of **1j**

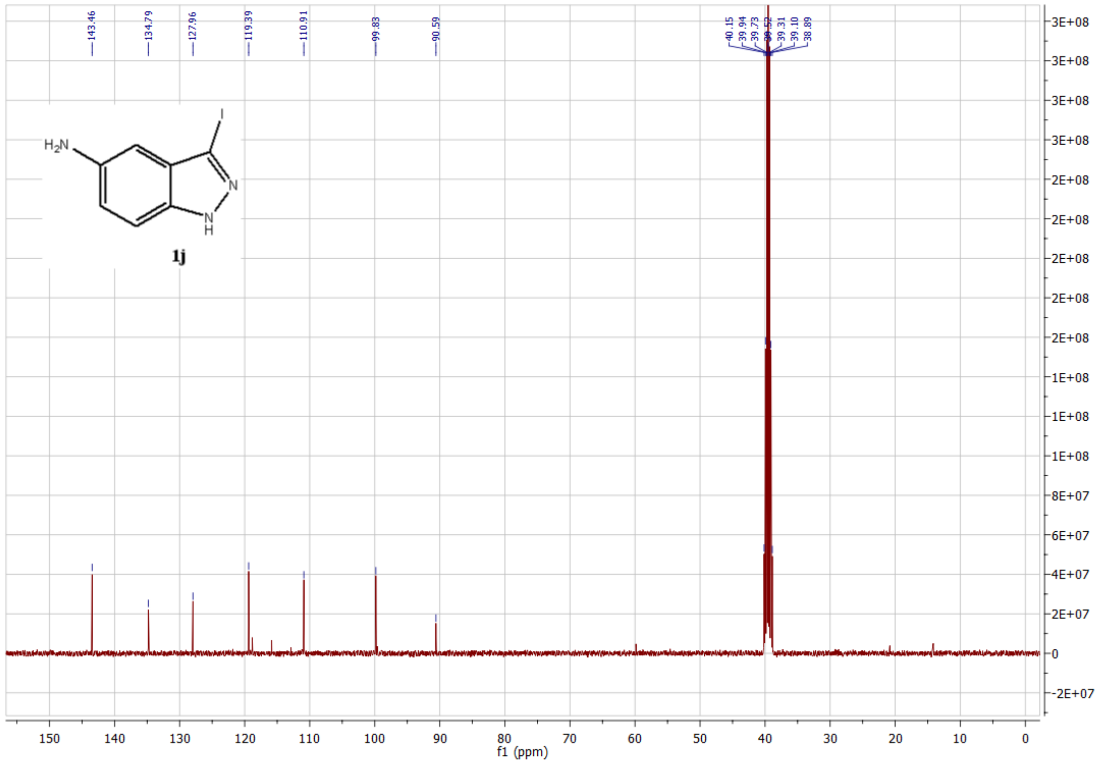

Figure S20. ^{13}C-NMR spectrum of **1j**

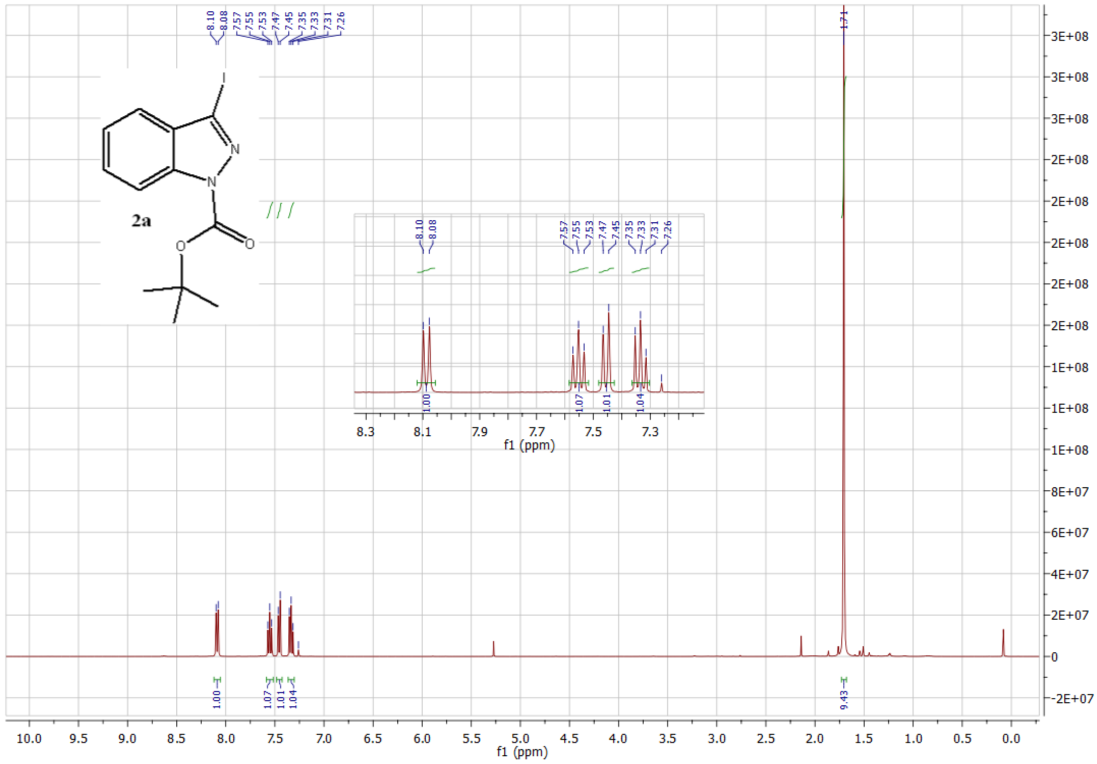

Figure S21. ^{1}H-NMR spectrum of **2a**

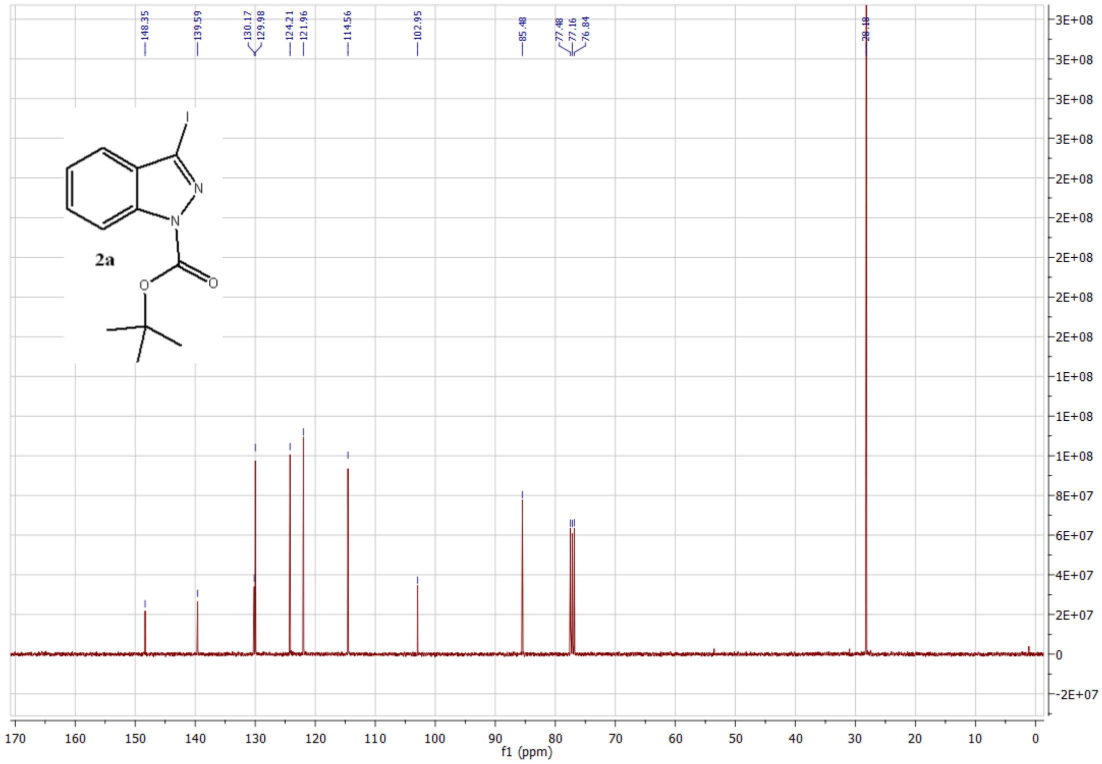

Figure S22. ^{13}C-NMR spectrum of **2a**

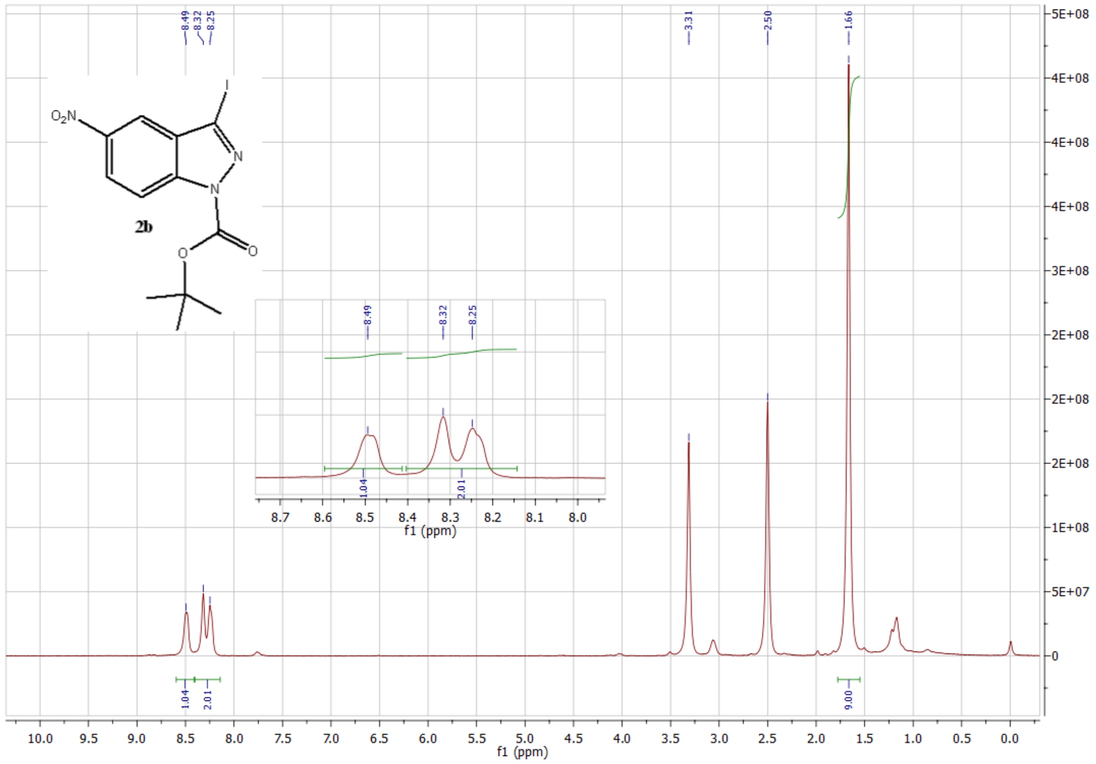

Figure S23. ^{1}H-NMR spectrum of **2b**

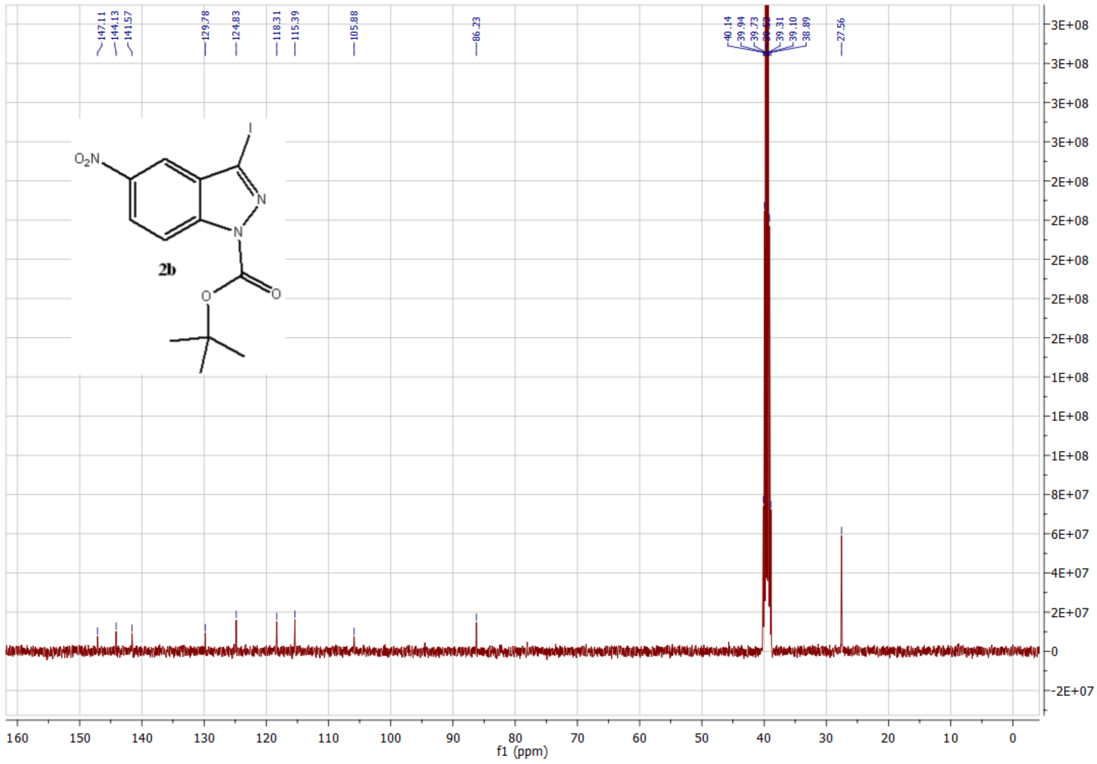

Figure S24. ^{13}C-NMR spectrum of **2b**

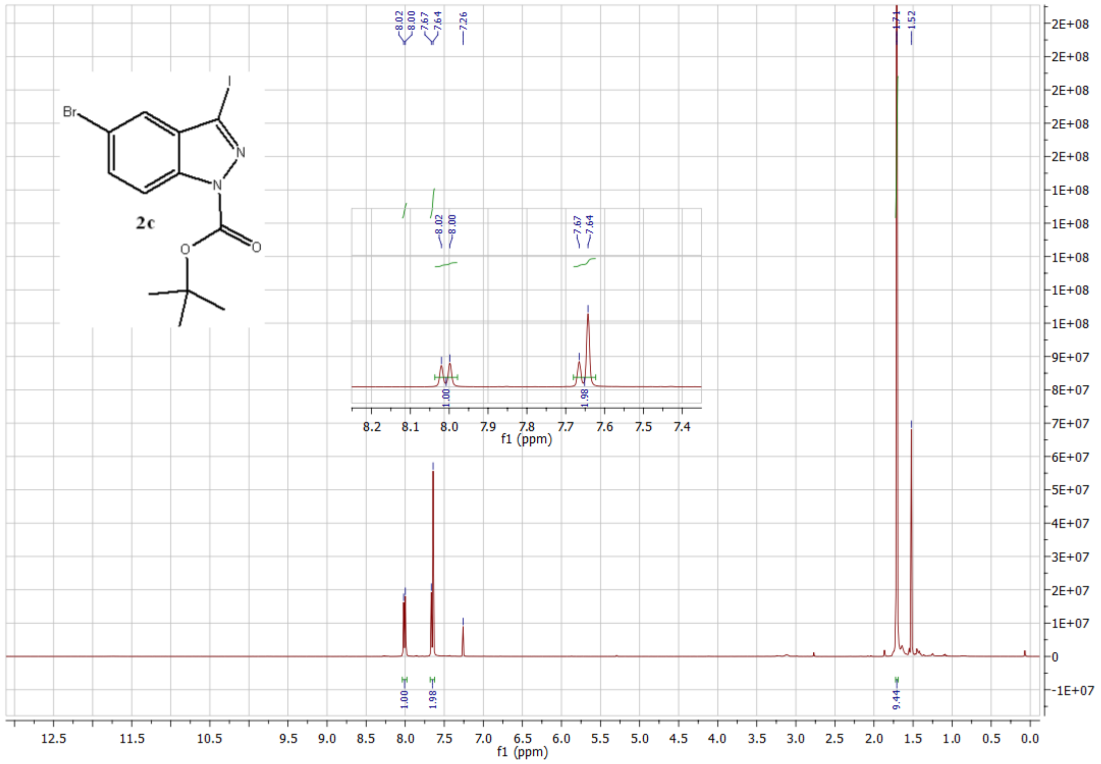

Figure S25. ^{1}H-NMR spectrum of **2c**

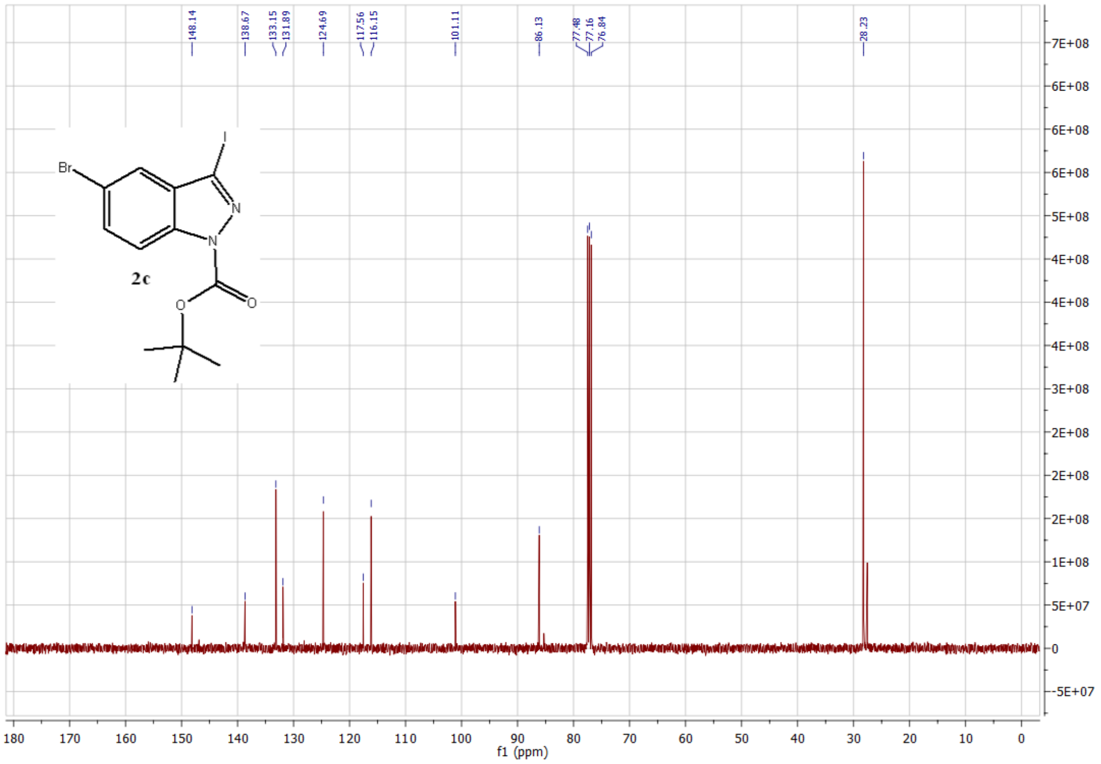

Figure S26. ^{13}C-NMR spectrum of **2c**

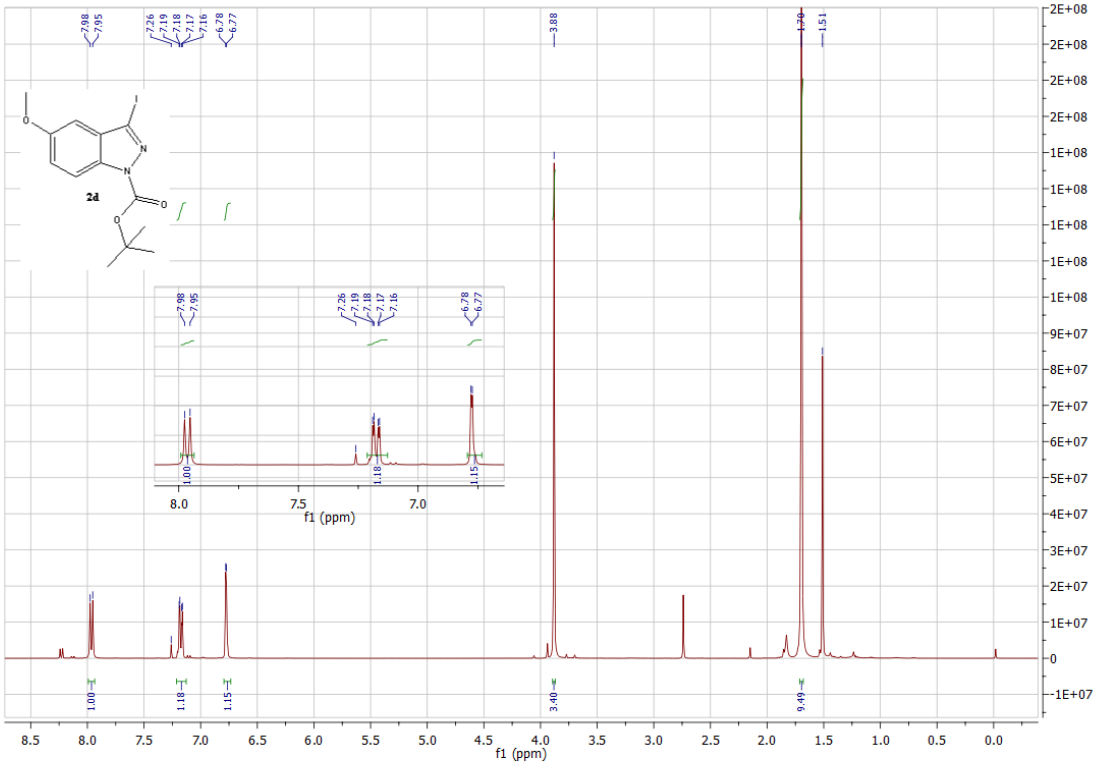

Figure S27. ^{1}H-NMR spectrum of **2d**

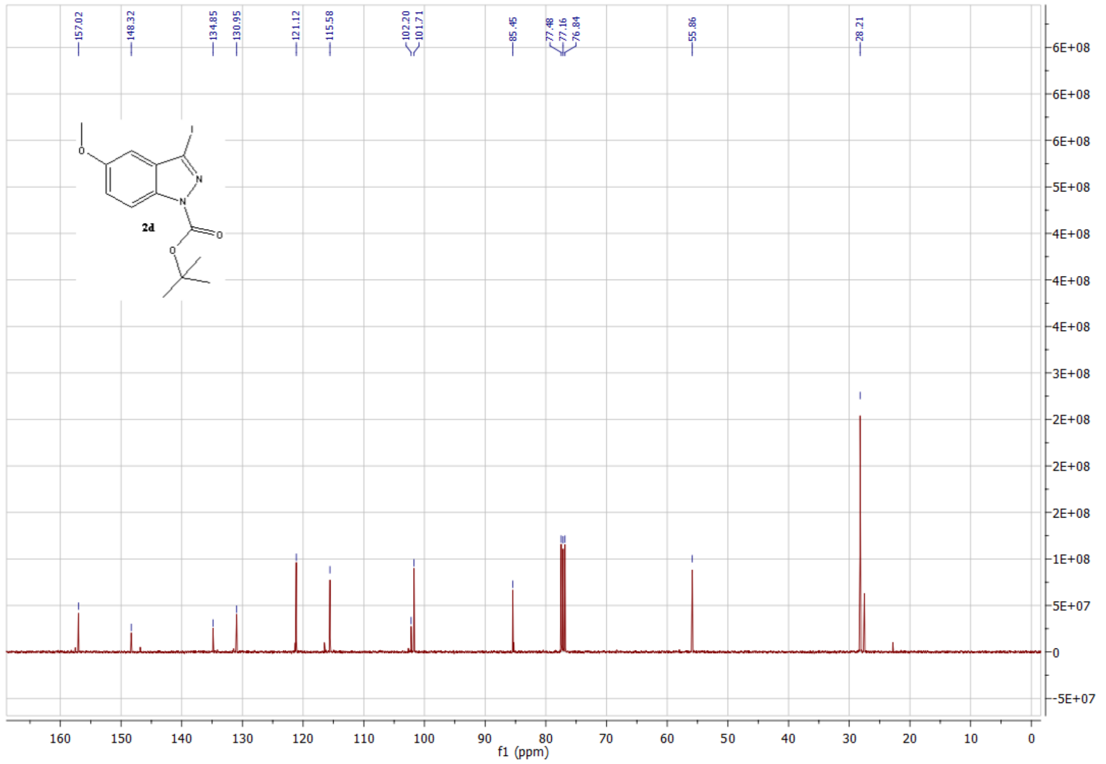

Figure S28. ^{13}C-NMR spectrum of **2d**

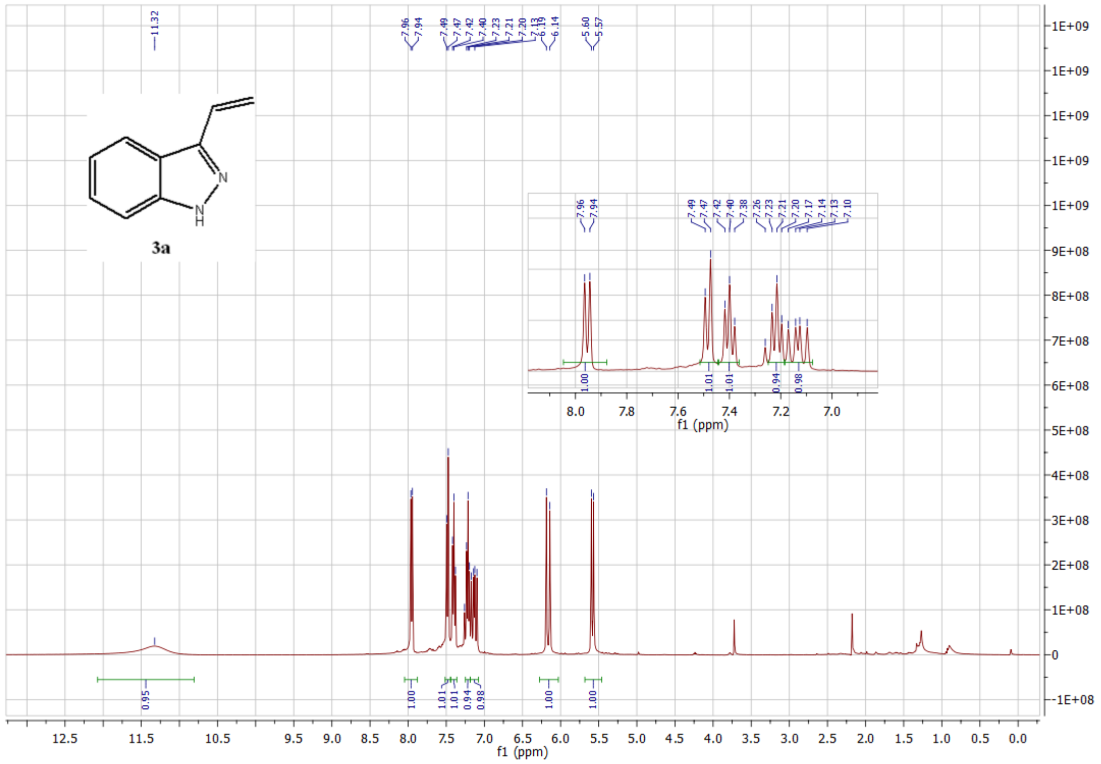

Figure S29. ^{1}H-NMR spectrum of **3a**

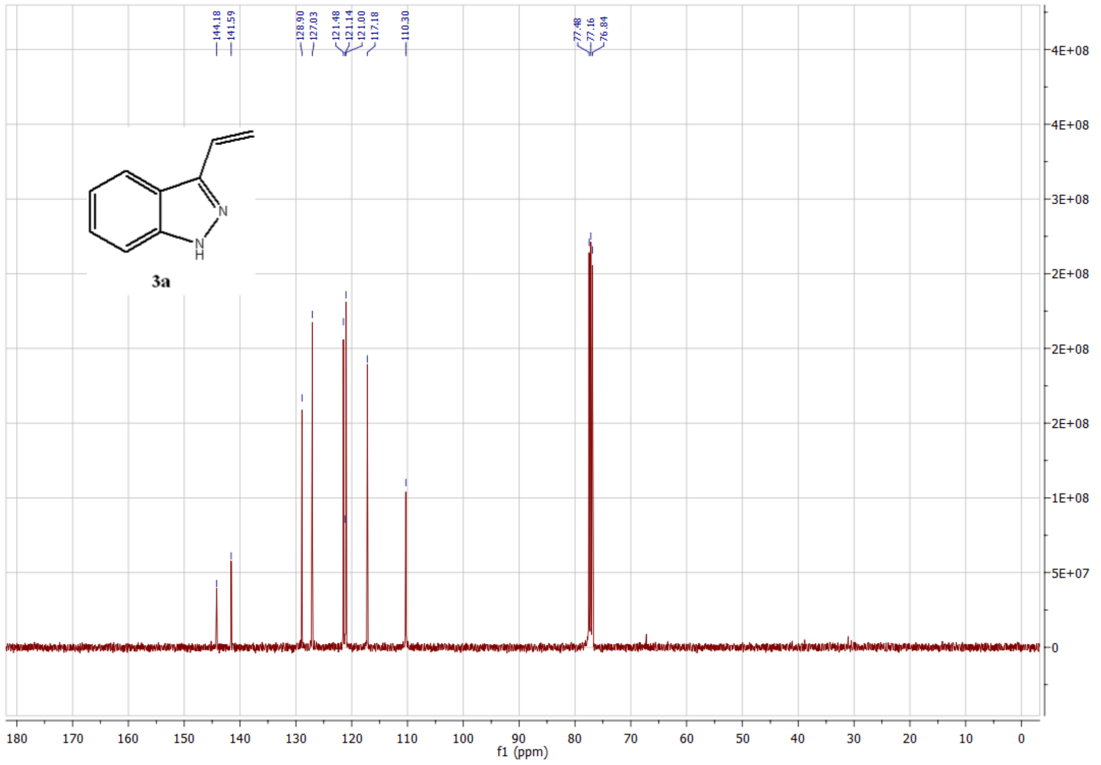

Figure S30. ^{13}C-NMR spectrum of **3a**

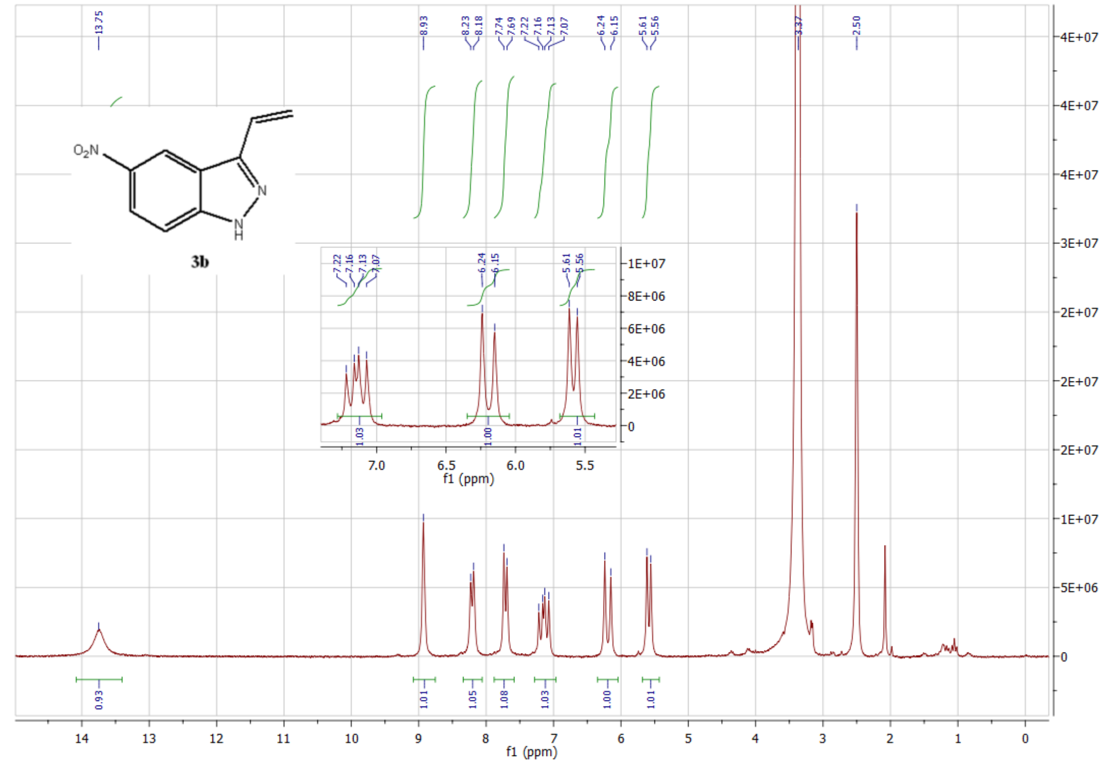

Figure S31. ^{1}H-NMR spectrum of **3b**

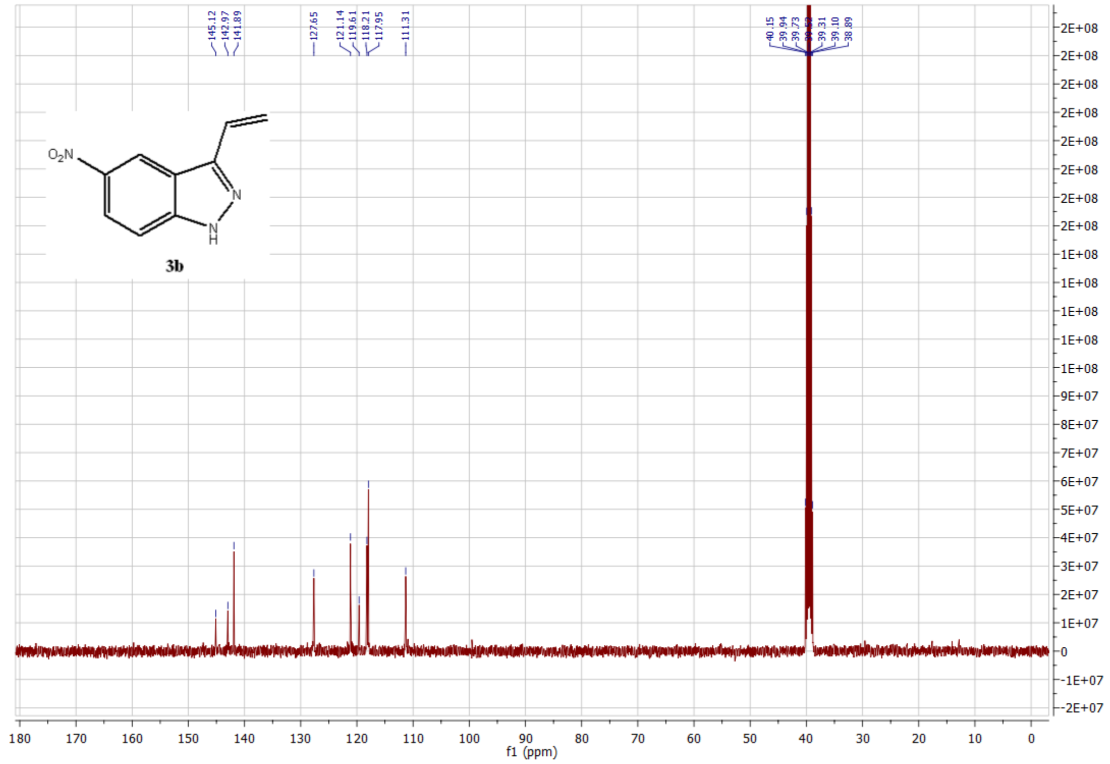

Figure S32. ^{13}C-NMR spectrum of **3b**

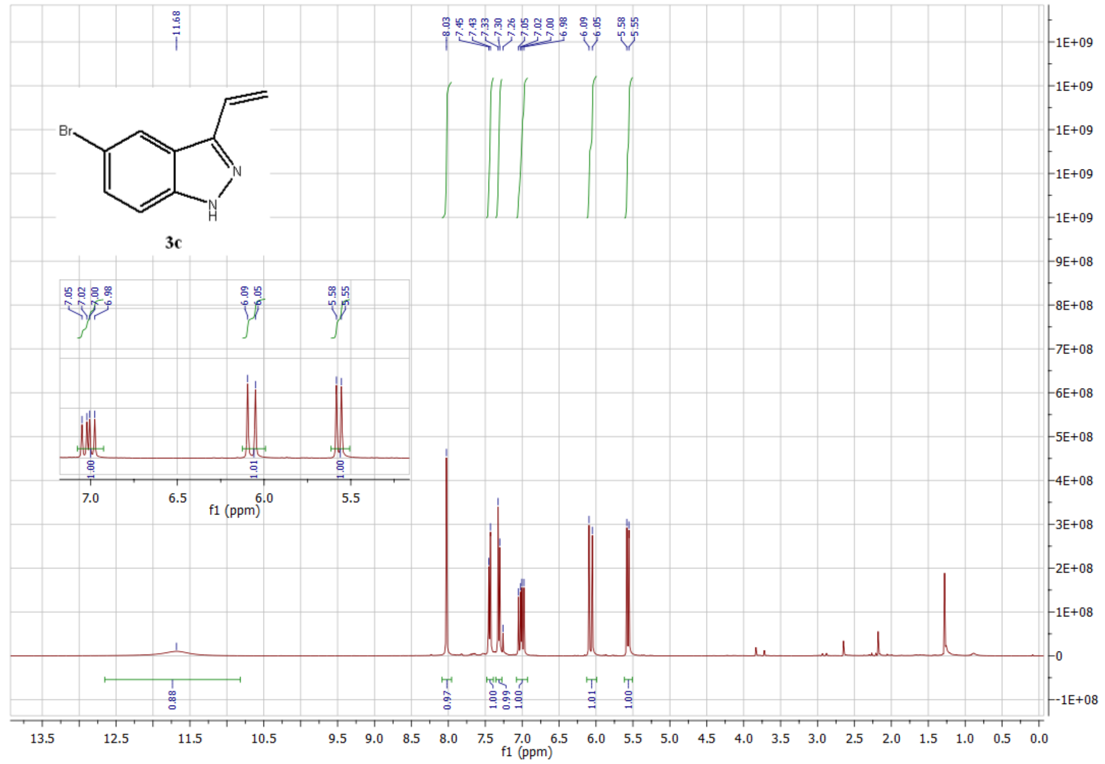

Figure S33. ^{1}H-NMR spectrum of **3c**

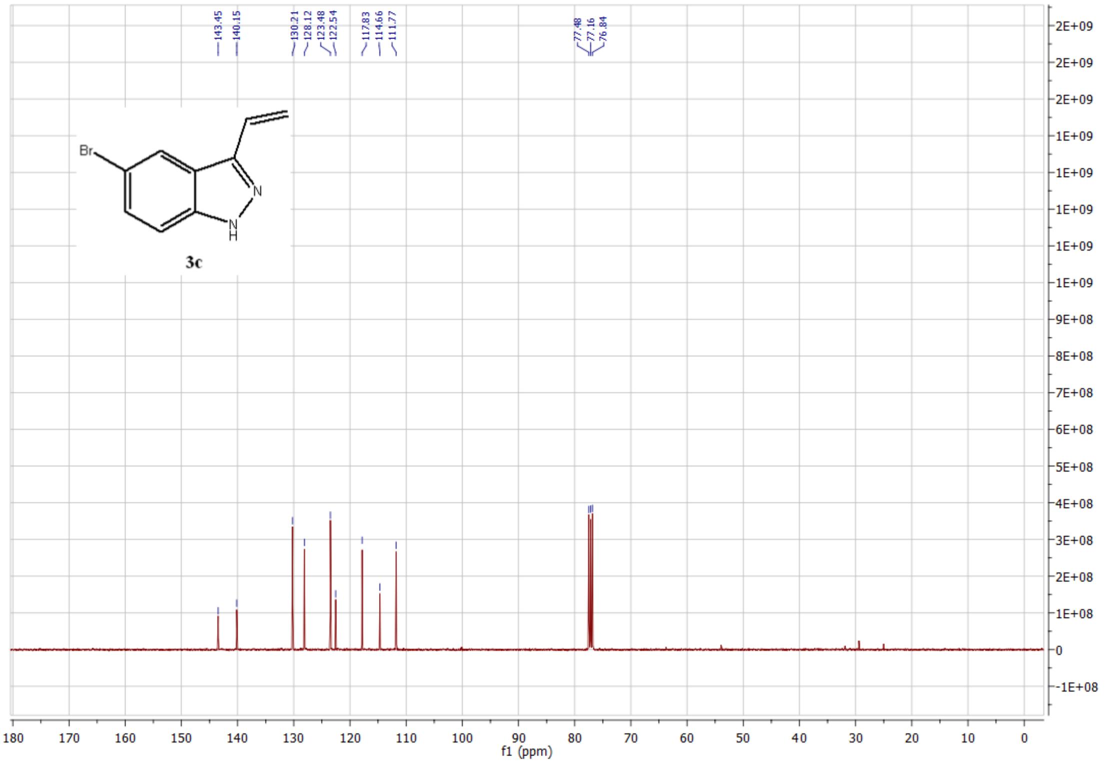

Figure S34. ^{13}C-NMR spectrum of **3c**

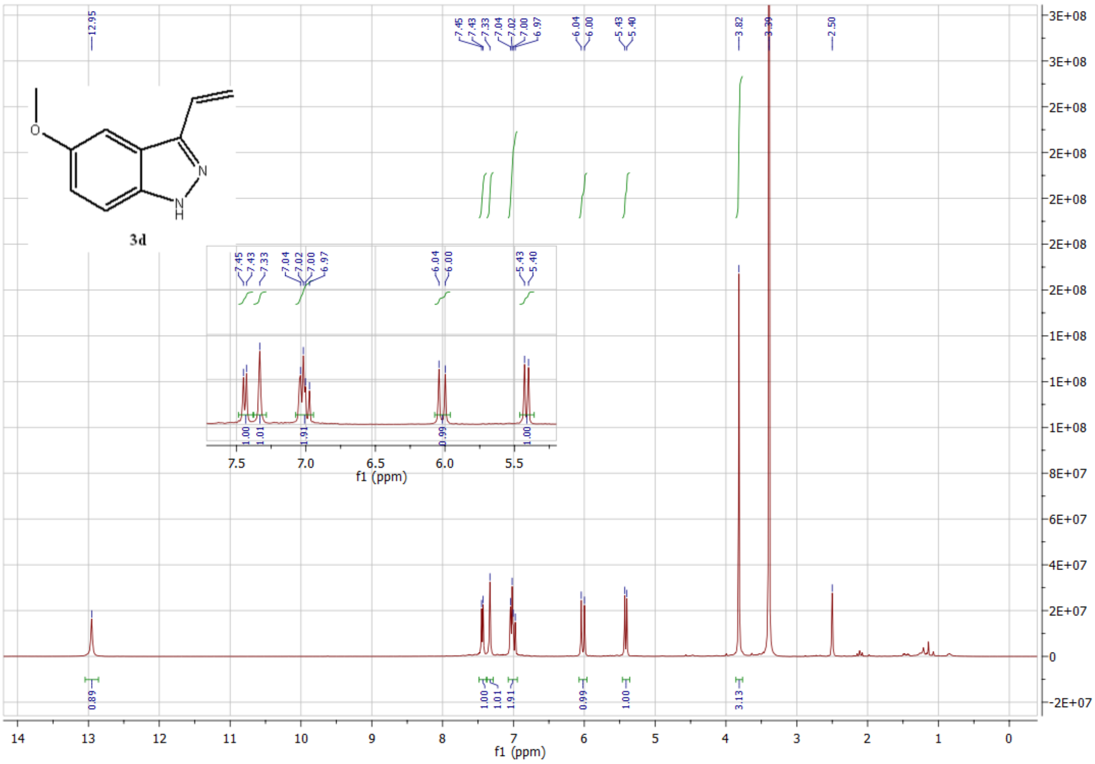

Figure S35. ^{1}H-NMR spectrum of **3d**

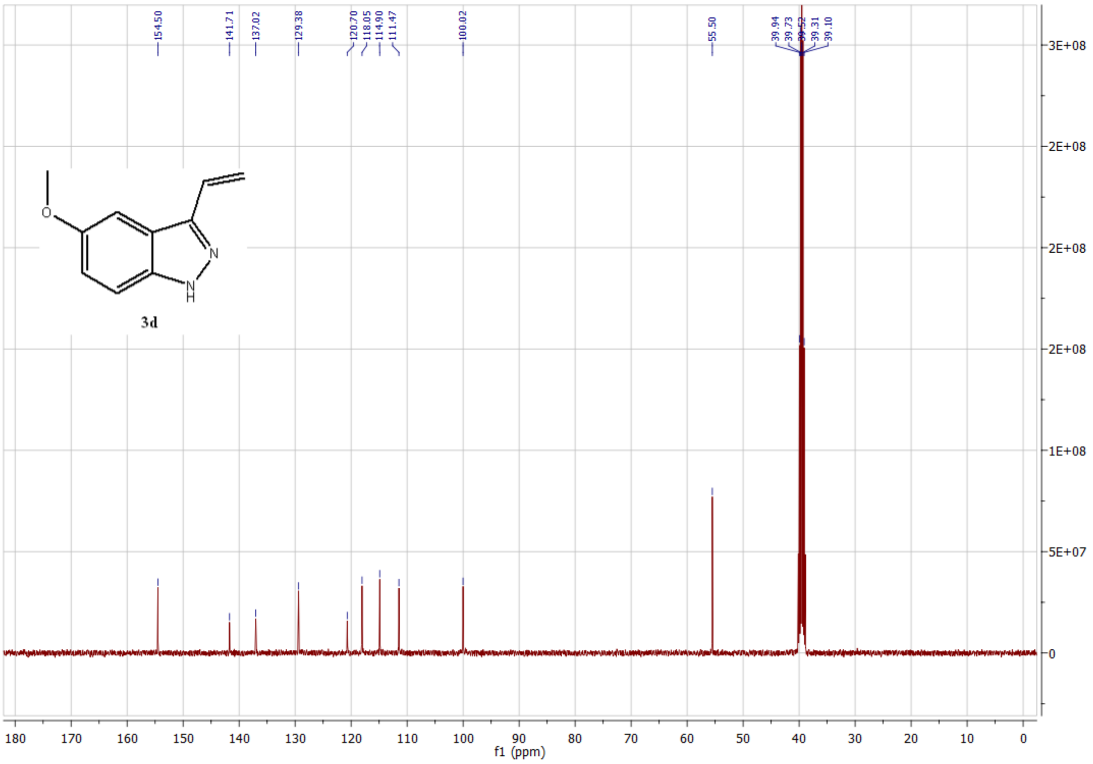

Figure S36. ^{13}C-NMR spectrum of **3d**

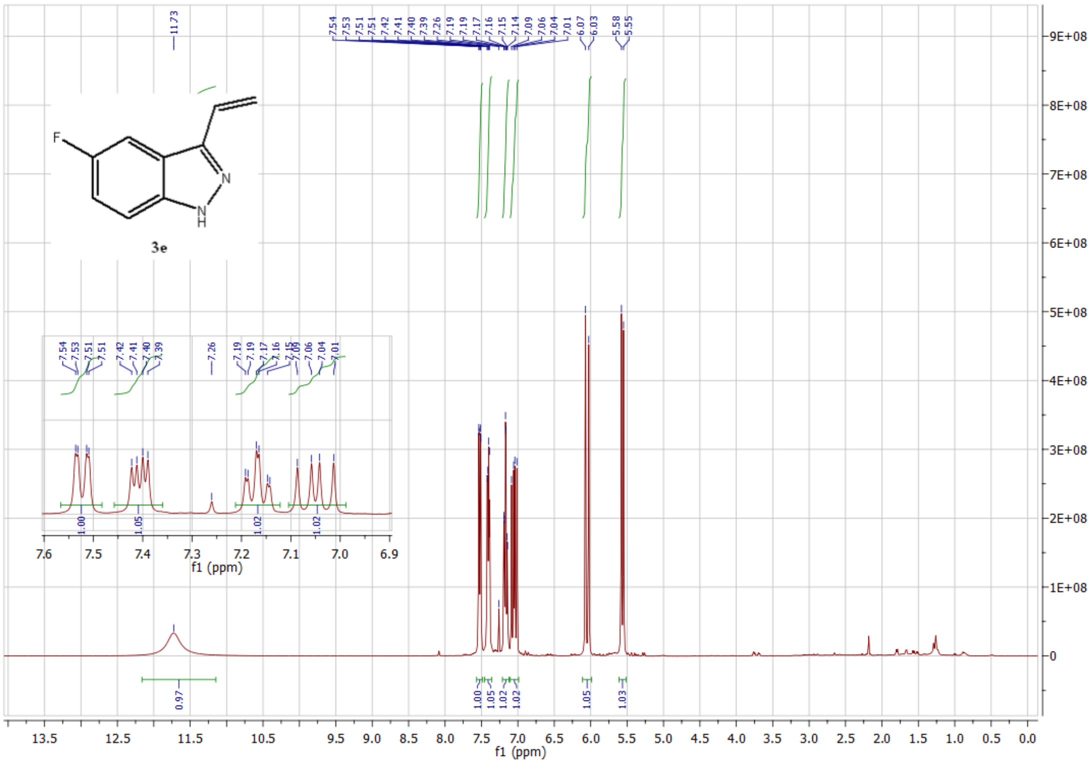

Figure S37. ^{1}H-NMR spectrum of **3e**

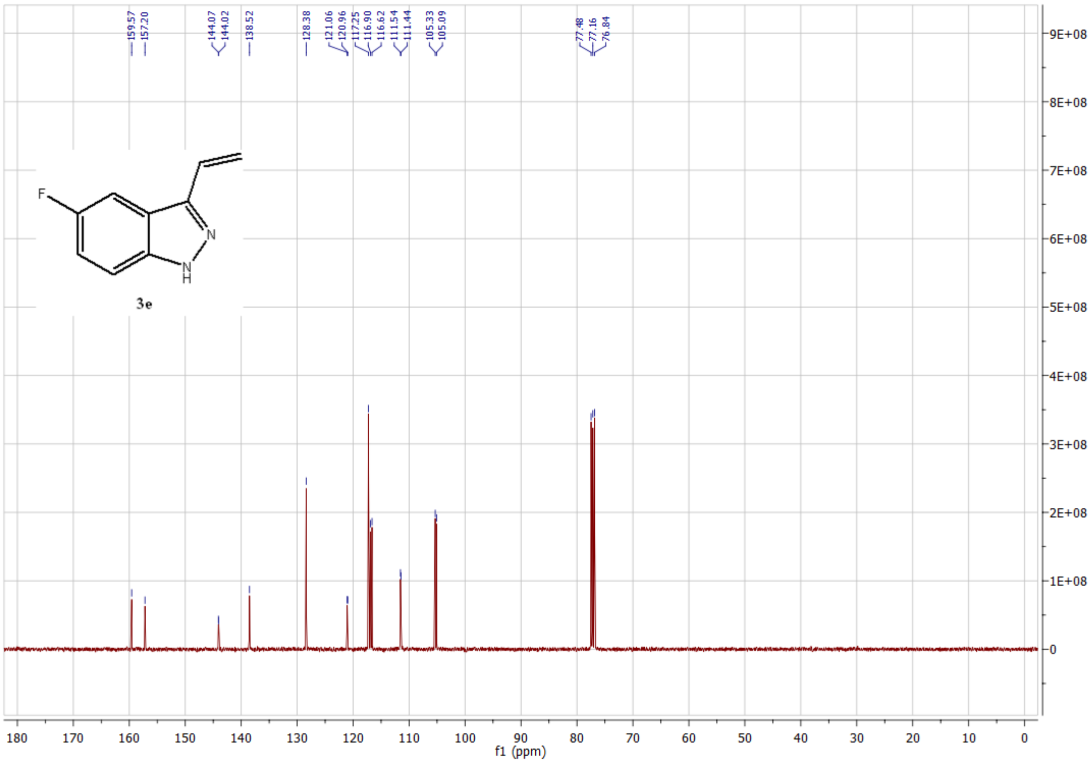

Figure S38. ^{13}C-NMR spectrum of **3e**

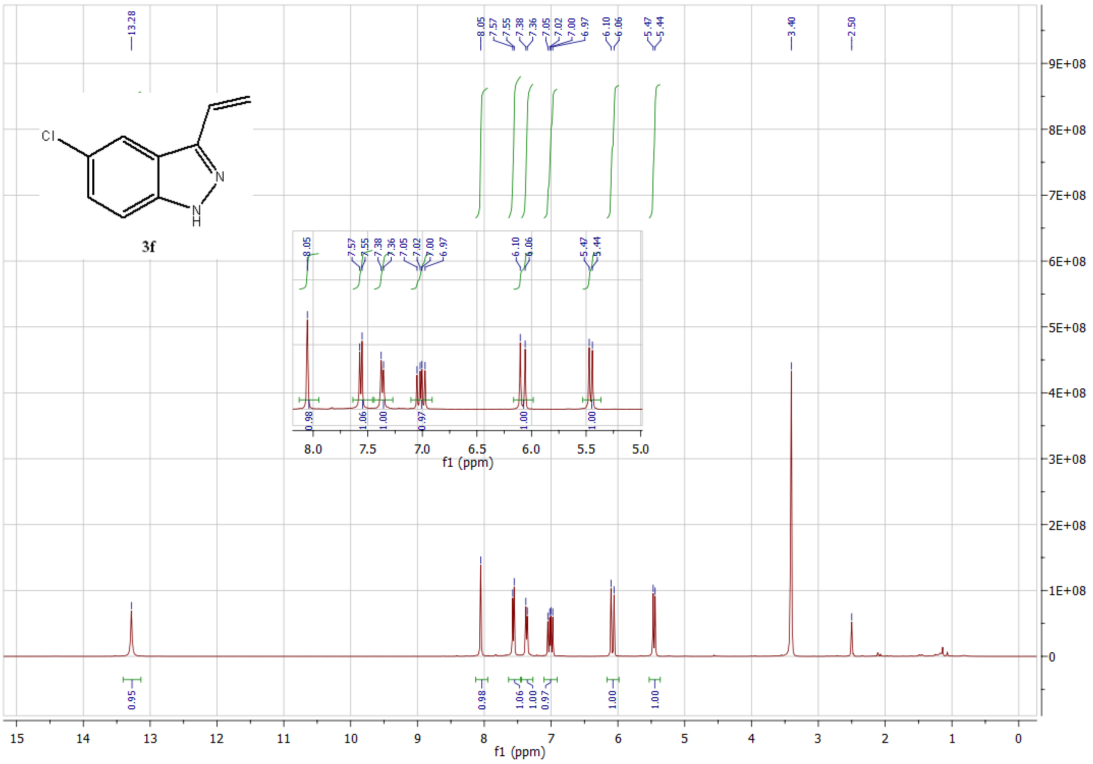

Figure S39. ^{1}H-NMR spectrum of **3f**

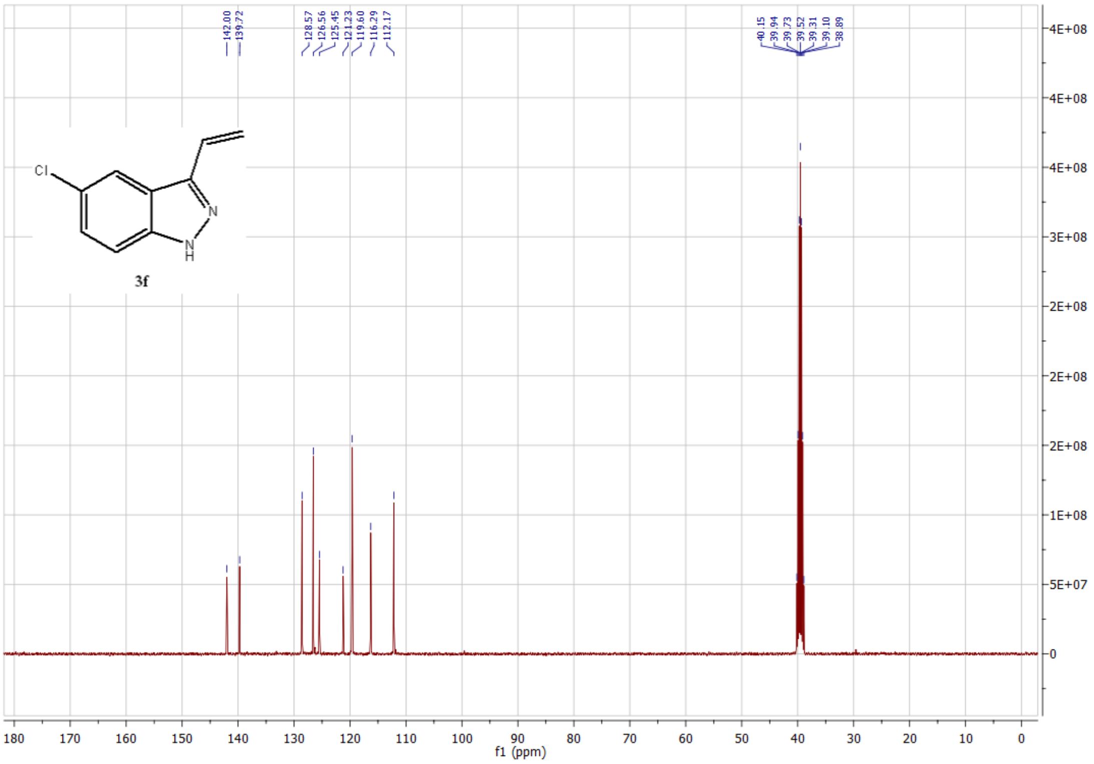

Figure S40. ^{13}C-NMR spectrum of **3f**

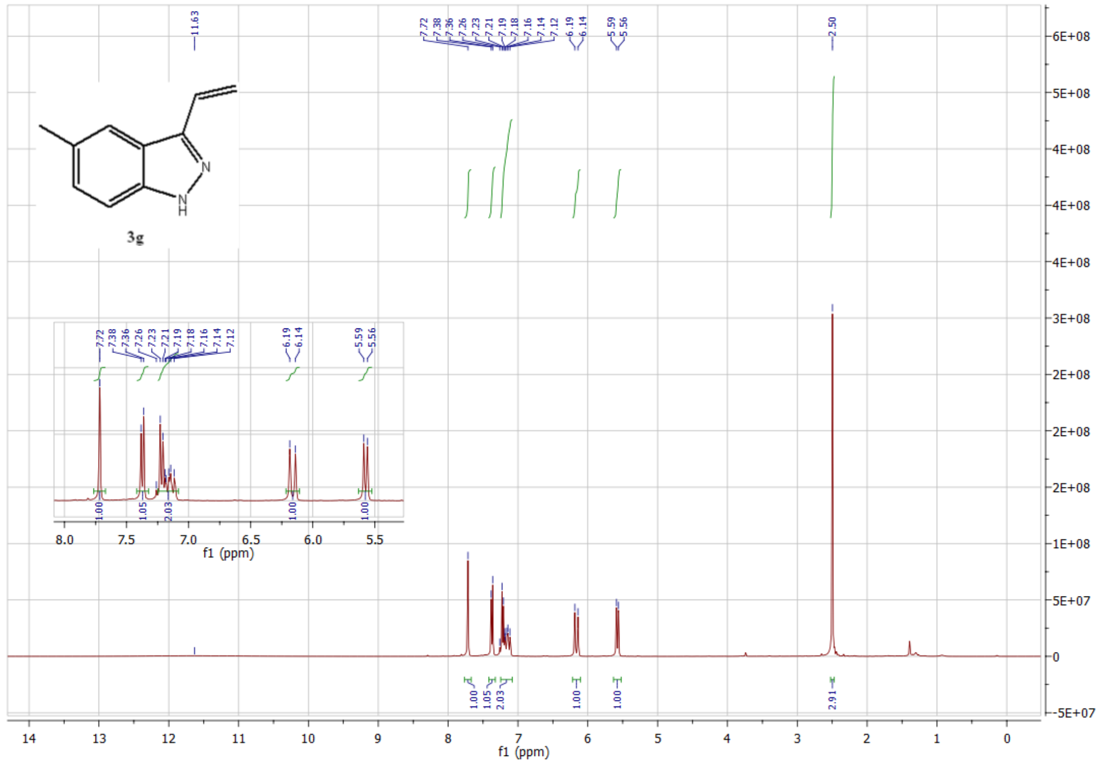

Figure S41. ^{1}H-NMR spectrum of **3g**

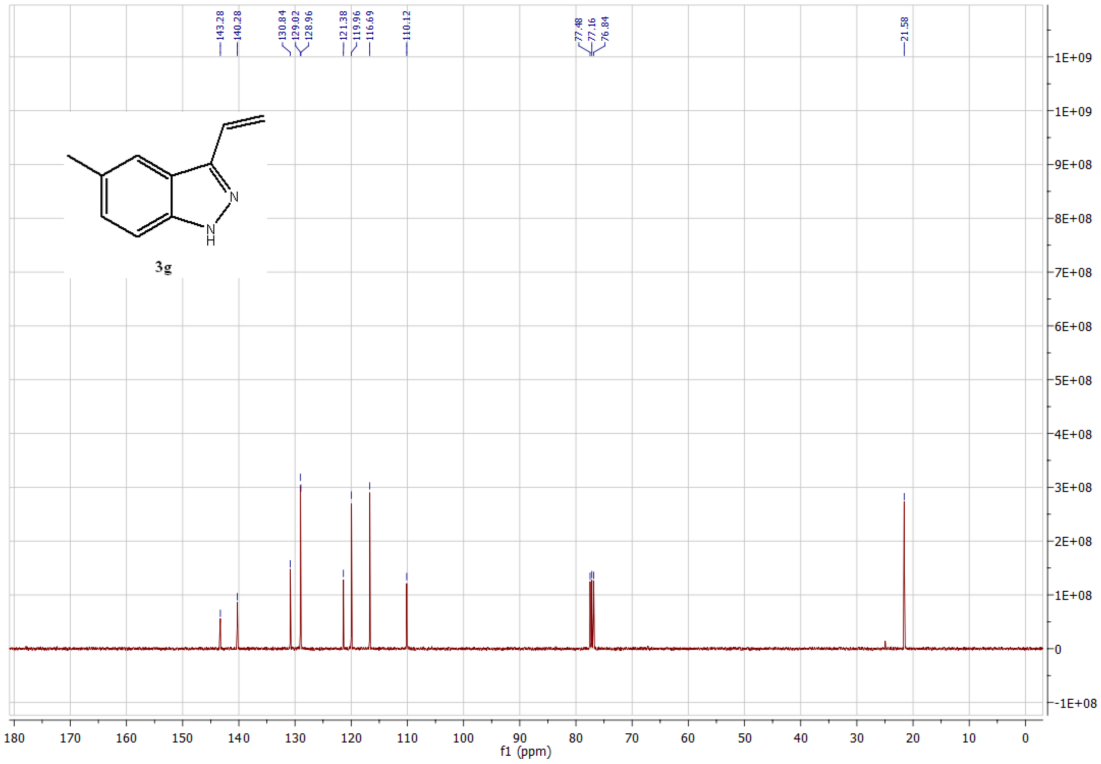

Figure S42. ^{13}C-NMR spectrum of **3g**

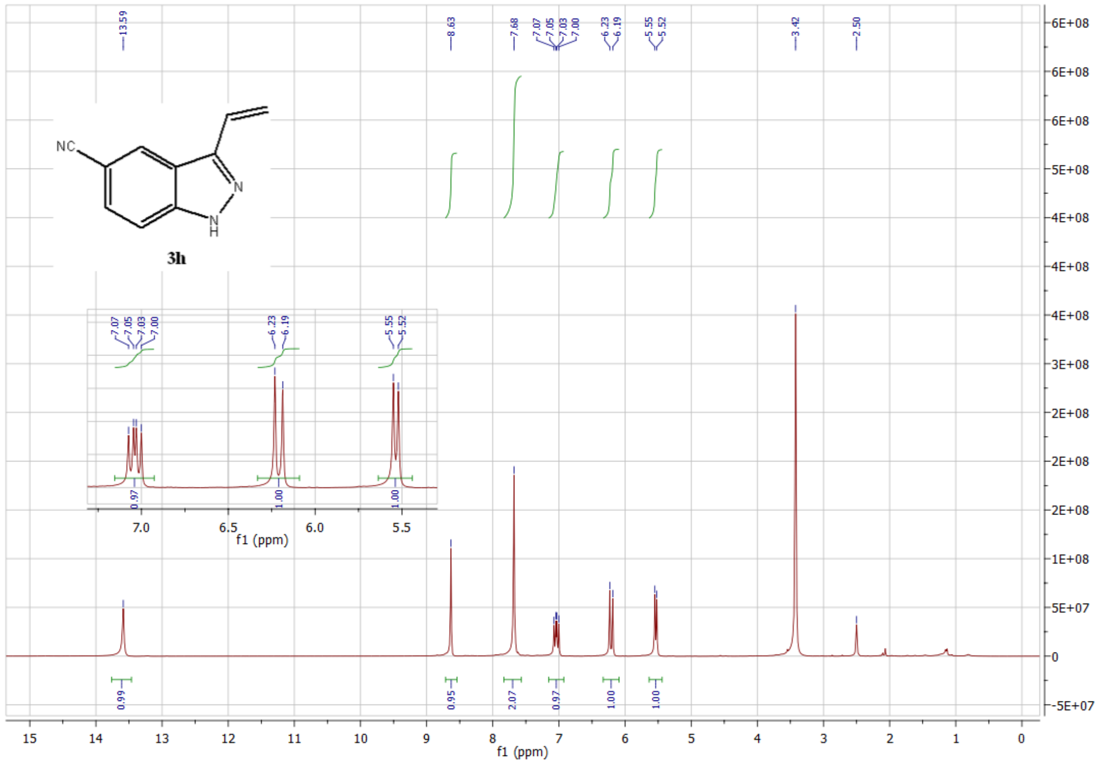

Figure S43. ^{1}H-NMR spectrum of **3h**

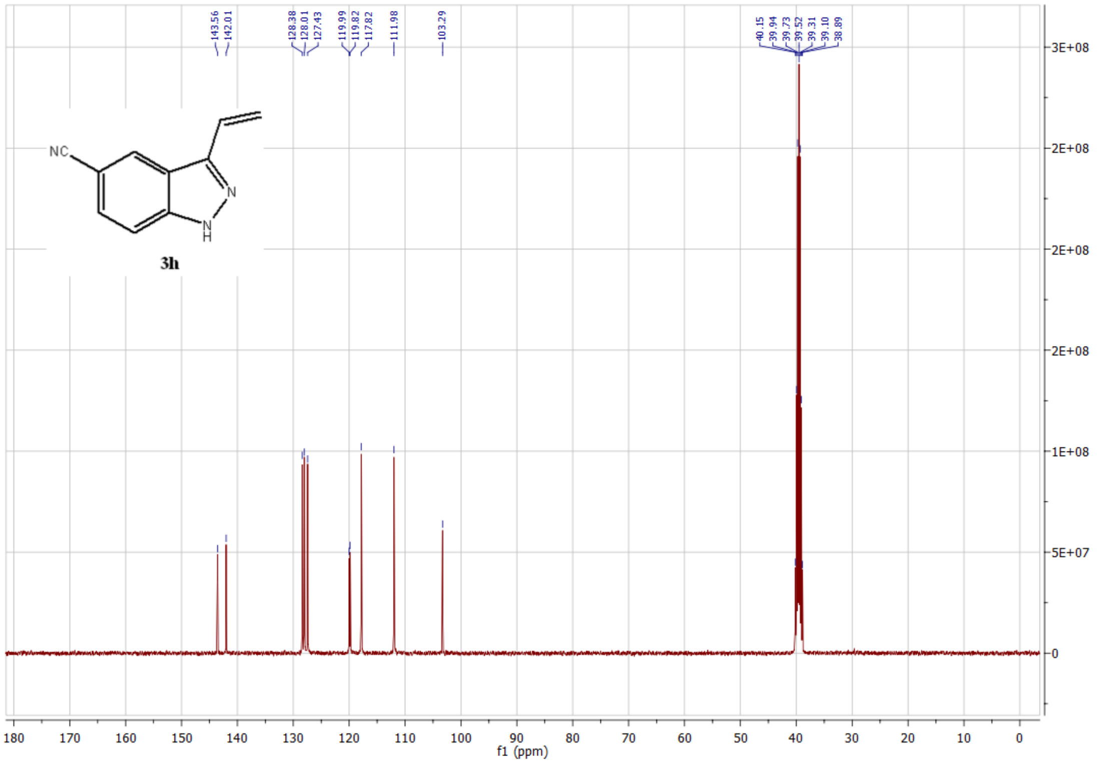

Figure S44. ^{13}C-NMR spectrum of **3h**

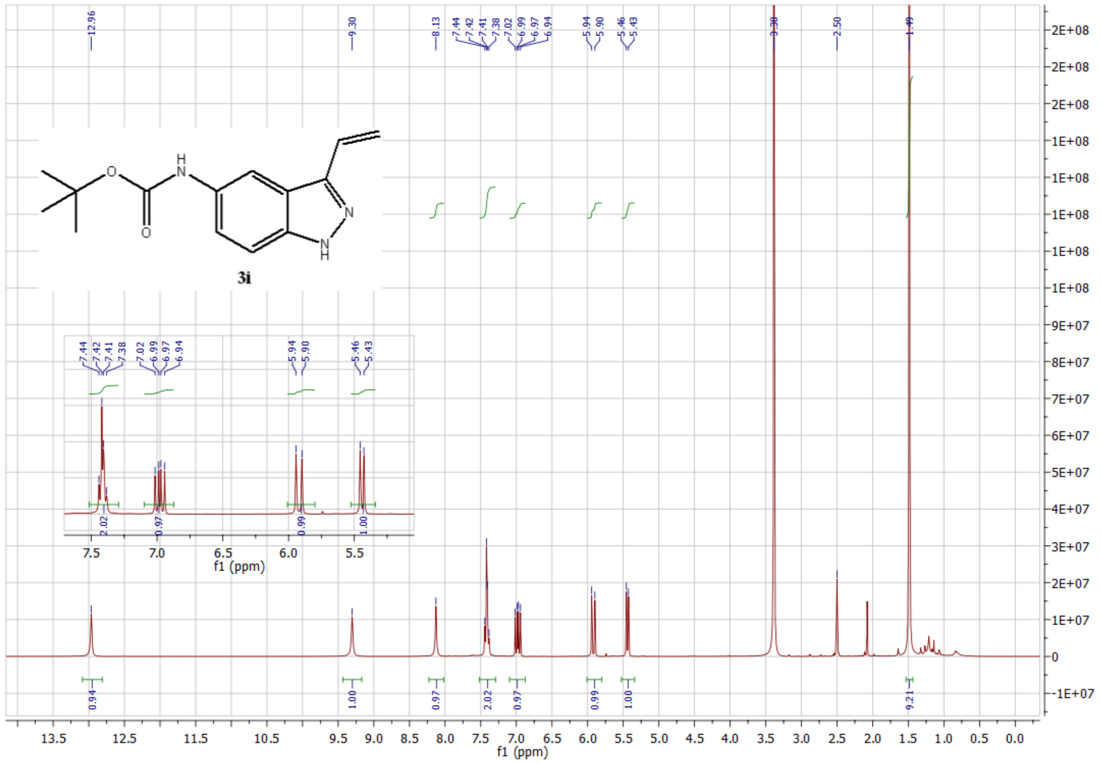

Figure S45. ^{1}H-NMR spectrum of **3i**

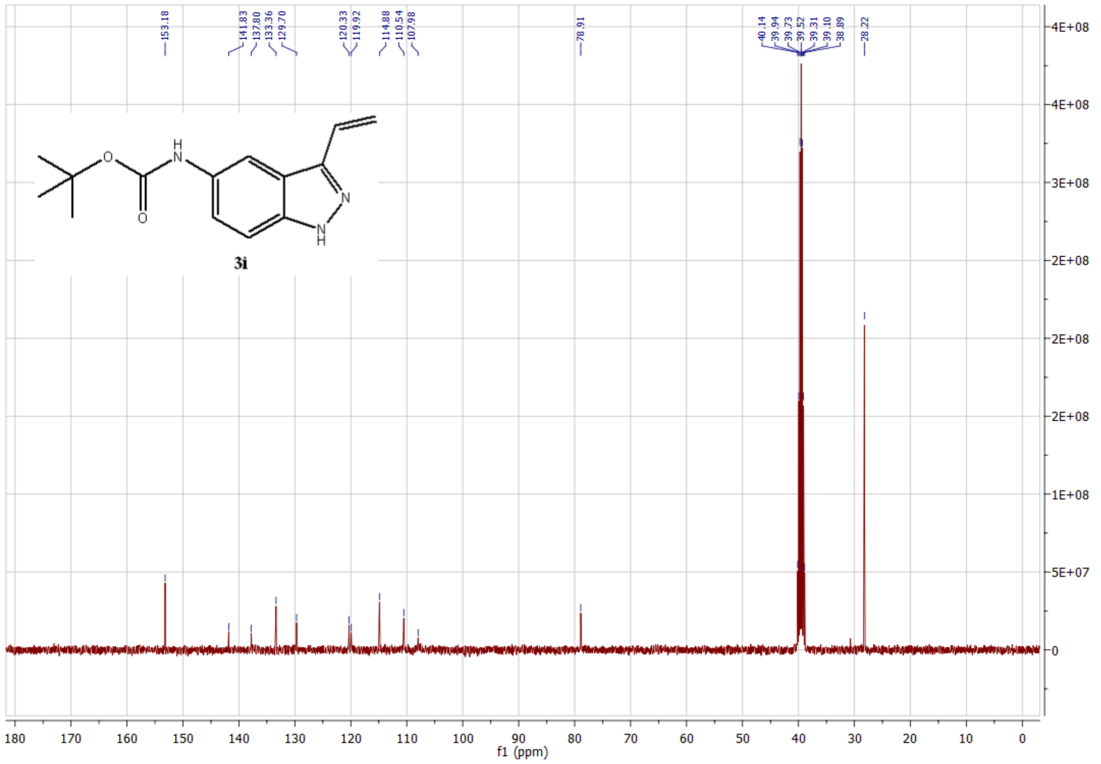

Figure S46. ^{13}C-NMR spectrum of **3i**

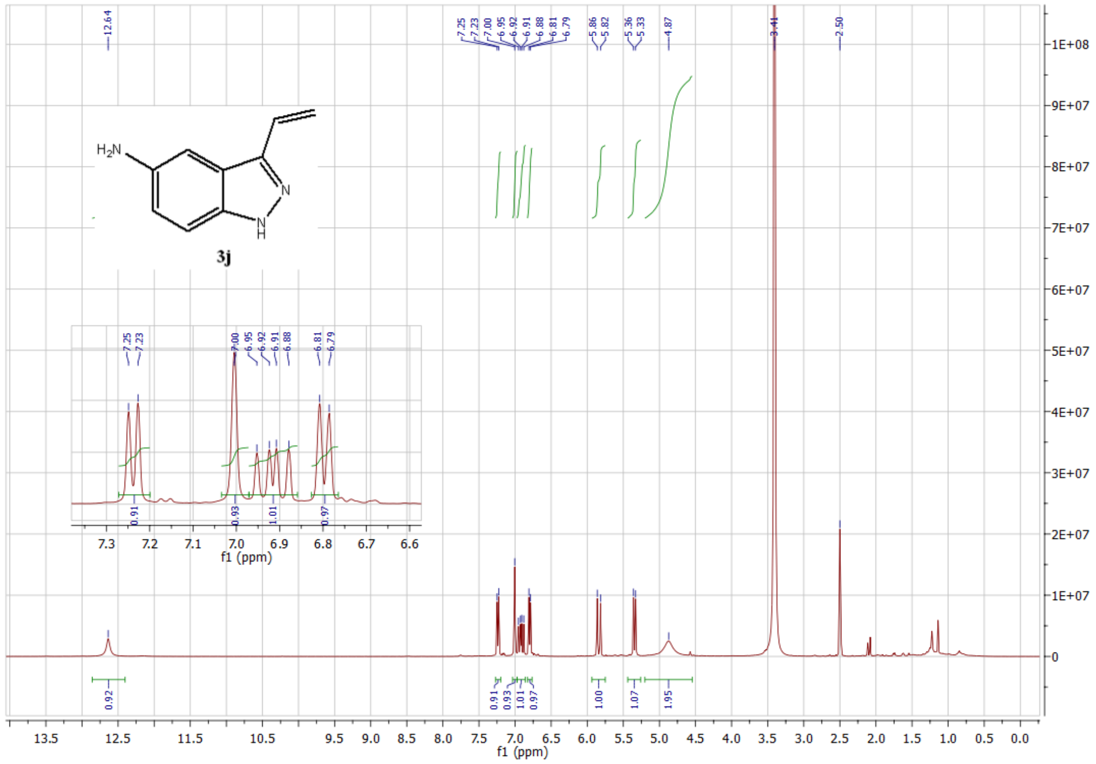

Figure S47. ^{1}H-NMR spectrum of **3j**

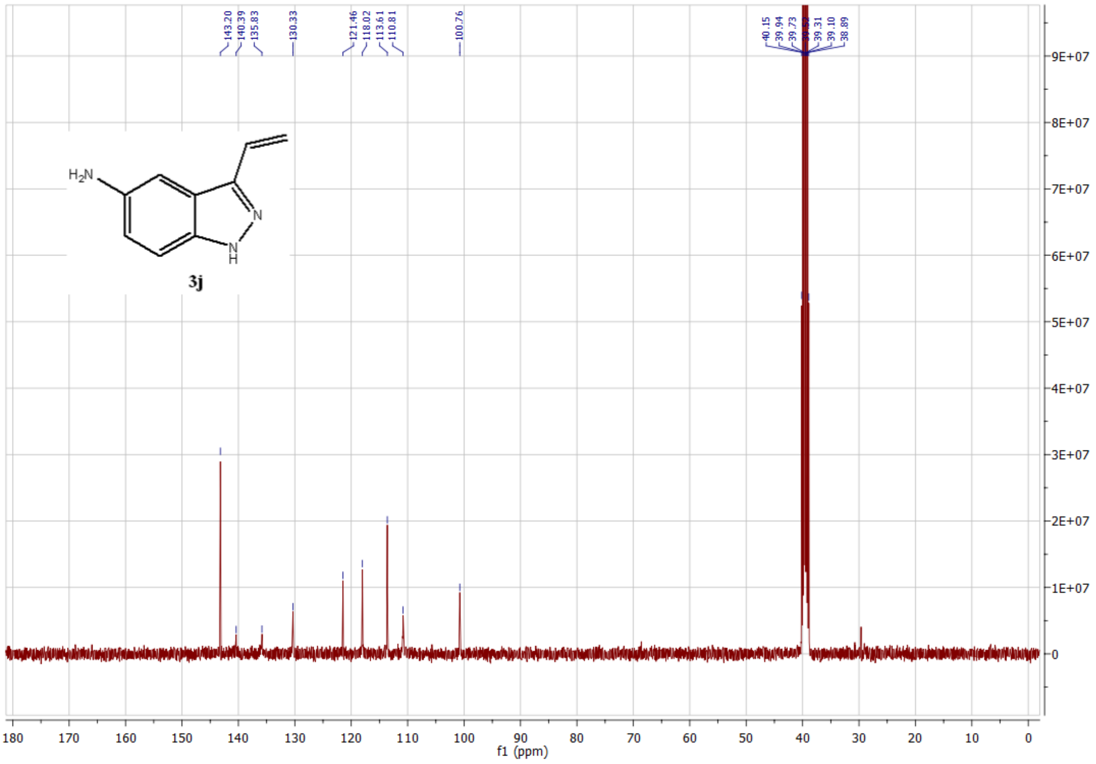

Figure S48. ^{13}C-NMR spectrum of **3j**
